# Using test positivity and reported case rates to estimate state-level COVID-19 prevalence and seroprevalence in the United States

**DOI:** 10.1371/journal.pcbi.1009374

**Published:** 2021-09-07

**Authors:** Weihsueh A. Chiu, Martial L. Ndeffo-Mbah

**Affiliations:** 1 Department of Veterinary Integrative Biosciences, College of Veterinary Medicine and Biomedical Sciences, Texas A&M University, College Station, Texas, United States of America; 2 Department of Epidemiology and Biostatistics, School of Public Health, Texas A&M University, College Station, Texas, United States of America; Institute for Disease Modeling, UNITED STATES

## Abstract

Accurate estimates of infection prevalence and seroprevalence are essential for evaluating and informing public health responses and vaccination coverage needed to address the ongoing spread of COVID-19 in each United States (U.S.) state. However, reliable, timely data based on representative population sampling are unavailable, and reported case and test positivity rates are highly biased. A simple data-driven Bayesian semi-empirical modeling framework was developed and used to evaluate state-level prevalence and seroprevalence of COVID-19 using daily reported cases and test positivity ratios. The model was calibrated to and validated using published state-wide seroprevalence data, and further compared against two independent data-driven mathematical models. The prevalence of undiagnosed COVID-19 infections is found to be well-approximated by a geometrically weighted average of the positivity rate and the reported case rate. Our model accurately fits state-level seroprevalence data from across the U.S. Prevalence estimates of our semi-empirical model compare favorably to those from two data-driven epidemiological models. As of December 31, 2020, we estimate nation-wide a prevalence of 1.4% [Credible Interval (CrI): 1.0%-1.9%] and a seroprevalence of 13.2% [CrI: 12.3%-14.2%], with state-level prevalence ranging from 0.2% [CrI: 0.1%-0.3%] in Hawaii to 2.8% [CrI: 1.8%-4.1%] in Tennessee, and seroprevalence from 1.5% [CrI: 1.2%-2.0%] in Vermont to 23% [CrI: 20%-28%] in New York. Cumulatively, reported cases correspond to only one third of actual infections. The use of this simple and easy-to-communicate approach to estimating COVID-19 prevalence and seroprevalence will improve the ability to make public health decisions that effectively respond to the ongoing COVID-19 pandemic.

## Author summary

Timely and reliable estimates of COVID-19 prevalence and seroprevalence are paramount for evaluating the spread and control of the pandemic in different US states. Relying on reported cases and test positivity rates individually can result in incorrect inferences as to the spread of COVID-19 and ill-informed public health decision-making. Our study developed a simple semi-empirical model for estimating state-level prevalence and seroprevalence of COVID-19 in the United States (US) using reported case and test positivity rates data. We found that due to the preferential nature of diagnostic COVID-19 testing in the US, the geometric mean of reported case and test positivity rates is an accurate predictor of undiagnosed COVID-19 prevalence and trends.

## Introduction

Accurate and reliable estimates of the prevalence and seroprevalence of infection are essential for evaluating and informing public health responses and vaccination strategies to mitigate the ongoing COVID-19 pandemic. The gold standard method to empirically measure disease prevalence and seroprevalence is to conduct periodic large-scale surveillance testing via random sampling [1]. However, this approach may be time- and resource-intensive, and only a handful of such surveillance studies has been conducted so far in the United States (US) [2–7]. Therefore, public health officials have relied on alternative metrics, such as test positivity, reported cases, fatality rates, hospitalization rates, and epidemiological models’ predictions, to inform COVID-19 responses. Test positivity has, for instance, been commonly used to infer the level of COVID-19 transmission in a population and/or the adequacy of testing [8–14]. However, the justifications for use of this metric often reference a WHO recommendation intended to be applied only in a sentinel surveillance context [15]), rather than in the more general context in which it has been frequently implemented. As measures of prevalence, test positivity and reported cases, although readily available and well-understood by public health officials, are very likely to provide biased estimates of disease transmission/prevalence and seroprevalence [1,16,17]). Hospitalization and death rates are also similarly readily available, but tend to lag infections by several weeks and only reflect the most severe outcomes [1]. Finally, epidemiological models are generally complex mathematical, computational, or statistical models that require extensive data and information for model training, and are perceived as a “black box” by most public health practitioners and decision makers [18–20].

**Fig 1:**
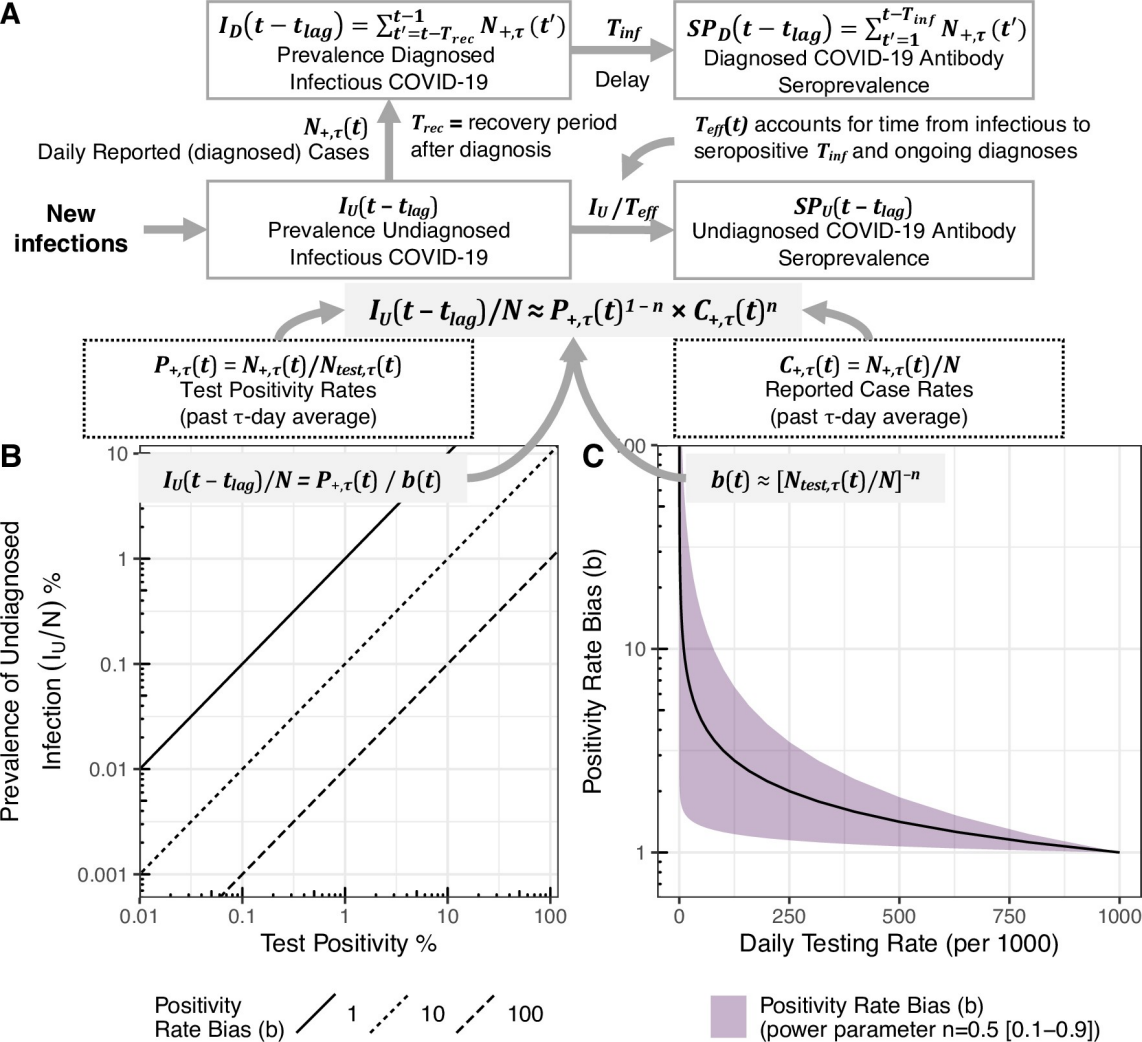
Conceptual model for relationship between test positivity, prevalence of infection, and testing rate. **(A)** Compartmental representation of how the relationships between new infections, undiagnosed and diagnosed prevalence (*I*_*U*_ and *I*_*D*_) and seroprevalence (*SP*_*U*_ and *SP*_*D*_) are modeled for each state, given a bias with power *n*. All observational inputs are the past *τ*-day averages of number of positive tests *N*_*+*,*τ*_(*t*) and number of tests performed *N*_*test*,*τ*_(*t*), the corresponding test positivity rate *P*_*+*,*τ*_(*t*) and reported case rate *C*_*+*,*τ*_(*t*), and the state population size *N*. For diagnosed prevalence and seroprevalence, the observational input is the daily reported cases *N*_*+*,*τ*_, and the model parameters are the recovery time after diagnosis *T*_*rec*_ and the time from infection to seropositivity *T*_*inf*_. For undiagnosed prevalence and seroprevalence, our model assumes the test positivity rate is correlated to delayed undiagnosed disease prevalence with a bias parameter *b*(*t*) modeled as a negative power function of the testing rate *b*(*t*) = [*N*_*test*,*τ*_(*t*)/*N*]^–*n*^ (Eq 2). The additional parameters consist of the power parameter *n* and the initial (missed) seroprevalence *SP*_o_. The effective rate parameter 1/*T*_*eff*_ is time-dependent, and accounts for both *T*_*inf*_ and ongoing diagnoses so as to not “double count.” Prevalence and seroprevalence are evaluated with a lag time *t*_*lag*_, assumed equal to half the averaging time *τ*/2. In **(B**), the diagonal lines represent different values of the bias parameter. In **(C),** the relationship between testing rate and bias parameter represented by Eq (4) is illustrated. Here the shaded region represents different powers *n* ranging from 0.1 (lower bound bias) to 0.9 (upper bound bias), the solid line represents *n* = ½.

Here, we develop a simple semi-empirical model to estimate the undiagnosed prevalence and seroprevalence of COVID-19 at the US state level based only on reported cases, test positivity rate, and testing rate (**Fig 1**). Specifically, we hypothesized that passive case finding employed in the US leads to preferential diagnostic testing for individuals at higher risk of infection and can be modeled as a convex function of the overall testing rate, reflecting the “diminishing return” from expanding general population testing (**Fig 1B and 1C**). We modeled this convexity using a negative power function, with power parameter *n* that is either fit to each state (random effects model) or fixed at ½ (geometric mean model). We also included seroprevalence in our simple semi-empirical modeling framework by adding an offset term *SP*_o_ to account for missed infections during the early part of the pandemic before regular and large-scale testing was established. We calibrated and validated the power parameter and other model parameters by fitting our seroprevalence model to state-wide seroprevalence data (**Tables A and B** in S1 Text), which has only recently become available across all U.S. states [2–7,21], using a Bayesian inference approach. We also compared our model predictions against two independent data-driven mechanistic models [18,22,23] and showed that our model’s predictions of infection prevalence approximate those of more complex models. We found that the state-level prevalence of undiagnosed COVID-19 in the US can be well-approximated by a geometric mean (corresponding to *n* ≈ ½) of the reported cases and test positivity rates. We evaluated how overall disease prevalence and seroprevalence varies with changes in reported cases and test positivity, and the implications of applying this simple model on informing public health decision-making to the COVID-19 pandemic in the US.

## Results

### Bayesian calibration to seroprevalence data

Four independent Markov chain Monte Carlo chains were simulated, and reached adequate convergence after 20,000 iterations per chain for the random effects model and 2,000 iterations per chain for the geometric mean model (PSRF ≤ 1.15 for all parameters) (see **Table 1** and **Table C** in S1 Text) and the multivariate PSRF≤1.11. For inference, 2,000 samples were selected randomly from across the available iterations (80,000 for random effects and 8,000 for geometric mean).

**Table 1:** Model parameters, prior and posterior distributions, and convergence diagnostic.

			Random Effects Model		Geometric Mean Model	
Model parameter	Prior distributions or fixed value	Rationale or Source	Posterior median [95% CrI]	PSRF	Posterior median [95% CrI]	PSRF
*n* (power parameter)	μ: Uniform on logit(*n*) Σ: Log-Uniform	Non-informative prior	μ: 0.54 [0.46–0.67] Σ: 0.20 [0.11–0.31]	μ: 1.15 Σ: 1.00	μ: 0.5 (fixed) Σ: 0 (fixed)	N/A
SP_o_ (initial condition for seroprevalence)	μ: Uniform on logit(SP_o_) Σ: Log-Uniform	Non-informative prior	μ: 0.61% [0.18% - 1.20%] Σ: 1.44 [0.99–2.33]	μ: 1.01 Σ: 1.00	μ: 0.90% [0.38% - 1.50%] Σ: 1.38 [1.02–2.01]	μ: 1.04 Σ: 1.01
*T*_inf_ (infection duration in days)	μ: Normal(m = 14, sd = 3.5)	[36]	μ: 11.2 [5.1–17.0]	μ: 1.11	μ: 15.1 [13.1–17.4]	μ: 1.06
σ_err_ (residual standard error on natural log scale)	μ: Log-Uniform	Non-informative prior	μ: 0.27 [0.24–0.30]	μ: 1.00	μ: 0.28 [0.25–0.32]	μ: 1.00
*T*_rec_ (recovery duration after diagnosis in days)	10	[31]				
*τ* (averaging time for smoothing testing data in days)	14 (sensitivity analysis includes 7 and 28)	At least 1 week to smooth out weekend effects.				
*t*_lag_ (lag time for prediction)	*τ*/*2*	Centered averaging window				

μ = fixed effect, Σ = random effect standard deviation (on logit scale), PSRF = potential scale reduction factor convergence diagnostic [33].

The 95% credible intervals for the power parameter *n* include ½ (corresponding to an unweighted geometric mean) both for the fixed effect and for all but three states’ random effects (ME, NH, RI) (**Table C** and **Fig A** in S1 Text). For the seroprevalence offset *SP*_o_, the posterior median for most states was < 1% initial condition, but three states had posterior medians > 5%. Specifically, for NY, PA, and LA, it was estimated that initial cases that were missed constituted 14% [95% CrI: 8.9%-18.6%], 5.2% [0.3%-8.1%], and 5.1% [2.6%-7.7%] of the population, respectively. For NY and LA, these values are consistent with these two states having large initial surges of cases when testing was highly limited, and therefore were likely to have missed a large number of cases. For PA, this value is consistent with its high death-to-case ratio observed in the initial phase of the pandemic which indicates a large number of cases were likely missed [24,25]. The large variation in *SP*_o_ values across states is consistent with high heterogeneity that has been noted both in the size of their initial surge of infections and in their testing capacity and availability.

**Fig 2:**
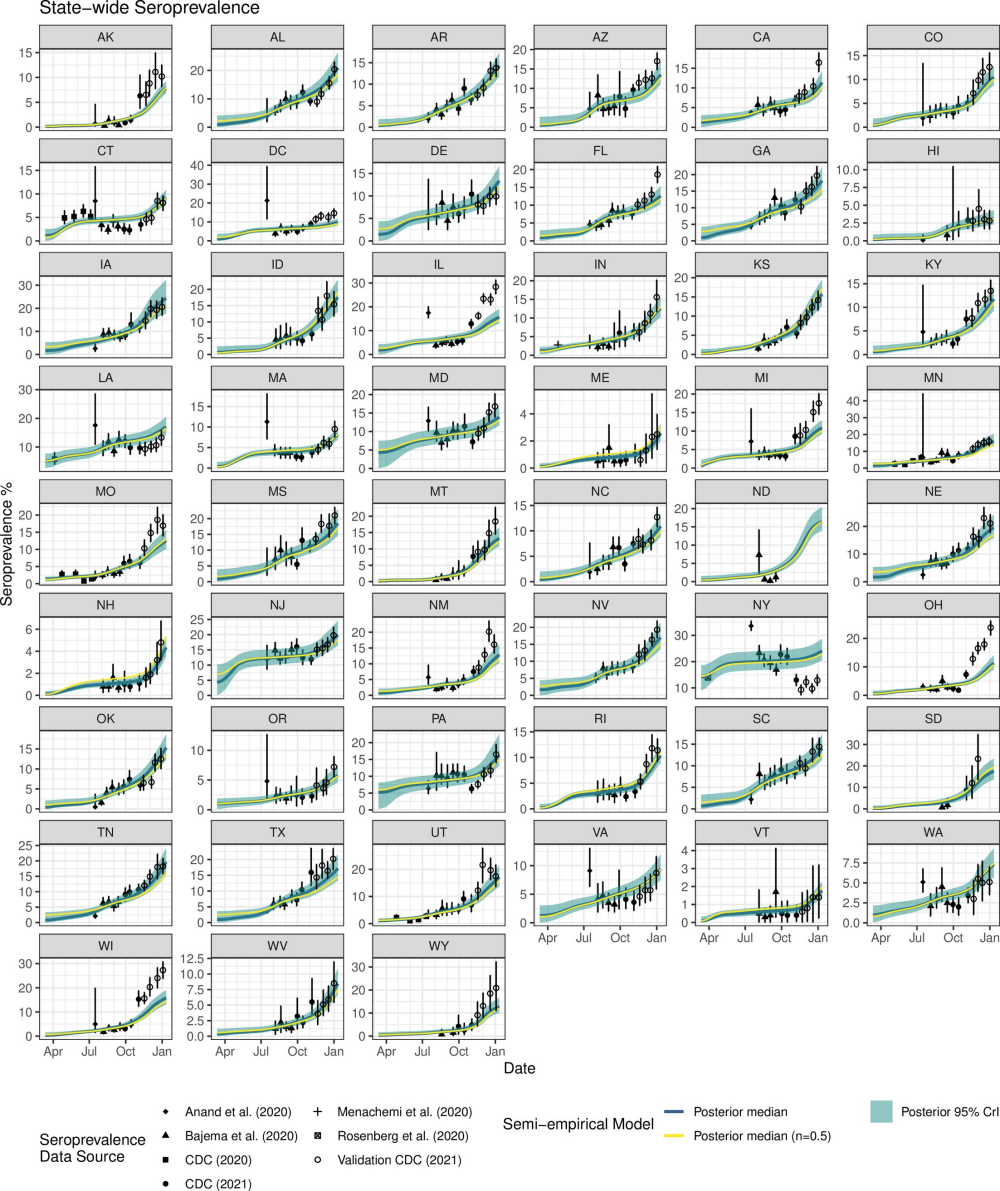
Calibration results of our semi-empirical model for COVID-19 antibody seroprevalence (posterior median and 95% credible intervals for primary random effects model; posterior median only for geometric mean *n* = ½ model) for each state with state-wide seroprevalence data (reported point estimates and 95% confidence intervals shown). Open circles represent validation data not used for model calibration; remaining symbols represent calibration data.

Comparison of posterior estimates and observations by state are shown in **Fig 2**, and show the model to be consistent with available seroprevalence calibration and validation data both in terms of level and trends. For four states (AK, IL, OH, WI), model validation’s predictions underestimated empirical seroprevalence data, though the trends were correctly predicted (**Fig 2**). For calibration data, the residual standard error (RSE) was estimated to be 0.27 [CrI: 0.24–0.30] on the natural log scale, corresponding to a coefficient of variation (CV) of 27% [CrI: 24%-31%], and the R^2^ between the posterior median and the observed point estimates was 0.79 (**Fig B** in S1 Text). Performance for validation data was very similar, with a RSE of 0.30 (corresponding to CV of 31%) and R^2^ of 0.80 (**Fig C** in S1 Text). Calibration and validation seroprevalence predictions had similar accuracy and precision, with the 95% CrI of model predictions within about 3-fold of the observed point estimates (**Figs B and C** in S1 Text).

### Comparison of prevalence estimates with epidemiological models

**Fig 3:**
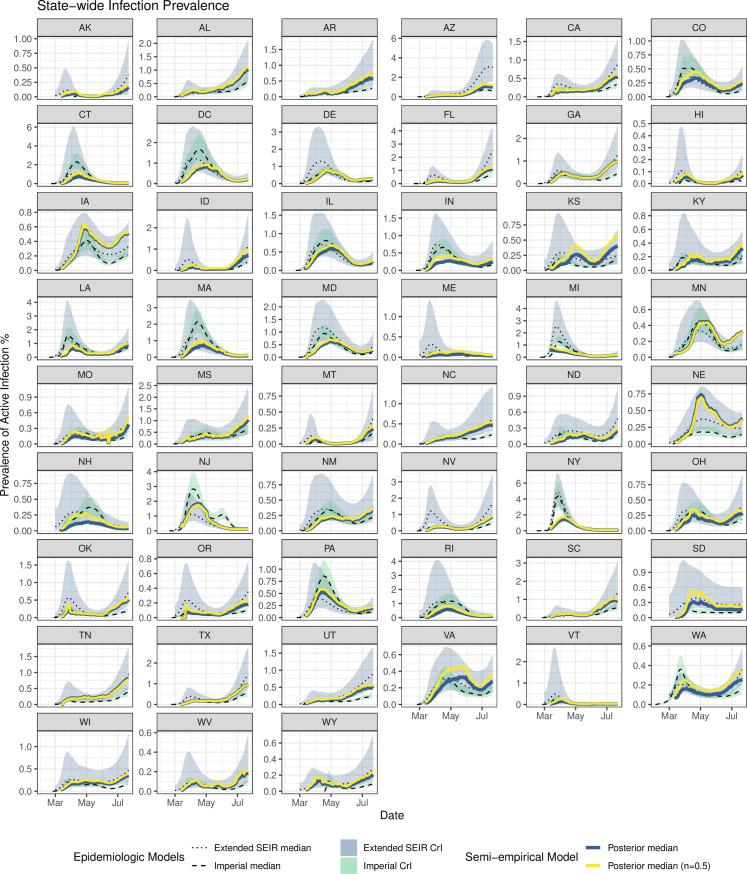
Validation of COVID-19 infection prevalence estimates (posterior median for both primary random effects model and simpler geometric mean *n* = ½ model) for each state in comparison to posterior median estimates and 95% credible intervals from two data-driven epidemiologic models: an extended-SEIR model calibrated to reported cases and confirmed deaths through July 22, 2020 [23] and a semi-mechanistic model calibrated to confirmed deaths through July 20, 2020 by Imperial College [37]).

We compared our model estimates for the prevalence of active infections with those from two independent epidemiologic models of U.S. states. As shown in **Fig 3**, the posterior estimates of the semi-empirical model are largely consistent with posterior credible intervals from the epidemiologic models, with the most notable difference in NY, where the initial surge was underestimated. This is not unexpected because this surge includes the “missed” cases, which for seroprevalence was addressed by the seroprevalence offset *SP*_o_, but which is not included in the prevalence estimates. Across all states in aggregate, the RSE difference between the posterior medians of semi-empirical estimate and the extended SEIR model is 0.67 natural log units (see **Fig D** in S1 Text), corresponding to a CV of 75%, with an R^2^ of 0.68. Similarly, the comparison with the Imperial model yields an RSE of 0.66, corresponding to an 74% CV, and an R^2^ of 0.68. These RSE values should be taken in context of the posterior uncertainty in the epidemiologic models themselves, which have individual reported uncertainties corresponding to CV of 45% and 23% for the extended SEIR and Imperial models, respectively, as well as the differences between the two models, which have a CV of 63%. Thus, the difference between the semi-empirical model and the epidemiological models is not much greater than the difference between the two epidemiologic models themselves. Overall, the semi-empirical model estimate of infection prevalence is consistent with the results of the available epidemiologic models.

### Estimates of prevalence and seroprevalence in 2020

**Fig 4:**
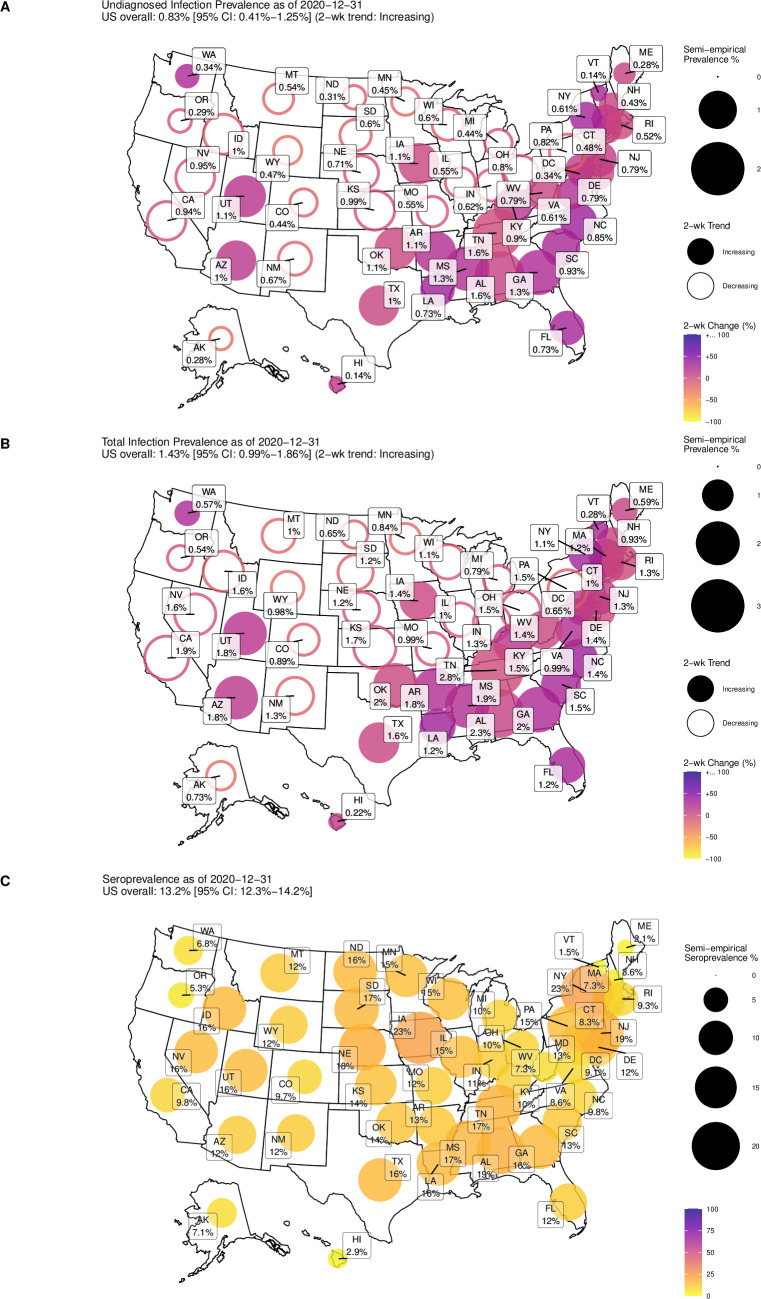
Map of estimated undiagnosed (**A**) and total (**B**) prevalence and transmission trends and overall seroprevalence (**C**) as of December 31, 2020, based on data through January 7, 2021. Values based on primary random effects model. Results for the simpler geometric mean model are provided in **Fig F** in S1 Text. The maps were generated using the R package usmap https://cran.r-project.org/web/packages/usmap/index.html (GPL-3), which uses shape files from the U.S. Census Bureau (the link provided in documentation is here: https://www.census.gov/geographies/mapping-files/time-series/geo/tiger-line-file.html).

As of December 31, 2020, our calibrated and validated semi-empirical model estimates that in the US, total infection prevalence was 1.43% [CrI: 0.99%-1.86%], with more than half undiagnosed (0.83% [0.41%-1.25%]), and a seroprevalence of 13.2% [CrI: 12.3%-14.2%] (**Fig 4**). The simpler geometric mean model gives very similar results (total infection prevalence 1.54%, undiagnosed infection prevalence 0.93%, seroprevalence 12.1% [11.4%-12.9%]) (**Fig F** in S1 Text). In individual states (**Table D** in S1 Text), estimated total prevalence ranged from 0.2% [CrI: 0.1%-0.3%] in Hawaii to 2.8% [CrI: 1.8%-4.1%] in Tennessee, with 3 states (GA, AL, TN) having at least 2% prevalence; undiagnosed prevalence ranged from 0.14% [0.06%-0.25%] in Hawaii to 1.6% [0.6%-2.9%] in Alabama, and was more than 1% in 11 states. The two-week trend in estimated total prevalence was increasing in 27 states and DC (**Fig 4B and Table D** in S1 Text). Estimated seroprevalences in individual states ranged from 1.5% [CrI: 1.2%-2.0%] in Vermont to 23% [CrI: 20%-28%] in New York, with 16 states exceeding 15%, and cumulative reported cases typically accounting for around one in three of estimated total cases (**Fig 4C and Figs E and H and Table D** in S1 Text). Results for the simpler geometric mean model were very similar (**Table E and Figs F and G** in S1 Text).

Between April 1 and December 31, 2020, the test positivity rate bias *b* and the ratio between estimated seroprevalence and cumulative reported cases were shown to decrease over time (**Fig H** in S1 Text). In April, the median positivity rate bias across states was 65 and the cumulative cases underreporting bias ranged between 0.02 to 0.09 (i.e., only 2%-9% of cases were reported). By December, the median positivity rate bias had declined to 17 and the cumulative cases underreporting bias ranged between 0.14 and 0.69 (**Fig H** in S1 Text). Across the U.S. in aggregate, from April to December, the median positivity rate bias declined from 61 to 15, and the cumulative cases underreporting bias improved from 0.01 to 0.33 (**Fig H** in S1 Text). Results for the simpler geometric mean model were very similar.

The pitfalls of relying on reported cases or test positivity rate alone to estimate the course of the epidemic are illustrated for five states, MN, VA, WI, KY, and TN where reported cases and positivity trends were in opposite directions in May or December (**Fig I** in S1 Text). Specifically, in May, reported cases were rising substantially in MN, VA, and WI at the same time that the test positivity rate was declining, testing rate was increasing, and the model (either the primary random effects or the simpler geometric mean) predicted total prevalence was flat or decreasing (**Fig I** in S1 Text). By contrast, in December, the states of KY and TN all showed declining reported case rates while positivity was increasing, while our model predicted that COVID-19 prevalence was actually flat or increasing during this time. In both scenarios, the increase (decrease) in reported cases was due to expanding (declining) testing rates, respectively.

## Discussion

Reported case rates and test positivity rates have been widely used to inform or justify public health decisions, such as increasing or relaxing non-pharmaceutical interventions, for the control of the COVID-19 pandemic in the US [26,27]. A recent report of the National Academies of Sciences and Engineering Medicine (NASEM) has urged caution about the reliability/validity of directly using data such as reported case rates and test positivity rates to inform decision making for COVID-19 [1]. Though these data are usually readily available, the NASEM report concludes that they are likely to substantially underestimate or overestimate the real state of disease spread [1]. Therefore, there is a critical need to develop simple and more reliable data-driven metrics/approaches to inform local public health decision-making.

We have developed a simple semi-empirical approach to estimate the prevalence and seroprevalence of COVID-19 infections in a population using only reported cases and testing rates that does not require developing and maintaining a complex, data-driven mathematical model. Based on a simple hypothesis that the bias in test positivity is a convex, negative power function of the testing rate, we find that the undiagnosed COVID-19 prevalence, with a 1-week lag, is well-approximated by the (weighted or unweighted) *geometric mean* of the positivity rate and the reported case rate averaged over the last 2 weeks (Eq 4). Seroprevalence can be calculated by taking a cumulative sum while accounting for the duration between infection and seropositivity, a period of typically 2 weeks, ongoing diagnoses as reflected in the testing rate, and a state-specific offset accounting for missed infections in the early part of the pandemic prior to establishment of regular testing (Eqs 10 and 11). Our model resulted in an accurate fit to recently available state-level seroprevalence data from across the U.S. Additionally, the prevalence estimates of our semi-empirical model were shown to compare favorably to those from two data-driven epidemiological models. We estimate the nation-wide total prevalence rate as of December 31, 2020 to be 1.4% [CrI: 1.0%-1.9%], corresponding to a test positivity bias of around 15, and nation-wide seroprevalence to be 13.2% [CrI: 12.3%-14.2%], so that cumulative reported cases correspond to approximately one-third of actual past infections. At the state level, estimated seroprevalence was 1.4 to seven times cumulative reported cases. These estimates compare favorably to those previously published using more complicated approaches [28,29].

Our analysis suggests that public health policy related to either non-pharmaceutical (masking and social distancing) or pharmaceutical interventions (vaccination) may be informed by available data in three main ways:

- First, decline in either positivity rate or reported case rates alone is insufficient to infer that prevalence is declining. In the case where one is increasing and one is decreasing, our model suggests that the direction of their geometric mean is a better indicator of increasing or decreasing prevalence (**Fig I** in S1 Text). Reported cases are particularly unreliable indicators when population testing rates are increasing or decreasing substantially, and at low testing rates, when the positivity rate bias is higher.
- Second, reported cases, test positivity, and testing rates should be publicly reported at the county or municipal level in order to provide local governments, health agencies, medical personnel, and the public with the necessary information to evaluate local pandemic conditions. Currently, only reported cases are routinely provided at the local level, with positivity and testing rates aggregated (often inconsistently) only at the state level.
- Finally, seroprevalence estimates can play a key role in forecasting future potential spread of the pandemic and threshold vaccination coverage needed to stamp out disease transmission at the state or community-level.

As with any model, ours has a number of limitations. The most significant limitation is the lack of more comprehensive, random sampling-based data with which to further validate the model. However, our model did accurately fit all the available seroprevalence data, including recent CDC data at multiple time points across all 50 states and the District of Columbia [6]. As further validation of the approach, we applied our model internationally to 15 countries for which both nation-wide seroprevalence data (**Table F** in S1 Text) and daily testing data were available early in the pandemic (March-August). The 95% CrI for our model, using the random effects posterior distributions from our U.S. state-level calibration, covered all the seroprevalence data except for Russia (**Fig J** in S1 Text), suggesting that this approach might be more broadly applicable, though requiring nation-specific calibration. With respect to prevalence, we could only compare epidemiologic model-based estimates of prevalence due to lack of random sample-based surveillance data. However, we believe this limitation is mitigated by our use of two independent estimates with completely different model structures, one of which is a more traditional extended-SEIR model, and other of which is a “semi-mechanistic” model partially statistical in nature. Another important limitation is the relatively limited range of testing rate observations for most U.S. states. For this reason, we cannot necessarily guarantee that our results can be easily extrapolated to substantially higher testing rates. However, with higher testing rates, the difference between test positivity rates and reported case rates would decrease and reduce the effect of greater uncertainty in the degree of bias between the test positivity rate and the lagged prevalence. Our model also does not account for the potential impact of population movement on seroprevalence. In- and out-flow of seropositive individuals could alter a state’s seropositivity rate. While population movement may have marginal impact on COVID-19 seroprevalence in most states/countries because of mobility restrictions, some US states such as New York have experienced a significant increase in population out-flow during the pandemic [30]. This population movement may explain in part the reduction in seroprevalence observed in New York. Moreover, our model did not account for the impact of rates of false-positives and false-negatives on COVID-19 prevalence and how these rates may change with testing methods/strategies. However, if time series data on false-positive and false-negative rates were available, these could be easily incorporated into our modeling framework. We anticipate that the impact of imperfect test accuracy (the sensitivity and specificity of diagnostic testing) is likely to have a minimal impact on our results. Finally, for simplicity, our model assumes the power parameter, *n* for the bias function, *b*(*t*) remain constant during the course of the epidemic. This assumption can be extended by assuming the power term changes as testing behavior and strategies and/or infection prevalence change over time. This can be done by using a stepwise function with *n*′*s* value constant over periods of marginal changes in testing strategies and behavior. Future work can account for these different factors and could also extend the current framework to explicitly account for the impact of vaccination on estimating disease prevalence and seroprevalence.

In conclusion, we found that the undiagnosed COVID-19 prevalence is well-approximated by the *geometric mean* of the positivity rate and the reported case rate, and that seroprevalence can be estimated by taking a cumulative sum while accounting for the duration between infection and seropositivity, a period of typically 2 weeks, ongoing diagnoses, and a state-specific offset. The use of this simple, reliable, and easy-to-communicate approach to estimating COVID-19 prevalence and seroprevalence will improve the ability to make public health decisions that effectively respond to the ongoing COVID-19 pandemic in the U.S.

## Methods

### Conceptual basis of a discrete-time semi-empirical model for the prevalence of COVID-19 infection

First, we develop a model for infection prevalence. Test positivity rate *P*_+,*τ*_(*t*) = *N*_+,*τ*_(*t*)/*N*_*test*,*τ*_(*t*) is defined as the percentage of positive diagnostic tests administered over a given period *τ* between *t*−*τ* and *t*, where time *t* is discretized by day (we use τ-averaged testing data throughout our analysis to smooth out day-to-day variations in reporting, including weekend effects). We hypothesize that, because testing is mainly done through passive case finding (i.e., only those considered more likely to be infected due to symptoms, contacts, etc., are tested), *P*_+,*τ*_(*t*) is correlated to the lagged prevalence *I*_*U*_(*t*−*t*_*lag*_)/*N* of undiagnosed COVID-19-infected persons in the population, where *N* is the population size, with a time-dependent bias parameter *b*(*t*):

$$P_{+,\tau }(t)=b(t)\times \frac{I_{U}(t-t_{lag})}{N}$$ (1)

Conceptually, this relationship is shown in **Fig 1A and 1B**. As shown in **Fig 1C**, we also hypothesize that the bias parameter *b*(*t*) is inversely related to the testing rate *Λ*_*τ*_(*t*) = *N*_*test*,*τ*_(*t*)/*N* over the same period *τ*. At a testing rate of 1, where everyone is tested, there is no bias, so *b* = 1. On the other hand, for low testing rates, the bias is likely to be high, as mostly severely ill individuals will be tested. We assume large-scale passive testing as a baseline testing rate for our model, which is consistent with COVID-19 outbreak response in the US. Under this condition, increases in the testing rate from baseline, which reflects more active testing/contact tracing efforts, will preferentially increase the infected population testing rate relative to the general population testing rate; so *b*(*t*) may decline more rapidly than at higher testing rates, as there is “diminishing return” from increased testing. Thus, for simplicity, we assume that *b*(*t*) is a convex function of *Λ*_*τ*_(*t*).We therefore model the bias as a negative power function of *Λ*_*τ*_(*t*):

$$b(t)={\left[\frac{N_{test,\tau }(t)}{N}\right]}^{-n}\equiv \Lambda_{\tau }(t{)}^{-n}$$ (2)

with *n* restricted between 0 and 1. Though other more complex functional forms could be used, the inverse power function we chose has the advantage that the limit of *n* = 0 reflects no bias (random sampling) and the limit *n* = 1 reflects the case that everyone infected is tested. While this appears to imply an unbounded bias as the testing rate goes to zero, as shown below, our model will naturally limit the bias parameter when test positivity is 100%. Combining Eqs (1) and (2), and re-arranging leads to the following relationship between test positivity and the undiagnosed infectious population:

$$\frac{I_{U}(t-t_{lag})}{N}=P_{+,\tau }(t)\times \Lambda_{\tau }(t{)}^{n}$$ (3)

Additionally, because test positivity and the testing rate share a term *N*_*test*,*τ*_(*t*), Eq (3) can be further rearranged as

$$\frac{I_{U}(t-t_{lag})}{N}=P_{+,\tau }(t{)}^{1-n}{\left[\frac{N_{+,\tau }(t)}{N}\right]}^{n}\equiv P_{+,\tau }(t{)}^{1-n}\times C_{+,\tau }(t{)}^{n}$$ (4)

where the last term is the reported cases per capita *C*_+,*τ*_(*t*) = *N*_+,*τ*_(*t*)/*N*. Thus, our hypothesis predicts that the infectious population is proportional to a *weighted geometric mean* of the positivity rate and the reported case rate, with *n* = ½ corresponding to equal weighting (simple geometric mean). For *n* = 1 the reported cases per capita is equal to the lagged undiagnosed prevalence rate regardless of the underlying disease dynamics and prevalence in the population. Such a scenario will likely occur only when everyone is tested.

To obtain the overall infection prevalence, we need to add diagnosed infectious cases. We assume a recovery period after diagnosis of *T*_rec_ = 10 days, consistent with the CDC quarantine recommendation for COVID-19 infection [31], so the diagnosed cases from the last *T*_rec_ days constitute the active diagnosed infections *I*_*D*_:

$$I_{D}(t{-t}_{lag})=\sum_{t-T_{rec}<t^{\prime }<t}N_{+,\tau }(t\prime ).$$ (5)

Note that *t*′ = *t* is not included because on the day individuals are diagnosed, they are considered part of the undiagnosed prevalence (i.e., testing is “sampling without replacement” of the undiagnosed population).

We can also rearrange Eq (1) and view the bias parameter as the relative efficacy of testing infected individuals compared to the general population:

$$b(t)=\frac{N_{+,\tau }(t)/I_{U}(t-t_{lag})}{N_{test,\tau }(t)/N}\equiv \frac{\Lambda_{I,\tau ,t_{lag}}(t)}{\Lambda_{\tau }(t)}$$ (6)

Here, $\Lambda_{I,\tau ,t_{lag}}(t)$ is the daily rate of testing of infectious individuals (with averaging time *τ* and lag *t*_*lag*_), whereas *Λ*_*τ*_(*t*) is the daily rate of testing of the general population, as previously defined. Thus, the bias reflects the extent to which infectious individuals are “preferentially” tested through passive case finding. Moreover, due to the way *I*_*U*_(*t*−*t*_*lag*_) is calculated, when positivity is 100% so that *N*_+*τ*_(*t*) = *N*_*test*,*τ*_(*t*), the bias appropriately equals *N*/*I*_*U*_(*t*−*t*_*lag*_).

We use this semi-empirical model for infection prevalence to estimate undiagnosed seroprevalence SP_U_(*t*) as follows. Assuming a time interval between infection and seropositivity = *T*_inf_, each time point *t*, we can subdivide the undiagnosed infection prevalence *I*_*U*_ into *T*_inf_ “sub-compartments” *I*_*U*,*m*_ (*m* = 1…*T*_inf_) (see **Fig K** in S1 Text). Given the daily testing rate of infectious individuals $\Lambda_{I,\tau ,t_{lag}}(t)$, the number of individuals in subsequent subcompartments declines by a factor (1 – *Λ*) as diagnoses occur (leaving *I*_*U*_ for *I*_*D*_), so the sub-compartment sizes are:

$$I_{U,m}=I_{U}\frac{{\left(1-\Lambda \right)}^{m-1}}{\sum_{m\prime =1}^{T_{inf}}{\left(1-\Lambda \right)}^{m\prime -1}}.$$ (7)

Thus, the number of undiagnosed individuals who become newly undiagnosed seropositive each day is simply the number in the last sub-compartment $I_{U,T_{inf}}$ multiplied by another factor of (1 – *Λ*), which simplifies to

$${SP}_{U}(t)={SP}_{U}(t-1)+I_{U}(t-1)/\sum_{m\prime =1}^{T_{inf}}{\left(1-\Lambda_{I,\tau ,t_{lag}}(t-1)\right)}^{-m\prime }.$$ (8)

Setting *γ* = 1/(1 – *Λ*), replacing the summation with the formula for the sum of a geometric sequence (*T*_inf_ terms, first term and common ratio both = *γ*), and defining the sum as a time-dependent “effective” time $T_{eff}=\gamma (1-\gamma^{T_{inf}})/(1-\gamma )$, Eq (8) becomes

$${SP}_{U}(t)={SP}_{U}(t-1)+I_{U}(t-1)/T_{eff}(t-1).$$ (9)

Therefore the fraction of *I*_*U*_ becoming seropositive each day (while remaining undiagnosed) is a fraction 1/*T*_*eff*_ (see **Fig K** in S1 Text). As testing rates approach 0, so that virtually everyone remains undiagnosed, this fraction approaches 1/*T*_*inf*_, as would be calculated considering *I*_*U*_ as a single “well-mixed” compartment. Additionally, as an initial condition, we allow for an offset *SP*_o_ for missed infections during the early part of the pandemic before regular and large-scale testing was established. Therefore, combining with Eq (4) gives the undiagnosed seroprevalence rate as:

$$\frac{SP_{U}(t-t_{lag})}{N}=\frac{SP_{o}}{N}+\sum_{t^{\prime }<t}\frac{p_{+,\tau }(t^{\prime }{)}^{1‐n}\times C_{+,\tau }{\left(t^{\prime }\right)}^{n}}{T_{eff}(t^{\prime })}$$ (10)

For the diagnosed seroprevalence *SP*_*D*_, we make the simplifying assumption that it is equal to the cumulative reported cases lagged by the mean time interval between infection and seropositivity *T*_inf_

$$SP_{D}(t-t_{lag})=\sum_{t^{\prime }<t-T_{inf}}N_{+,\tau }(t^{\prime }).$$ (11)

Eqs (4), (5), (10) and (11) therefore comprise the complete semi-empirical model for overall infection prevalence (*I* = *I*_*D*_ + *I*_*U*_) and seroprevalence (*SP* = *SP*_*D*_ + *SP*_*U*_) based solely on average positivity *P*_+,*τ*_(*t*), averaged reported case rates *C*_+,*τ*_(*t*), and corresponding reported cases *N*_+,*τ*_(*t*), which we calculate from data obtained from the COVID Tracking Project [32]. We fix the averaging time *τ* at 14 days, and the lag time *t*_*lag*_ = *τ*/2 at 7 days, so the semi-empirical model has only three remaining free parameters: the power parameter *n*, the infection-to-seropositive time interval *T*_inf_, and the initial condition for seroprevalence *SP*_*o*_. We consider two variations of the model: the primary “random effects” model in which *n* and *SP*_*o*_ are considered as random effects across states and a simpler “geometric mean” model with a fixed *n* = ½ so that *I*_*U*_ is the geometric mean of positivity and case rates. For both variations, a single value of *T*_inf_ across states is used. We conducted sensitivity analyses for different values of the averaging time *τ* (7 and 28 days instead of 14 days); the posterior parameter estimates for *n* and *SP*_*o*_ and the seroprevalence predictions were almost indistinguishable across different averaging times, while the infection prevalence predictions were much noisier using an averaging time of 7 days but little changed using 28 days (**Figs L-O** in S1 Text).

### Bayesian calibration and validation using seroprevalence data

To calibrate and validate the model, we utilized state-wide seroprevalence data, which has only recently become available for all 50 states and the District of Columbia (**Table A** in S1 Text). Specifically, we fitted our model using data collected from 9-March-2020 to 15-Nov-2020 and validated our model predictions by comparing them to data collected from 9-Nov-2020 to 4-Jan-2021 that were not used for model fitting (the overlap in dates is due to overlapping end dates and start dates of CDC data collection rounds). The likelihood function assumes independent log-normal distributed errors given an observed and model-predicted seroprevalence. The log-transformed variance of the likelihood distribution was calculated as the sum of the reported error variance in the data (estimated from reported 95% CI for each observation) and a fitted residual error variance. We used a Bayesian MCMC approach to calibrate the model parameters (see **Table 1** for prior and posterior distributions) and the potential scale reduction factor (PSRF) was used to assess convergence, with a value of <1.2 regarded as adequate [33,34]. Additional details about model calibration and validation are found in S1 Text.

### Comparison of prevalence estimates with epidemiological models

We compare prevalence estimates of our model to estimates from a Bayesian extended-SEIR [23] and Imperial model [35]. This was done by comparing the log-transformed posterior median estimates for each model for their overlapping time intervals (March 12 to July 22 for the extended-SEIR model and March 12 to July 20 for the Imperial model). The model performance was quantified by the residual standard error on the log-transformed values between models, the corresponding coefficient of variation, as well as the R-squared statistic. The extended-SEIR [23] was calibrated to US state-level reported cases and deaths through a Markov Chain Monte Carlo (MCMC) approach using a Metropolis within Gibbs sampling. The model explicitly estimated underreported symptomatic/mild symptomatic cases in each state and the District of Columbia. The Imperial model [35] uses a Bayesian semi-mechanistic model calibrated to US state-level reported deaths. Model calibration was done using a MCMC approach with an adaptive Hamiltonian Monte Carlo (HMC) sampler. The model back-calculates cases from estimated deaths through estimated infection fatality rate. This approach implicitly accounts for under-reported cases. Both of these are Bayesian models, and we use these models’ posterior distributions for comparison.

### Bias of test positivity and reported cases in estimating prevalence and seroprevalence

Our model can be used to estimate the degree of bias in current measures of prevalence (test positivity and reported case rates) and seroprevalence (cumulative reported cases). The over-reporting bias of test positivity as a measure of prevalence is already given in Eq (2). The under-reporting bias of reported case rates can be calculated by rearranging Eq (4),

$$C_{+,\tau }(t)=\frac{I_{U}(t-t_{lag})}{N}\times {\left[\frac{N_{test,\tau }(t)}{N}\right]}^{1-n}=\frac{I_{U}(t-t_{lag})}{N}\times {b(t)}^{(n-1)/n},$$ (12)

so the under-reporting bias is *b*(*t*)^(*n*−1)/*n*^, which is equal to *b*(*t*)^−1^ for *n* = ½. The implied bias from cumulative reported cases as a measure of seroprevalence is calculated by dividing by sum of *C*_+,*τ*_(*t*) by the seroprevalence estimated by Eqs (10 and 11).

### Software

All analyses were performed using the R statistical software (R version 3.6.1) in RStudio (Version 1.2.1335). We have implemented our model in an online dashboard (https://wchiu.shinyapps.io/COVID-19-Prevalence-and-Seroprevalence/) to enable easy access to our results.

### Ethical approval

Ethical approval was not required for this work.

## Supporting information

S1 TextSupplemental Methods.**Table A.** State-wide seroprevalence calibration data. **Table B.** State-wide seroprevalence validation data. **Table C.** Posterior distributions and convergence diagnostic of *n* and SP_o_ for individual states (random effects). **Table D.** Primary model posterior estimates of prevalence (undiagnosed and total) and seroprevalence as of December 31, 2020. **Table E.** Geometric mean model posterior estimates of prevalence (undiagnosed and total) and seroprevalence as of December 31, 2020. **Table F.** International seroprevalence data. **Fig A.** Posterior distributions of the power parameter *n* and the seroprevalence offset SP_o_ for individual states using the primary random effects model. The fixed effect is denoted by “F.E.,” and the vertical dashed line represents its posterior median. For the simpler geometric mean model, the power parameter is fixed at *n* = ½, and the F.E. posterior median [CrI] for SP_o_ is 0.90 [0.38–1.50]. **Fig B.** Scatter plot of seroprevalence predictions (posterior median for primary random effects model) versus calibration data (reported point estimate and 95% CI). The solid line represents equality, the dashed line is +/- one residual standard error, and the dotted line is the 95% CrI residual error. The adjusted R^2^ is calculated from a linear model based on the log-transformed posterior medians and the observed point estimates. Results for the simpler geometric mean (*n* = ½) model are similar, with residual SE of 1.33-fold, 95% CrI range of 3.01-fold, and adjusted R^2^ = 0.78. **Fig C.** Scatter plot of seroprevalence predictions (posterior median for primary random effects model) versus validation data (reported point estimate and 95% CI). The solid line represents equality, the dashed line is +/- one residual standard error, and the dotted line is the 95% CrI residual error. The adjusted R^2^ is calculated from a linear model based on the log-transformed posterior medians and the observed point estimates. Results for the simpler geometric mean (*n* = ½) model are similar, with residual SE of 1.39-fold, 95% CrI range of 3.62-fold, and adjusted R^2^ = 0.77. **Fig D.** Scatter plot of active infection prevalence predictions from semi-empirical model (posterior median for primary random effects model) versus those from epidemiologic models (posterior median and 95% CrI). The solid line represents equality. The residual standard error (RSE) and adjusted R^2^ are from the comparison of natural log-transformed median predictions. Results for the simpler geometric mean (*n* = ½) model are similar, with RSEs of 1.71-fold and 2.01-fold, 95% CrI ranges of 1.77-fold and 2.01-fold, and adjusted R^2^ values = 0.73 and 0.71, for the Extended SEIR and Imperial models, respectively. **Fig E.** Boxplots (box = IQR, line = median, whiskers = 95% CrI) of posterior estimate of infection prevalence (A) and seroprevalence (B) across states and for the U.S. overall as of December 31, 2020, using the primary random effects model. In (B), for comparison, cumulative reported cases are shown with a 14-day lag to allow time for seroconversion (error bars denote range of 7–21 day lags). **Fig F.** A) Map of estimated undiagnosed (A) and total (B) prevalence and transmission trends and overall seroprevalence (C) as of December 31, 2020, based on data through January 15, 2021. Values based on the simpler geometric mean model (see Fig 4 for primary random effects model predictions). The maps were generated using the R package usmap https://cran.r-project.org/web/packages/usmap/index.html (GPL-3), which uses shape files from the U.S. Census Bureau (the link provided in documentation is here: https://www.census.gov/geographies/mapping-files/time-series/geo/tiger-line-file.html). **Fig G.** Boxplots (box = IQR, line = median, whiskers = 95% CrI) of posterior estimate of infection prevalence (A) and seroprevalence (B) across states and for the U.S. overall as of December 31, 2020, using the simpler geometric mean model. In (B), for comparison, cumulative reported cases are shown with a 14-day lag to allow time for seroconversion (error bars denote range of 7–21 day lags). **Fig H.** Bias estimates from primary random effects model. A, B) Comparison of test positivity (14-day average) and semi-empirical prevalence estimates (median and 95% CrI) across all states (A) or across the U.S. in aggregate (B) from April 1-December 31, 2020. Diagonal lines denote different levels of positivity bias, as illustrated in **Fig 1A**. C, D) Comparison of cumulative reported cases, with 14-day lag to allow for conversion to seropositivity, and semi-empirical seropositivity estimates (median and 95% CrI) across all states (C) or across the U.S. in aggregate (D) from April 1-December 31, 2020. Diagonal lines denote different levels of cumulative case under-reporting. Results for the simpler geometric mean (*n* = ½) model are similar. **Fig I.** Examples of five states where the trends in reported case rates and positivity rates diverged (i.e., one increasing, the other decreasing). For each state, the top panel is the active infection (total diagnosed and undiagnosed) prevalence as predicted by the semi-empirical model (posterior median and 95% CrI), the second panel is the active undiagnosed infection prevalence, whereas the bottom three panels show the reported case, positivity, and testing rates, each averaged over the previous 14 days. **Fig J.** Application of semi-empirical model using random effects posterior distributions from U.S. states to other nations/countries. COVID-19 antibody seroprevalence estimates (posterior median and 95% credible intervals) for each nation/country with state-wide seroprevalence data (**Table F**, reported point estimates and 95% confidence intervals shown). **Fig K.** Conceptual model of undiagnosed prevalence (Eqs 7–9). Assuming a time interval between infection and seropositivity = *T*_inf_, each time point *t*, we can subdivide the undiagnosed infection prevalence *I*_*U*_ into *T*_inf_ “subcompartments” *I*_*U*,*m*_ (*m* = 1…*T*_inf_). The number of undiagnosed individuals who are diagnosed each day is *I*_*U*_ ✕ Λ (diagnosis considered sampling without replacement of *I*_*U*_). The number of undiagnosed individuals who become newly undiagnosed seropositive (entering SP_U_ the next day) is simply the number in the last subcompartment $I_{U,T_{inf}}$ multiplied by another factor of (1 –Λ) to account for the fraction that get diagnosed that day. **Fig L. Sensitivity of parameter estimates to changing averaging time τ from 14 to 7 or 28 days.** A) Posterior distributions of power parameter *n*; B) posterior distributions of seroprevalence offset SP_o_. **Fig M. Sensitivity of seroprevalence predictions to changing averaging time τ from 14 to 7 or 28 days.** All predictions are posterior medians. **Fig N. Sensitivity of undiagnosed prevalence predictions to changing averaging time τ from 14 to 7 or 28 days.** All predictions are posterior medians. **Fig O. Sensitivity of total prevalence predictions to changing averaging time τ from 14 to 7 or 28 days.** All predictions are posterior medians.(DOCX)
