# Supplementary material for: Using test positivity and reported case rates to estimate state-level COVID-19 prevalence and seroprevalence in the United States
Source: PLoS Comput Biol. 2021 Sep 7;17(9):e1009374. doi: 10.1371/journal.pcbi.1009374 (PMC8448371; doi:10.1371/journal.pcbi.1009374)
Supplement: S1 Text — Table A. State-wide seroprevalence calibration data. Table B. State-wide seroprevalence validation data. Table C. Posterior distributions and convergence diagnostic of n and SPo for individual states (random effects). Table D. Primary model posterior estimates of prevalence (undiagnosed and total) and seroprevalence as of December 31, 2020. Table E. Geometric mean model posterior estimates of prevalence (undiagnosed and total) and seroprevalence as of December 31, 2020. Table F. International seroprevalence data. Fig A. Posterior distributions of the power parameter n and the seroprevalence offset SPo for individual states using the primary random effects model. The fixed effect is denoted by “F.E.,” and the vertical dashed line represents its posterior median. For the simpler geometric mean model, the power parameter is fixed at n = ½, and the F.E. posterior median [CrI] for SPo is 0.90 [0.38–1.50]. Fig B. Scatter plot of seroprevalence predictions (posterior median for primary random effects model) versus calibration data (reported point estimate and 95% CI). The solid line represents equality, the dashed line is +/- one residual standard error, and the dotted line is the 95% CrI residual error. The adjusted R2 is calculated from a linear model based on the log-transformed posterior medians and the observed point estimates. Results for the simpler geometric mean (n = ½) model are similar, with residual SE of 1.33-fold, 95% CrI range of 3.01-fold, and adjusted R2 = 0.78. Fig C. Scatter plot of seroprevalence predictions (posterior median for primary random effects model) versus validation data (reported point estimate and 95% CI). The solid line represents equality, the dashed line is +/- one residual standard error, and the dotted line is the 95% CrI residual error. The adjusted R2 is calculated from a linear model based on the log-transformed posterior medians and the observed point estimates. Results for the simpler geometric mean (n = ½) model are similar, with [file pcbi.1009374.s001.docx]

**S1 Text: Using Test Positivity and Reported Case Rates to Estimate State-Level
COVID-19 Prevalence and Seroprevalence in the United States**

Weihsueh A. Chiu^1^, Martial L. Ndeffo-Mbah^1,2^

^1^Department of Veterinary Integrative Biosciences, College of Veterinary Medicine and Biomedical Sciences, Texas A&M University, College Station, Texas, USA

^2^Department of Epidemiology and Biostatistics, School of Public Health, Texas A&M University, College Station, Texas, USA

**Supplemental Methods**

***Bayesian calibration and validation to seroprevalence data***

To calibrate and validate the model, we utilized state-wide seroprevalence data, which has only recently become available for all 50 states and the District of Columbia (**Table A**). We only included data from states where the entire state was sampled or where adjustments for convenience sampling bias were integrated into the analysis (1–6), and verified our selections by comparison to three independent systematic reviews (7–9). Data were extracted from study tables or supplemental materials. Additional data were downloaded from the CDC Initial Ten-Site Commercial Laboratory Seroprevalence Survey for 4 states (CT, LA, MO, UT), and the Nationwide Commercial Laboratory Seroprevalence Survey for all 50 states and DC (10). For this last dataset, Rounds 1-4 were reported previously by Bajema et al. (5), Rounds 5-7 were included in calibration, and Rounds 8-11 were used for validation.

We used a Bayesian approach to estimate model parameters, using the prior distributions shown in **Table 1**. Because states differ somewhat in their testing availability and capacity, as well as in the extent to which the initial surge of infections was missed, we assume random effects for the power parameter *n* and the initial condition for seroprevalence SP_o_. We use a logit transformation to restrict values between 0 and 1, where SP_o_ is scaled by state population, and assume state-to-state variation on the transformed values is distributed normally with unknown random effects standard deviation Σ. We use non-informative prior distributions consisting of a uniform distribution on the logit scale for the fixed effects μ, and a log-uniform distribution for Σ. For the duration of infection *T*_inf_, we assign an informative prior distribution with mean 14 days and standard deviation 3.5 days, corresponding to a 95% CI range of 7-21 days, based on the CDC estimate that it takes 7-21 days after infection to become seropositive (11).

The likelihood function assumes independent log-normal distributed errors given an observed seroprevalence $y_{obs}$ and estimated seroprevalence (equation 6) $y_{pred}$:

|  | $ln y_{obs} =ln y_{pred}+\epsilon$  $\epsilon\sim N(0,\sigma_{obs}^{2}+\sigma_{err}^{2})$ | (7) |
| --- | --- | --- |

with the observed error variance $\sigma_{obs}^{2}$ estimated from reported 95% CI for each observation $[\sigma_{obs}=(ln {UCL}_{obs}-ln y_{obs} )/z_{97.5}]$, where ${UCL}_{obs}$ is the reported upper confidence limit and $z_{97.5}$ is the z-score of the 97.5^th^ percentile (in some cases, the reported lower confidence limit was 0, so only the upper confidence limit was used to calculate $\sigma_{obs}^{2}$). The residual error standard deviation $\sigma_{err}$ is assigned a log-uniform prior.

Markov chain Monte Carlo (MCMC) simulation was conducted to sample from the posterior distribution. Sampling was conducted using component-wise Metropolis sampling with a normal proposal distribution (12). The variance of the proposal distribution was adjusted for each parameter to maintain an acceptance rate between 20% and 60%. Four independent chains with different starting points and random seeds were run, and the potential scale reduction factor (PSRF) was used to assess convergence, with a value of <1.2 regarded as adequate (13,14).

***Data***

U.S. testing and reported case data were downloaded from the COVID Tracking Project using their API (15). In the data definitions, the fields “positiveIncrease” (confirmed plus probable [if reported] cases of COVID-19 reported) and “totalTestResultsIncrease” (increase in number of test results, computed differently depending on state) were used to represent daily reported cases and total test results, respectively. International testing and case data were downloaded from Our World in Data (16) or the U.K. government (17).

**References**

1. Havers FP, Reed C, Lim T, Montgomery JM, Klena JD, Hall AJ, et al. Seroprevalence of Antibodies to SARS-CoV-2 in 10 Sites in the United States, March 23-May 12, 2020. JAMA Intern Med [Internet]. 2020 [cited 2020 Aug 28]; Available from: https://jamanetwork.com/

2. Menachemi N, Yiannoutsos CT, Dixon BE, Duszynski TJ, Fadel WF, Wools-Kaloustian KK, et al. Population Point Prevalence of SARS-CoV-2 Infection Based on a Statewide Random Sample — Indiana, April 25–29, 2020. MMWR Morb Mortal Wkly Rep [Internet]. 2020 Jul 24 [cited 2020 Aug 28];69(29):960–4. Available from: http://www.cdc.gov/mmwr/volumes/69/wr/mm6929e1.htm?s_cid=mm6929e1_w

3. Rosenberg ES, Tesoriero JM, Rosenthal EM, Chung R, Barranco MA, Styer LM, et al. Cumulative incidence and diagnosis of SARS-CoV-2 infection in New York. Ann Epidemiol. 2020 Aug 1;48:23-29.e4.

4. Anand S, Montez-Rath M, Han J, Bozeman J, Kerschmann R, Beyer P, et al. Prevalence of SARS-CoV-2 antibodies in a large nationwide sample of patients on dialysis in the USA: a cross-sectional study. Lancet [Internet]. 2020 Oct 24 [cited 2020 Dec 14];396(10259):1335–44. Available from: https://doi.org/10.1016/

5. Bajema KL, Wiegand RE, Cuffe K, Patel S V., Iachan R, Lim T, et al. Estimated SARS-CoV-2 Seroprevalence in the US as of September 2020. JAMA Intern Med [Internet]. 2020 [cited 2020 Dec 14]; Available from: https://jamanetwork.com/

6. CDC COVID Data Tracker [Internet]. [cited 2020 Aug 28]. Available from: https://covid.cdc.gov/covid-data-tracker/?CDC_AA_refVal=https%3A%2F%2Fwww.cdc.gov%2Fcoronavirus%2F2019-ncov%2Fcases-updates%2Fcommercial-labs-interactive-serology-dashboard.html#serology-surveillance

7. Rostami A, Sepidarkish M, Leeflang MMG, Riahi SM, Nourollahpour Shiadeh M, Esfandyari S, et al. SARS-CoV-2 seroprevalence worldwide: a systematic review and meta-analysis [Internet]. Vol. 0, Clinical Microbiology and Infection. Elsevier B.V.; 2020 [cited 2020 Dec 14]. Available from: https://doi.org/10.1016/j.cmi.2020.10.020

8. Franceschi VB, Santos AS, Glaeser AB, Paiz JC, Caldana GD, Machado Lessa CL, et al. Population‐based prevalence surveys during the Covid‐19 pandemic: A systematic review. Rev Med Virol [Internet]. 2020 Dec 4 [cited 2020 Dec 14]; Available from: https://onlinelibrary.wiley.com/doi/10.1002/rmv.2200

9. Bobrovitz N, Krishan Arora R, Cao C, Boucher E, Liu M, Rahim H, et al. Global seroprevalence of SARS-CoV-2 antibodies: a systematic review and meta-analysis. medRxiv [Internet]. 2020 Nov 18 [cited 2020 Dec 14];2020.11.17.20233460. Available from: https://doi.org/10.1101/2020.11.17.20233460

10. Commercial Laboratory Seroprevalence Surveys | Coronavirus | COVID-19 | CDC [Internet]. [cited 2020 Aug 28]. Available from: https://www.cdc.gov/coronavirus/2019-ncov/cases-updates/commercial-lab-surveys.html#interpreting-serology-results

11. Serology Testing for COVID-19 at CDC | CDC [Internet]. [cited 2020 Dec 14]. Available from: https://www.cdc.gov/coronavirus/2019-ncov/lab/serology-testing.html

12. Hastings WK. Monte Carlo sampling methods using Markov chains and their applications. Biometrika [Internet]. 1970 Apr 1 [cited 2020 Dec 14];57(1):97–109. Available from: https://academic.oup.com/biomet/article/57/1/97/284580

13. Gelman A, Rubin DB. Inference from Iterative Simulation Using Multiple Sequences [Internet]. Vol. 7, Statistical Science. Institute of Mathematical Statistics; [cited 2020 Jul 4]. p. 457–72. Available from: https://www.jstor.org/stable/2246093

14. Brooks S, Gelman A. General Methods for Monitoring Convergence of Iterative Simulations. J Am Stat Assoc [Internet]. 1998 [cited 2013 Jan 8];7(7):434–55. Available from: http://www.citeulike.org/user/fonnesbeck/article/1176225

15. The COVID Tracking Project | The COVID Tracking Project [Internet]. [cited 2020 May 18]. Available from: https://covidtracking.com/

16. Our World in Data [Internet]. [cited 2020 Dec 14]. Available from: https://ourworldindata.org/

17. Daily summary | Coronavirus in the UK [Internet]. [cited 2020 Dec 14]. Available from: https://coronavirus.data.gov.uk/

18. Herzog S, De Bie J, Abrams S, Wouters I, Ekinci E, Patteet L, et al. Seroprevalence of IgG antibodies against SARS coronavirus 2 in Belgium – a serial prospective cross-sectional nationwide study of residual samples. medRxiv [Internet]. 2020 Oct 1 [cited 2020 Dec 15];2020.06.08.20125179. Available from: https://doi.org/10.1101/2020.06.08.20125179

19. (No Title) [Internet]. [cited 2020 Dec 15]. Available from: https://www.covid19immunitytaskforce.ca/wp-content/uploads/2020/09/COVID-19-Public-Report_2.pdf

20. Erikstrup C, Hother CE, Pedersen OBV, Mølbak K, Skov RL, Holm DK, et al. Estimation of SARS-CoV-2 Infection Fatality Rate by Real-time Antibody Screening of Blood Donors. Clin Infect Dis [Internet]. 2020 Jun 25 [cited 2020 Dec 15]; Available from: https://academic.oup.com/cid/advance-article/doi/10.1093/cid/ciaa849/5862661

21. (No Title) [Internet]. [cited 2020 Dec 15]. Available from: https://assets.publishing.service.gov.uk/government/uploads/system/uploads/attachment_data/file/911002/Weekly_COVID19_Surveillance_Report_week_34_FINAL.pdf

22. (No Title) [Internet]. [cited 2020 Dec 15]. Available from: https://assets.publishing.service.gov.uk/government/uploads/system/uploads/attachment_data/file/907954/Weekly_COVID19_Surveillance_Report_week_32_2.pdf

23. Coronavirus (COVID-19) Infection Survey pilot - Office for National Statistics [Internet]. [cited 2020 Dec 15]. Available from: https://www.ons.gov.uk/peoplepopulationandcommunity/healthandsocialcare/conditionsanddiseases/bulletins/coronaviruscovid19infectionsurveypilot/englandandwales14august2020

24. Coronavirus (COVID-19) Infection Survey pilot - Office for National Statistics [Internet]. [cited 2020 Dec 15]. Available from: https://www.ons.gov.uk/peoplepopulationandcommunity/healthandsocialcare/conditionsanddiseases/bulletins/coronaviruscovid19infectionsurveypilot/england17july2020

25. (No Title) [Internet]. [cited 2020 Dec 15]. Available from: https://assets.publishing.service.gov.uk/government/uploads/system/uploads/attachment_data/file/912973/Weekly_COVID19_Surveillance_Report_week_35_FINAL.PDF

26. Ward H, Atchison CJ, Whitaker M, Ainslie KEC, Elliott J, Okell LC, et al. Antibody prevalence for SARS-CoV-2 in England following first peak of the pandemic: REACT2 study in 100,000 adults. medRxiv [Internet]. 2020 Aug 21 [cited 2020 Dec 15];2020.08.12.20173690. Available from: https://doi.org/10.1101/2020.08.12.20173690

27. Bogogiannidou Z, Vontas A, Dadouli K, Kyritsi MA, Soteriades S, Nikoulis DJ, et al. Repeated leftover serosurvey of SARS-CoV-2 IgG antibodies, Greece, March and April 2020. Eurosurveillance [Internet]. 2020 Aug 6 [cited 2020 Dec 15];25(31):1–6. Available from: https://www.eurosurveillance.org/content/10.2807/1560-7917.ES.2020.25.31.2001369

28. Merkely B, Szabó AJ, Kosztin A, Berényi E, Sebestyén A, Lengyel C, et al. Novel coronavirus epidemic in the Hungarian population, a cross-sectional nationwide survey to support the exit policy in Hungary. GeroScience [Internet]. 2020 Aug 1 [cited 2020 Dec 15];42(4):1063–74. Available from: https://doi.org/10.1007/s11357-020-00226-9

29. Gudbjartsson DF, Helgason A, Jonsson H, Magnusson OT, Melsted P, Norddahl GL, et al. Spread of SARS-CoV-2 in the Icelandic Population. N Engl J Med [Internet]. 2020 Jun 11 [cited 2020 Dec 15];382(24):2302–15. Available from: https://www.nejm.org/doi/10.1056/NEJMoa2006100

30. (No Title) [Internet]. [cited 2020 Dec 15]. Available from: https://www.hpsc.ie/a-z/respiratory/coronavirus/novelcoronavirus/scopi/SCOPI report preliminary results final version.pdf

31. Snoeck C, Vaillant M, Abdelrahman T, Satagopam V, Turner J, Beaumont K, et al. Prevalence of SARS-CoV-2 infection in the Luxembourgish population: the CON-VINCE study. medRxiv [Internet]. 2020 May 18 [cited 2020 Dec 15];2020.05.11.20092916. Available from: https://doi.org/10.1101/2020.05.11.20092916

32. Seroprevalence of SARS-CoV-2 in the Norwegian population measured in residual sera collected in April/May 2020 and August 2019 - NIPH [Internet]. [cited 2020 Dec 15]. Available from: https://www.fhi.no/en/publ/2020/seroprevalence-of-sars-cov-2-in-the-norwegian-population--measured-in-resid/

33. INSA (Instituto National de Saude)- Doutor Ricardo Jorge [Internet]. [cited 2020 Dec 15]. Available from: http://www.insa.min-saude.pt/resultadospreliminares-do-primeiro-inquerito-serologico-nacional-covid-19-relatorio/

34. 1 in 7 Russians Have ‘Coronavirus Immunity’ – Official - The Moscow Times [Internet]. [cited 2020 Dec 15]. Available from: https://www.themoscowtimes.com/2020/06/10/1-in-7-russians-have-coronavirus-immunity-official-a70540

35. Maver Vodičar P, Oštrbenk Valenčak A, Zupan B, Avšič Županc T, Kurdija S, Korva M, et al. Low prevalence of active COVID-19 in Slovenia: a nationwide population study of a probability-based sample. Clin Microbiol Infect. 2020 Nov 1;26(11):1514–9.

36. Nationwide Commercial Laboratory Seroprevalence Survey | Data | Centers for Disease Control and Prevention. [cited 19 Jul 2021]. Available: https://data.cdc.gov/Laboratory-Surveillance/Nationwide-Commercial-Laboratory-Seroprevalence-Su/d2tw-32xv

**Tables and Figures legends:**

**Table A.** State-wide seroprevalence calibration data

**Table B.** State-wide seroprevalence validation data

**Table C:** Posterior distributions and convergence diagnostic of *n* and SP_o_ for individual states (random effects)

**Table D.** Primary model posterior estimates of prevalence (undiagnosed and total) and seroprevalence as of December 31, 2020.

**Table E.** Geometric mean model posterior estimates of prevalence (undiagnosed and total) and seroprevalence as of December 31, 2020.

**Table F.** International seroprevalence data.

**Fig A:** Posterior distributions of the power parameter n and the seroprevalence offset SP_o_ for individual states using the primary random effects model. The fixed effect is denoted by “F.E.,” and the vertical dashed line represents its posterior median. For the simpler geometric mean model, the power parameter is fixed at *n*=½, and the F.E. posterior median [CrI] for SP_o_ is 0.90 [0.38-1.50].

**Fig B:** Scatter plot of seroprevalence predictions (posterior median for primary random effects model) versus calibration data (reported point estimate and 95% CI). The solid line represents equality, the dashed line is +/- one residual standard error, and the dotted line is the 95% CrI residual error. The adjusted R^2^ is calculated from a linear model based on the log-transformed posterior medians and the observed point estimates. Results for the simpler geometric mean (*n*=½) model are similar, with residual SE of 1.33-fold, 95% CrI range of 3.01-fold, and adjusted R^2^ = 0.78.

**Fig C:** Scatter plot of seroprevalence predictions (posterior median for primary random effects model) versus validation data (reported point estimate and 95% CI). The solid line represents equality, the dashed line is +/- one residual standard error, and the dotted line is the 95% CrI residual error. The adjusted R^2^ is calculated from a linear model based on the log-transformed posterior medians and the observed point estimates. Results for the simpler geometric mean (*n*=½) model are similar, with residual SE of 1.39-fold, 95% CrI range of 3.62-fold, and adjusted R^2^ = 0.77.

**Fig D:** Scatter plot of active infection prevalence predictions from semi-empirical model (posterior median for primary random effects model) versus those from epidemiologic models (posterior median and 95% CrI). The solid line represents equality. The residual standard error (RSE) and adjusted R^2^ are from the comparison of natural log-transformed median predictions. Results for the simpler geometric mean (*n*=½) model are similar, with RSEs of 1.71-fold and 2.01-fold, 95% CrI ranges of 1.77-fold and 2.01-fold, and adjusted R^2^ values = 0.73 and 0.71, for the Extended SEIR and Imperial models, respectively.

**Fig E:** Boxplots (box=IQR, line=median, whiskers=95% CrI) of posterior estimate of infection prevalence (A) and seroprevalence (B) across states and for the U.S. overall as of December 31, 2020, using the primary random effects model. In (B), for comparison, cumulative reported cases are shown with a 14-day lag to allow time for seroconversion (error bars denote range of 7-21 day lags).

**Fig F.** A) Map of estimated undiagnosed (A) and total (B) prevalence and transmission trends and overall seroprevalence (C) as of December 31, 2020, based on data through January 15, 2021. Values based on the simpler geometric mean model (see Figure 4 for primary random effects model predictions). The maps were generated using the R package usmap <https://cran.r-project.org/web/packages/usmap/index.html> (GPL-3), which uses shape files from the U.S. Census Bureau (the link provided in documentation is here: <https://www.census.gov/geographies/mapping-files/time-series/geo/tiger-line-file.html>).

**Fig G:** Boxplots (box=IQR, line=median, whiskers=95% CrI) of posterior estimate of infection prevalence (A) and seroprevalence (B) across states and for the U.S. overall as of December 31, 2020, using the simpler geometric mean model. In (B), for comparison, cumulative reported cases are shown with a 14-day lag to allow time for seroconversion (error bars denote range of 7-21 day lags).

**Fig H.** Bias estimates from primary random effects model. A, B) Comparison of test positivity (14-day average) and semi-empirical prevalence estimates (median and 95% CrI) across all states (A) or across the U.S. in aggregate (B) from April 1-December 31, 2020. Diagonal lines denote different levels of positivity bias, as illustrated in Figure 1A. C, D) Comparison of cumulative reported cases, with 14-day lag to allow for conversion to seropositivity, and semi-empirical seropositivity estimates (median and 95% CrI) across all states (C) or across the U.S. in aggregate (D) from April 1-December 31, 2020. Diagonal lines denote different levels of cumulative case under-reporting. Results for the simpler geometric mean (*n*=½) model are similar.

**Fig I:** Examples of five states where the trends in reported case rates and positivity rates diverged (i.e., one increasing, the other decreasing). For each state, the top panel is the active infection (total diagnosed and undiagnosed) prevalence as predicted by the semi-empirical model (posterior median and 95% CrI), the second panel is the active undiagnosed infection prevalence, whereas the bottom three panels show the reported case, positivity, and testing rates, each averaged over the previous 14 days.

**Fig J:** Application of semi-empirical model using random effects posterior distributions from U.S. states to other nations/countries. COVID-19 antibody seroprevalence estimates (posterior median and 95% credible intervals) for each nation/country with state-wide seroprevalence data (**Table F**, reported point estimates and 95% confidence intervals shown).

**Fig K:** Conceptual model of undiagnosed prevalence (equations 7-9). Assuming a time interval between infection and seropositivity = *T*_inf_, each time point *t*, we can subdivide the undiagnosed infection prevalence *I_U_* into *T*_inf_ “subcompartments” *I_U,m_* (*m* = 1...*T*_inf_). The number of undiagnosed individuals who are diagnosed each day is *I_U_* ✕ Λ (diagnosis considered sampling without replacement of *I_U_*). The number of undiagnosed individuals who become newly undiagnosed seropositive (entering SP_U_ the next day) is simply the number in the last subcompartment $I_{U,T_{inf}}$multiplied by another factor of (1 – Λ) to account for the fraction that get diagnosed that day.

**Fig L: Sensitivity of parameter estimates to changing averaging time τ from 14 to 7 or 28 days.** A) Posterior distributions of power parameter *n*; B) posterior distributions of seroprevalence offset SP_o_.

**Fig M: Sensitivity of seroprevalence predictions to changing averaging time τ from 14 to 7 or 28 days.** All predictions are posterior medians.

**Fig N: Sensitivity of undiagnosed prevalence predictions to changing averaging time τ from 14 to 7 or 28 days.** All predictions are posterior medians.

**Fig O: Sensitivity of total prevalence predictions to changing averaging time τ from 14 to 7 or 28 days.** All predictions are posterior medians.

**Tables and Figures:**

**Table A.** State-wide seroprevalence calibration data

| **State** | **Min date** | **Max date** | **Point est. (%)** | **LCL (%)** | **UCL (%)** | **Source** |
| --- | --- | --- | --- | --- | --- | --- |
| NY | 3/29/20 | 3/29/20 | 14 | 13.3 | 14.7 | (3) |
| IN | 4/25/20 | 4/29/20 | 2.79 | 2.02 | 3.7 | (2) |
| CT | 4/26/20 | 5/3/20 | 4.9 | 3.6 | 6.5 | (1,6) |
| CT | 5/21/20 | 5/26/20 | 5.2 | 3.9 | 6.6 | (1,6) |
| CT | 6/15/20 | 6/17/20 | 6.3 | 5 | 7.8 | (1,6) |
| CT | 7/3/20 | 7/6/20 | 5.2 | 4 | 6.7 | (1,6) |
| LA | 4/1/20 | 4/8/20 | 5.8 | 3.9 | 8.2 | (1,6) |
| MN | 4/30/20 | 5/12/20 | 2.4 | 1 | 4.5 | (1,6) |
| MN | 5/25/20 | 6/7/20 | 2.2 | 1.4 | 3.3 | (1,6) |
| MN | 6/15/20 | 6/27/20 | 4.3 | 3.4 | 5.8 | (1,6) |
| MN | 7/6/20 | 7/17/20 | 6.1 | 4.4 | 8.3 | (1,6) |
| MO | 4/20/20 | 4/26/20 | 2.7 | 1.7 | 3.9 | (1,6) |
| MO | 5/25/20 | 5/30/20 | 2.8 | 1.7 | 4.1 | (1,6) |
| MO | 6/15/20 | 6/20/20 | 0.8 | 0.6 | 1.8 | (1,6) |
| MO | 7/5/20 | 7/9/20 | 1.4 | 0.9 | 2.5 | (1,6) |
| UT | 4/20/20 | 5/3/20 | 2.2 | 1.2 | 3.4 | (1,6) |
| UT | 5/25/20 | 6/5/20 | 1.1 | 0.6 | 2.1 | (1,6) |
| UT | 6/15/20 | 6/27/20 | 1.5 | 0.9 | 2.6 | (1,6) |
| UT | 7/6/20 | 7/15/20 | 2.7 | 1.8 | 3.9 | (1,6) |
| AK | 7/1/20 | 7/31/20 | 0.7 | 0.1 | 4.7 | (4) |
| AL | 7/1/20 | 7/31/20 | 4.2 | 1.8 | 10.2 | (4) |
| AR | 7/1/20 | 7/31/20 | 1.9 | 1 | 3.5 | (4) |
| AZ | 7/1/20 | 7/31/20 | 4.7 | 2.5 | 9.1 | (4) |
| CA | 7/1/20 | 7/31/20 | 3.8 | 3.3 | 4.3 | (4) |
| CO | 7/1/20 | 7/31/20 | 1.9 | 0.3 | 13.4 | (4) |
| CT | 7/1/20 | 7/31/20 | 8.5 | 4.6 | 15.9 | (4) |
| DC | 7/1/20 | 7/31/20 | 21.3 | 11.4 | 39.5 | (4) |
| DE | 7/1/20 | 7/31/20 | 5.7 | 2.4 | 13.8 | (4) |
| FL | 7/1/20 | 7/31/20 | 4.6 | 3.6 | 5.8 | (4) |
| GA | 7/1/20 | 7/31/20 | 4.5 | 3.5 | 5.9 | (4) |
| HI | 7/1/20 | 7/31/20 | 0.1 | 0 | 0.9 | (4) |
| IA | 7/1/20 | 7/31/20 | 2.6 | 1.1 | 6.2 | (4) |
| IL | 7/1/20 | 7/31/20 | 17.5 | 15.2 | 20.2 | (4) |
| IN | 7/1/20 | 7/31/20 | 3.1 | 1.8 | 5.5 | (4) |
| KY | 7/1/20 | 7/31/20 | 4.8 | 1.5 | 14.8 | (4) |
| LA | 7/1/20 | 7/31/20 | 17.6 | 10.8 | 28.7 | (4) |
| MA | 7/1/20 | 7/31/20 | 11.3 | 7 | 18.2 | (4) |
| MD | 7/1/20 | 7/31/20 | 12.9 | 10 | 16.7 | (4) |
| MI | 7/1/20 | 7/31/20 | 7.2 | 3.2 | 16.1 | (4) |
| MN | 7/1/20 | 7/31/20 | 6.3 | 0.9 | 44.4 | (4) |
| MO | 7/1/20 | 7/31/20 | 1.9 | 0.9 | 3.8 | (4) |
| MS | 7/1/20 | 7/31/20 | 4.5 | 1.9 | 10.8 | (4) |
| NC | 7/1/20 | 7/31/20 | 1.9 | 0.5 | 7.7 | (4) |
| NE | 7/1/20 | 7/31/20 | 2.5 | 0.9 | 6.5 | (4) |
| NJ | 7/1/20 | 7/31/20 | 11.9 | 8.5 | 16.6 | (4) |
| NM | 7/1/20 | 7/31/20 | 5.7 | 3.4 | 9.7 | (4) |
| NY | 7/1/20 | 7/31/20 | 33.6 | 31.7 | 35.6 | (4) |
| OH | 7/1/20 | 7/31/20 | 3 | 2.2 | 4.1 | (4) |
| OK | 7/1/20 | 7/31/20 | 0.5 | 0.1 | 3.8 | (4) |
| OR | 7/1/20 | 7/31/20 | 4.8 | 1.8 | 12.7 | (4) |
| PA | 7/1/20 | 7/31/20 | 6.4 | 4.7 | 8.8 | (4) |
| SC | 7/1/20 | 7/31/20 | 2.2 | 1.1 | 4.4 | (4) |
| TN | 7/1/20 | 7/31/20 | 2.1 | 1.3 | 3.6 | (4) |
| TX | 7/1/20 | 7/31/20 | 3.6 | 3.1 | 4.2 | (4) |
| UT | 7/1/20 | 7/31/20 | 3.1 | 1.8 | 5.1 | (4) |
| VA | 7/1/20 | 7/31/20 | 9.1 | 6.3 | 13.1 | (4) |
| WA | 7/1/20 | 7/31/20 | 5.1 | 3.8 | 6.8 | (4) |
| WI | 7/1/20 | 7/31/20 | 5 | 1.3 | 20 | (4) |
| AK | 8/6/20 | 8/11/20 | 0.3 | 0 | 1.12 | (5) |
| AL | 7/29/20 | 8/13/20 | 5.8 | 4.16 | 7.71 | (5) |
| AR | 7/29/20 | 8/13/20 | 4.1 | 2.74 | 5.63 | (5) |
| AZ | 7/31/20 | 8/11/20 | 8.2 | 4.11 | 13.59 | (5) |
| CA | 7/30/20 | 8/5/20 | 5.7 | 4.05 | 7.77 | (5) |
| CO | 7/30/20 | 8/7/20 | 2.4 | 0.89 | 4.53 | (5) |
| CT | 7/30/20 | 8/3/20 | 3.4 | 2.04 | 4.72 | (5) |
| DC | 7/30/20 | 8/13/20 | 3.9 | 2.18 | 5.76 | (5) |
| DE | 7/29/20 | 8/13/20 | 5.7 | 3.55 | 8.22 | (5) |
| FL | 7/31/20 | 8/3/20 | 4.3 | 2.77 | 5.86 | (5) |
| GA | 8/2/20 | 8/11/20 | 6.8 | 4.83 | 8.81 | (5) |
| HI | 8/3/20 | 8/11/20 |  |  |  | (5) |
| IA | 7/29/20 | 8/13/20 | 8.6 | 5.98 | 11.34 | (5) |
| ID | 8/4/20 | 8/11/20 | 4.5 | 1.8 | 7.99 | (5) |
| IL | 7/29/20 | 8/10/20 | 3.9 | 2.52 | 5.21 | (5) |
| IN | 7/31/20 | 8/11/20 | 2.2 | 1.1 | 3.63 | (5) |
| KS | 7/29/20 | 8/8/20 | 1.6 | 0.7 | 2.8 | (5) |
| KY | 7/30/20 | 8/13/20 | 2.4 | 1.32 | 3.62 | (5) |
| LA | 7/28/20 | 8/13/20 | 9.6 | 6.88 | 12.29 | (5) |
| MA | 7/30/20 | 8/10/20 | 4.2 | 2.89 | 5.81 | (5) |
| MD | 7/31/20 | 8/11/20 | 9.7 | 7.26 | 12.87 | (5) |
| ME | 7/30/20 | 8/11/20 | 0.5 | 0 | 1.16 | (5) |
| MI | 7/30/20 | 8/11/20 | 3.4 | 2.16 | 4.84 | (5) |
| MN | 7/29/20 | 8/13/20 | 3.5 | 2.06 | 5.01 | (5) |
| MO | 7/28/20 | 8/10/20 | 2.5 | 1.44 | 3.61 | (5) |
| MS | 7/30/20 | 8/13/20 | 7.1 | 3.75 | 11.09 | (5) |
| MT | 7/29/20 | 8/10/20 | 0.5 | 0 | 1.67 | (5) |
| NC | 7/29/20 | 8/10/20 | 2.5 | 1.31 | 3.72 | (5) |
| ND | 7/29/20 | 8/12/20 | 7.3 | 1.3 | 14.28 | (5) |
| NE | 7/28/20 | 8/13/20 | 7.4 | 5.25 | 9.75 | (5) |
| NH | 7/30/20 | 8/11/20 | 0.8 | 0.22 | 1.48 | (5) |
| NJ | 7/31/20 | 8/11/20 | 14.8 | 12.23 | 17.57 | (5) |
| NM | 7/29/20 | 8/13/20 | 2 | 1.09 | 3.02 | (5) |
| NV | 7/30/20 | 8/2/20 | 5.1 | 3.63 | 6.66 | (5) |
| NY | 7/31/20 | 8/11/20 | 23.3 | 20.07 | 26.32 | (5) |
| OH | 7/29/20 | 8/11/20 | 2.3 | 1.11 | 3.61 | (5) |
| OK | 7/28/20 | 8/4/20 | 1.6 | 0.85 | 2.49 | (5) |
| OR | 8/5/20 | 8/11/20 | 2.3 | 1.03 | 3.84 | (5) |
| PA | 7/31/20 | 8/11/20 | 10.2 | 5.71 | 17.21 | (5) |
| PR | 7/27/20 | 8/7/20 | 1.1 | 0.43 | 1.8 | (5) |
| RI | 7/30/20 | 8/11/20 | 3 | 1.23 | 5.51 | (5) |
| SC | 7/30/20 | 8/13/20 | 8.1 | 5.79 | 10.69 | (5) |
| SD | 7/29/20 | 8/12/20 |  |  |  | (5) |
| TN | 7/30/20 | 8/11/20 | 6.3 | 4.41 | 8.25 | (5) |
| TX | 7/29/20 | 8/5/20 | 5.9 | 4.04 | 7.95 | (5) |
| UT | 7/30/20 | 8/11/20 | 3.2 | 1.7 | 5.03 | (5) |
| VA | 7/31/20 | 8/11/20 | 4.1 | 1.8 | 6.93 | (5) |
| VT | 7/30/20 | 8/11/20 | 0.5 | 0 | 1.84 | (5) |
| WA | 7/29/20 | 8/11/20 | 2.1 | 0.81 | 3.7 | (5) |
| WI | 7/30/20 | 8/13/20 | 1.8 | 0.76 | 3.18 | (5) |
| WV | 7/30/20 | 8/13/20 | 1.2 | 0.23 | 2.5 | (5) |
| WY | 7/29/20 | 8/11/20 |  |  |  | (5) |
| AK | 8/12/20 | 8/26/20 | 1.3 | 0.5 | 2.33 | (5) |
| AL | 8/12/20 | 8/26/20 | 7.6 | 5.33 | 9.76 | (5) |
| AR | 8/11/20 | 8/25/20 | 3 | 1.76 | 4.35 | (5) |
| AZ | 8/12/20 | 8/26/20 | 4.7 | 2.7 | 7.61 | (5) |
| CA | 8/13/20 | 8/19/20 | 4.3 | 2.85 | 6.06 | (5) |
| CO | 8/10/20 | 8/25/20 | 3.3 | 1.83 | 4.8 | (5) |
| CT | 8/11/20 | 8/24/20 | 2.4 | 1.45 | 3.52 | (5) |
| DC | 8/13/20 | 8/27/20 | 6.8 | 4.59 | 9.21 | (5) |
| DE | 8/12/20 | 8/27/20 | 8.5 | 5.79 | 11.25 | (5) |
| FL | 8/14/20 | 8/14/20 | 4.5 | 3.15 | 6.06 | (5) |
| GA | 8/13/20 | 8/26/20 | 7.4 | 5.52 | 9.51 | (5) |
| HI | 8/14/20 | 8/26/20 |  |  |  | (5) |
| IA | 8/12/20 | 8/27/20 | 9.4 | 7.12 | 11.63 | (5) |
| ID | 8/12/20 | 8/26/20 | 4.6 | 1.67 | 9.05 | (5) |
| IL | 8/12/20 | 8/27/20 | 4.9 | 3.4 | 6.65 | (5) |
| IN | 8/12/20 | 8/26/20 | 3.1 | 1.53 | 4.76 | (5) |
| KS | 8/11/20 | 8/25/20 | 3.7 | 2.02 | 5.47 | (5) |
| KY | 8/12/20 | 8/26/20 | 3.1 | 2.05 | 4.49 | (5) |
| LA | 8/12/20 | 8/25/20 | 11.8 | 9.27 | 14.81 | (5) |
| MA | 8/12/20 | 8/27/20 | 3.7 | 2.27 | 5.21 | (5) |
| MD | 8/10/20 | 8/26/20 | 7 | 4.92 | 9.33 | (5) |
| ME | 8/12/20 | 8/27/20 | 0.6 | 0 | 1.44 | (5) |
| MI | 8/12/20 | 8/25/20 | 4.2 | 2.88 | 5.76 | (5) |
| MN | 8/11/20 | 8/27/20 | 4.5 | 3.05 | 5.87 | (5) |
| MO | 8/12/20 | 8/21/20 | 3.5 | 2.23 | 5.06 | (5) |
| MS | 8/12/20 | 8/27/20 | 10 | 6.69 | 14.71 | (5) |
| MT | 8/12/20 | 8/24/20 | 1.3 | 0.38 | 2.49 | (5) |
| NC | 8/11/20 | 8/27/20 | 3.8 | 2.33 | 5.29 | (5) |
| ND | 8/12/20 | 8/26/20 | 0.6 | 0 | 1.45 | (5) |
| NE | 8/11/20 | 8/25/20 | 7.9 | 5.51 | 10.61 | (5) |
| NH | 8/13/20 | 8/25/20 | 0.8 | 0.3 | 1.22 | (5) |
| NJ | 8/10/20 | 8/26/20 | 12.2 | 10.15 | 14.53 | (5) |
| NM | 8/10/20 | 8/27/20 | 2.5 | 1.47 | 3.79 | (5) |
| NV | 8/12/20 | 8/26/20 | 7.9 | 6.13 | 9.83 | (5) |
| NY | 8/10/20 | 8/26/20 | 20.6 | 18.04 | 23.14 | (5) |
| OH | 8/12/20 | 8/27/20 | 2.1 | 1.06 | 3.29 | (5) |
| OK | 8/10/20 | 8/18/20 | 4 | 2.72 | 5.51 | (5) |
| OR | 8/10/20 | 8/27/20 | 2.4 | 1.41 | 3.6 | (5) |
| PA | 8/10/20 | 8/26/20 | 10.1 | 7.53 | 13.74 | (5) |
| PR | 8/10/20 | 8/17/20 | 2.2 | 1.18 | 3.26 | (5) |
| RI | 8/12/20 | 8/27/20 | 3.6 | 1.69 | 5.89 | (5) |
| SC | 8/12/20 | 8/27/20 | 6 | 4.37 | 7.95 | (5) |
| SD | 8/12/20 | 8/26/20 | NA | 0 | 4.35 | (5) |
| TN | 8/12/20 | 8/26/20 | 6.7 | 5.11 | 8.57 | (5) |
| TX | 8/12/20 | 8/24/20 | 6.5 | 4.69 | 8.47 | (5) |
| UT | 8/15/20 | 8/25/20 | 5.5 | 2.94 | 8.71 | (5) |
| VA | 8/10/20 | 8/26/20 | 4.7 | 2.51 | 7.22 | (5) |
| VT | 8/13/20 | 8/27/20 | 0.3 | 0 | 0.81 | (5) |
| WA | 8/12/20 | 8/27/20 | 3 | 1.79 | 4.47 | (5) |
| WI | 8/12/20 | 8/27/20 | 3.3 | 2.03 | 4.72 | (5) |
| WV | 8/13/20 | 8/27/20 | 2.2 | 0.52 | 4.9 | (5) |
| WY | 8/13/20 | 8/24/20 | 0.8 | 0 | 3.11 | (5) |
| AK | 8/26/20 | 9/9/20 | 1 | 0.24 | 2.17 | (5) |
| AL | 8/26/20 | 9/8/20 | 9.9 | 7.19 | 12.79 | (5) |
| AR | 8/24/20 | 9/8/20 | 4.9 | 3.5 | 6.41 | (5) |
| AZ | 8/26/20 | 9/9/20 | 4.9 | 3.05 | 7.11 | (5) |
| CA | 8/28/20 | 9/9/20 | 6 | 4.2 | 7.82 | (5) |
| CO | 8/24/20 | 9/4/20 | 3.6 | 2.31 | 5.09 | (5) |
| CT | 8/26/20 | 9/4/20 | 4.3 | 2.9 | 5.69 | (5) |
| DC | 8/27/20 | 9/10/20 | 5 | 2.83 | 7.55 | (5) |
| DE | 8/26/20 | 9/10/20 | 4.5 | 2.42 | 7.12 | (5) |
| FL | 8/27/20 | 9/9/20 | 5.7 | 3.93 | 7.49 | (5) |
| GA | 8/25/20 | 9/10/20 | 8.7 | 6.69 | 11.14 | (5) |
| HI | 8/28/20 | 9/9/20 |  |  |  | (5) |
| IA | 8/25/20 | 9/10/20 | 8.4 | 6.15 | 10.74 | (5) |
| ID | 8/26/20 | 9/10/20 | 5.7 | 2.9 | 9.7 | (5) |
| IL | 8/26/20 | 9/3/20 | 5.6 | 3.9 | 7.67 | (5) |
| IN | 8/26/20 | 9/9/20 | 2.4 | 1.36 | 3.52 | (5) |
| KS | 8/24/20 | 9/3/20 | 2.9 | 1.57 | 4.31 | (5) |
| KY | 8/26/20 | 9/8/20 | 3.1 | 1.91 | 4.33 | (5) |
| LA | 8/26/20 | 9/8/20 | 8.6 | 6.65 | 11.3 | (5) |
| MA | 8/27/20 | 9/5/20 | 3.6 | 2.36 | 5.08 | (5) |
| MD | 8/26/20 | 9/8/20 | 7.9 | 5.72 | 10.35 | (5) |
| ME | 8/26/20 | 9/10/20 | 1.5 | 0.44 | 3.22 | (5) |
| MI | 8/26/20 | 9/3/20 | 3.4 | 2.16 | 4.95 | (5) |
| MN | 8/26/20 | 9/8/20 | 9.2 | 6.71 | 11.95 | (5) |
| MO | 8/24/20 | 9/10/20 | 2.9 | 1.74 | 4.17 | (5) |
| MS | 8/26/20 | 9/10/20 | 8.4 | 4.89 | 12.73 | (5) |
| MT | 8/24/20 | 9/9/20 | 0.9 | 0.23 | 1.82 | (5) |
| NC | 8/26/20 | 9/9/20 | 3.8 | 2.33 | 5.26 | (5) |
| ND | 8/26/20 | 9/9/20 | 0.2 | 0 | 0.99 | (5) |
| NE | 8/24/20 | 9/10/20 | 6.3 | 4.47 | 8.27 | (5) |
| NH | 8/26/20 | 9/3/20 | 1.6 | 0.73 | 2.84 | (5) |
| NJ | 8/26/20 | 9/7/20 | 12.8 | 10.49 | 15.35 | (5) |
| NM | 8/25/20 | 9/8/20 | 3.7 | 2.28 | 5.55 | (5) |
| NV | 8/27/20 | 8/29/20 | 6.5 | 4.63 | 8.58 | (5) |
| NY | 8/26/20 | 9/10/20 | 19.5 | 16.88 | 22.38 | (5) |
| OH | 8/27/20 | 9/10/20 | 5 | 3.17 | 7.14 | (5) |
| OK | 8/24/20 | 9/1/20 | 5.4 | 3.83 | 7.29 | (5) |
| OR | 8/26/20 | 9/10/20 | 1.9 | 0.92 | 2.94 | (5) |
| PA | 8/26/20 | 9/9/20 | 9.5 | 6.58 | 13.56 | (5) |
| PR | 8/24/20 | 9/3/20 | 2.5 | 1.36 | 3.76 | (5) |
| RI | 8/27/20 | 9/10/20 | 3.1 | 1.73 | 4.64 | (5) |
| SC | 8/26/20 | 9/10/20 | 7.2 | 5.12 | 9.57 | (5) |
| SD | 8/26/20 | 9/10/20 | 0.7 | 0 | 2.47 | (5) |
| TN | 8/25/20 | 9/8/20 | 5.4 | 3.62 | 7.31 | (5) |
| TX | 8/25/20 | 9/2/20 | 5.8 | 4.04 | 7.92 | (5) |
| UT | 8/24/20 | 9/8/20 | 4.9 | 2.82 | 7.67 | (5) |
| VA | 8/26/20 | 9/8/20 | 3.5 | 1.97 | 5.26 | (5) |
| VT | 8/26/20 | 9/10/20 | 0.4 | 0 | 0.94 | (5) |
| WA | 8/25/20 | 9/10/20 | 4.5 | 2.63 | 6.94 | (5) |
| WI | 8/25/20 | 9/10/20 | 2.6 | 1.48 | 3.95 | (5) |
| WV | 8/26/20 | 9/10/20 | 1.5 | 0.78 | 2.38 | (5) |
| WY | 8/24/20 | 9/9/20 |  |  |  | (5) |
| AK | 9/9/20 | 9/18/20 | 0.4 | 0.11 | 0.82 | (5) |
| AL | 9/9/20 | 9/18/20 | 8.7 | 6.19 | 11.31 | (5) |
| AR | 9/9/20 | 9/18/20 | 6.3 | 4.6 | 8.03 | (5) |
| AZ | 9/9/20 | 9/23/20 | 5.4 | 3.08 | 8.52 | (5) |
| CA | 9/10/20 | 9/16/20 | 4.9 | 3.17 | 6.85 | (5) |
| CO | 9/9/20 | 9/18/20 | 3.3 | 1.9 | 4.92 | (5) |
| CT | 9/9/20 | 9/14/20 | 3.1 | 2.06 | 4.37 | (5) |
| DC | 9/8/20 | 9/24/20 | 6.5 | 4.47 | 8.29 | (5) |
| DE | 9/9/20 | 9/24/20 | 7.5 | 4.89 | 10.5 | (5) |
| FL | 9/11/20 | 9/11/20 | 8.5 | 6.55 | 10.68 | (5) |
| GA | 9/9/20 | 9/18/20 | 13 | 10.51 | 15.79 | (5) |
| HI | 9/8/20 | 9/22/20 | 0.8 | 0 | 2.18 | (5) |
| IA | 9/9/20 | 9/24/20 | 7.6 | 5.56 | 10.02 | (5) |
| ID | 9/8/20 | 9/18/20 | 5.2 | 2.74 | 8.46 | (5) |
| IL | 9/8/20 | 9/17/20 | 4.5 | 3.1 | 6.07 | (5) |
| IN | 9/9/20 | 9/23/20 | 4 | 1.44 | 8.79 | (5) |
| KS | 9/9/20 | 9/19/20 | 3.5 | 2.31 | 4.84 | (5) |
| KY | 9/9/20 | 9/18/20 | 3.6 | 2.33 | 5.21 | (5) |
| LA | 9/9/20 | 9/19/20 | 12.5 | 10.02 | 15.61 | (5) |
| MA | 9/9/20 | 9/15/20 | 3.7 | 2.23 | 5.16 | (5) |
| MD | 9/10/20 | 9/23/20 | 10.2 | 7.91 | 12.68 | (5) |
| ME | 9/8/20 | 9/24/20 | 0.5 | 0.09 | 0.87 | (5) |
| MI | 9/8/20 | 9/22/20 | 3.7 | 2.54 | 4.93 | (5) |
| MN | 9/9/20 | 9/23/20 | 8 | 4.74 | 11.43 | (5) |
| MO | 9/8/20 | 9/24/20 | 3.5 | 2.15 | 5.01 | (5) |
| MS | 9/9/20 | 9/22/20 | 7.9 | 5.35 | 10.87 | (5) |
| MT | 9/9/20 | 9/23/20 | 2.2 | 0.67 | 4.38 | (5) |
| NC | 9/9/20 | 9/17/20 | 6.8 | 4.83 | 8.87 | (5) |
| ND | 9/9/20 | 9/24/20 | 1.2 | 0 | 3.4 | (5) |
| NE | 9/9/20 | 9/18/20 | 6.7 | 4.8 | 8.88 | (5) |
| NH | 9/9/20 | 9/17/20 | 0.7 | 0.26 | 1.32 | (5) |
| NJ | 9/9/20 | 9/23/20 | 15.1 | 12.65 | 17.63 | (5) |
| NM | 9/8/20 | 9/24/20 | 2.4 | 1.19 | 3.65 | (5) |
| NV | 9/9/20 | 9/12/20 | 7.8 | 5.9 | 9.99 | (5) |
| NY | 9/11/20 | 9/24/20 | 17 | 14.72 | 19.23 | (5) |
| OH | 9/10/20 | 9/22/20 | 2.8 | 1.69 | 4.16 | (5) |
| OK | 9/9/20 | 9/18/20 | 5 | 3.44 | 6.54 | (5) |
| OR | 9/8/20 | 9/22/20 | 2.6 | 1.51 | 4.06 | (5) |
| PA | 9/9/20 | 9/23/20 | 11.1 | 8.85 | 13.71 | (5) |
| PR | 9/8/20 | 9/15/20 | 3 | 1.78 | 4.27 | (5) |
| RI | 9/9/20 | 9/24/20 | 2.7 | 1.15 | 4.91 | (5) |
| SC | 9/9/20 | 9/18/20 | 7.8 | 5.58 | 10.19 | (5) |
| SD | 9/9/20 | 9/23/20 | 1.8 | 0 | 4.82 | (5) |
| TN | 9/8/20 | 9/16/20 | 6.7 | 4.96 | 8.57 | (5) |
| TX | 9/9/20 | 9/24/20 | 8.2 | 6.16 | 10.47 | (5) |
| UT | 9/9/20 | 9/18/20 | 5.1 | 3.29 | 7.9 | (5) |
| VA | 9/10/20 | 9/23/20 | 3.2 | 1.81 | 5.13 | (5) |
| VT | 9/9/20 | 9/24/20 | 1.7 | 0.27 | 4.15 | (5) |
| WA | 9/9/20 | 9/22/20 | 2.5 | 1.29 | 3.83 | (5) |
| WI | 9/9/20 | 9/24/20 | 3.8 | 2.2 | 5.73 | (5) |
| WV | 9/9/20 | 9/22/20 | 1.3 | 0.51 | 2.18 | (5) |
| WY | 9/9/20 | 9/24/20 | 1.5 | 0 | 3.97 | (5) |

| AK | 9/22/20 | 10/7/20 | 0.9 | 0.4 | 1.54 | (36) |
| --- | --- | --- | --- | --- | --- | --- |
| AL | 9/21/20 | 10/7/20 | 8.9 | 6.58 | 11.38 | (36) |
| AR | 9/23/20 | 10/7/20 | 4.3 | 2.78 | 5.74 | (36) |
| AZ | 9/22/20 | 10/7/20 | 7.9 | 2.96 | 14.43 | (36) |
| CA | 9/25/20 | 9/30/20 | 4.1 | 2.61 | 5.64 | (36) |
| CO | 9/24/20 | 10/7/20 | 3 | 1.7 | 4.54 | (36) |
| CT | 9/24/20 | 9/30/20 | 2.5 | 1.47 | 3.8 | (36) |
| DC | 9/23/20 | 10/8/20 | 5 | 3.26 | 7.15 | (36) |
| DE | 9/23/20 | 10/8/20 | 6 | 4.07 | 8.12 | (36) |
| FL | 9/25/20 | 9/25/20 | 7.9 | 6.09 | 9.91 | (36) |
| GA | 9/23/20 | 10/7/20 | 10.4 | 7.68 | 13.1 | (36) |
| HI | 9/22/20 | 10/8/20 | 1.7 | 0.1 | 10.5 | (36) |
| IA | 9/23/20 | 10/7/20 | 8.3 | 6.33 | 10.36 | (36) |
| ID | 9/23/20 | 10/7/20 | 4.9 | 2.73 | 7.17 | (36) |
| IL | 9/23/20 | 10/1/20 | 5.4 | 3.81 | 7.18 | (36) |
| IN | 9/21/20 | 10/7/20 | 6 | 2.14 | 12.02 | (36) |
| KS | 9/22/20 | 10/8/20 | 5 | 3.53 | 6.72 | (36) |
| KY | 9/23/20 | 10/6/20 | 2.3 | 1.29 | 3.46 | (36) |
| LA | 9/23/20 | 10/6/20 | 12 | 9.63 | 14.45 | (36) |
| MA | 9/25/20 | 10/1/20 | 2.8 | 1.76 | 4.04 | (36) |
| MD | 9/21/20 | 10/5/20 | 9.9 | 7.8 | 12.2 | (36) |
| ME | 9/25/20 | 10/8/20 | 0.5 | 0.08 | 1.16 | (36) |
| MI | 9/22/20 | 10/2/20 | 3.4 | 2.33 | 4.48 | (36) |
| MN | 9/23/20 | 10/6/20 | 4.4 | 2.92 | 5.95 | (36) |
| MO | 9/21/20 | 10/2/20 | 5.9 | 4.19 | 8.12 | (36) |
| MS | 9/23/20 | 10/6/20 | 5.5 | 3.82 | 7.31 | (36) |
| MT | 9/23/20 | 10/8/20 | 2.7 | 0.6 | 6.52 | (36) |
| NC | 9/23/20 | 10/2/20 | 6.7 | 4.74 | 8.89 | (36) |
| NE | 9/23/20 | 10/7/20 | 10 | 7.16 | 12.88 | (36) |
| NH | 9/25/20 | 10/1/20 | 1 | 0.28 | 2.23 | (36) |
| NJ | 9/21/20 | 10/7/20 | 16 | 13.57 | 18.74 | (36) |
| NM | 9/23/20 | 10/6/20 | 3.6 | 2.29 | 5.07 | (36) |
| NV | 9/23/20 | 9/26/20 | 7.8 | 5.85 | 9.94 | (36) |
| NY | 9/20/20 | 10/7/20 | 22.8 | 19.2 | 26.54 | (36) |
| OH | 9/23/20 | 10/8/20 | 2.4 | 1.26 | 3.64 | (36) |
| OK | 9/23/20 | 9/29/20 | 5.6 | 3.78 | 7.36 | (36) |
| OR | 9/20/20 | 10/7/20 | 2.5 | 0.57 | 5.68 | (36) |
| PA | 9/20/20 | 10/5/20 | 10.7 | 8.17 | 13.78 | (36) |
| RI | 9/25/20 | 10/8/20 | 4.2 | 2.57 | 6.27 | (36) |
| SC | 9/23/20 | 10/7/20 | 9.1 | 6.39 | 11.78 | (36) |
| TN | 9/23/20 | 10/7/20 | 9.1 | 6.83 | 11.4 | (36) |
| TX | 9/23/20 | 10/5/20 | 7.2 | 5.19 | 9.56 | (36) |
| UT | 9/23/20 | 10/7/20 | 5.3 | 3.61 | 7.31 | (36) |
| VA | 9/21/20 | 10/5/20 | 5 | 2.34 | 9.26 | (36) |
| VT | 9/25/20 | 10/8/20 | 0.5 | 0 | 1.19 | (36) |
| WA | 9/22/20 | 10/8/20 | 2.3 | 1.25 | 3.6 | (36) |
| WI | 9/23/20 | 10/6/20 | 3.1 | 1.96 | 4.62 | (36) |
| WV | 9/23/20 | 10/8/20 | 3.2 | 1.11 | 6.18 | (36) |
| WY | 9/23/20 | 10/7/20 | 4.2 | 0.68 | 9.25 | (36) |
| AK | 10/6/20 | 10/21/20 | 1.5 | 0.79 | 2.46 | (36) |
| AL | 10/7/20 | 10/21/20 | 12.3 | 10 | 14.99 | (36) |
| AR | 10/7/20 | 10/22/20 | 9 | 7.14 | 11.38 | (36) |
| AZ | 10/7/20 | 10/21/20 | 4.8 | 2.55 | 7.89 | (36) |
| CA | 10/8/20 | 10/13/20 | 4.5 | 2.9 | 6.42 | (36) |
| CO | 10/8/20 | 10/19/20 | 4.4 | 2.82 | 6.51 | (36) |
| CT | 10/8/20 | 10/13/20 | 2.3 | 1.32 | 3.52 | (36) |
| DC | 10/7/20 | 10/22/20 | 6.8 | 4.99 | 8.89 | (36) |
| DE | 10/7/20 | 10/22/20 | 7.4 | 4.95 | 9.88 | (36) |
| FL | 10/9/20 | 10/9/20 | 8 | 6.01 | 10.22 | (36) |
| GA | 10/6/20 | 10/13/20 | 8.5 | 6.17 | 11.18 | (36) |
| HI | 10/6/20 | 10/22/20 | 2 | 0.55 | 4.19 | (36) |
| IA | 10/7/20 | 10/22/20 | 13 | 9.28 | 18.19 | (36) |
| ID | 10/5/20 | 10/21/20 | 4.2 | 2.64 | 6.02 | (36) |
| IL | 10/7/20 | 10/13/20 | 5.8 | 4.14 | 7.54 | (36) |
| IN | 10/7/20 | 10/20/20 | 4.6 | 2.27 | 7.83 | (36) |
| KS | 10/6/20 | 10/22/20 | 7.1 | 5.16 | 9.18 | (36) |
| KY | 10/7/20 | 10/16/20 | 3.3 | 1.95 | 4.72 | (36) |
| LA | 10/6/20 | 10/19/20 | 9.8 | 7.36 | 12.46 | (36) |
| MA | 10/9/20 | 10/13/20 | 2.6 | 1.5 | 3.88 | (36) |
| MD | 10/10/20 | 10/21/20 | 11.4 | 8.27 | 14.84 | (36) |
| ME | 10/9/20 | 10/22/20 | 0.6 | 0.16 | 1.25 | (36) |
| MI | 10/6/20 | 10/16/20 | 3.2 | 1.96 | 4.55 | (36) |
| MN | 10/6/20 | 10/22/20 | 7.5 | 5.36 | 10.01 | (36) |
| MO | 10/7/20 | 10/15/20 | 6.5 | 4.37 | 8.64 | (36) |
| MS | 10/5/20 | 10/20/20 | 13.1 | 9.4 | 17.24 | (36) |
| MT | 10/7/20 | 10/21/20 | 3.2 | 1.57 | 5.01 | (36) |
| NC | 10/6/20 | 10/19/20 | 3.5 | 2.05 | 5.04 | (36) |
| NE | 10/7/20 | 10/21/20 | 11.3 | 8.6 | 14.13 | (36) |
| NH | 10/9/20 | 10/15/20 | 0.8 | 0.31 | 1.42 | (36) |
| NJ | 10/7/20 | 10/21/20 | 12.4 | 10.02 | 15.17 | (36) |
| NM | 10/6/20 | 10/20/20 | 4.8 | 3.26 | 6.62 | (36) |
| NV | 10/6/20 | 10/16/20 | 8.2 | 6.4 | 10.06 | (36) |
| NY | 10/9/20 | 10/21/20 | 21.9 | 18.72 | 25.25 | (36) |
| OH | 10/6/20 | 10/22/20 | 1.8 | 0.94 | 2.75 | (36) |
| OK | 10/7/20 | 10/14/20 | 7.4 | 5.08 | 9.71 | (36) |
| OR | 10/7/20 | 10/19/20 | 2.1 | 0.87 | 3.59 | (36) |
| PA | 10/8/20 | 10/21/20 | 10.5 | 8.39 | 13.16 | (36) |
| RI | 10/9/20 | 10/22/20 | 2.4 | 1.43 | 3.54 | (36) |
| SC | 10/7/20 | 10/21/20 | 8 | 5.78 | 10.49 | (36) |
| TN | 10/7/20 | 10/15/20 | 9.9 | 7.49 | 12.26 | (36) |
| TX | 10/5/20 | 10/19/20 | 10.4 | 8.15 | 12.98 | (36) |
| UT | 10/5/20 | 10/21/20 | 9.1 | 6.3 | 11.99 | (36) |
| VA | 10/9/20 | 10/21/20 | 4.1 | 2.41 | 6.2 | (36) |
| VT | 10/9/20 | 10/22/20 | 0.4 | 0 | 1.22 | (36) |
| WA | 10/7/20 | 10/21/20 | 2 | 0.91 | 3.21 | (36) |
| WI | 10/7/20 | 10/22/20 | 5 | 3.41 | 6.98 | (36) |
| WV | 10/7/20 | 10/22/20 | 2.2 | 1.29 | 3.3 | (36) |
| WY | 10/8/20 | 10/21/20 | 2.6 | 0.46 | 5.78 | (36) |
| AK | 10/30/20 | 11/13/20 | 6.3 | 3.68 | 10.57 | (36) |
| AL | 10/26/20 | 11/9/20 | 9.2 | 7.57 | 10.99 | (36) |
| AR | 10/26/20 | 11/6/20 | 6.5 | 5.15 | 7.98 | (36) |
| AZ | 10/26/20 | 11/10/20 | 9.6 | 8.08 | 11.12 | (36) |
| CA | 10/26/20 | 11/12/20 | 6.6 | 5.09 | 8.12 | (36) |
| CO | 10/30/20 | 11/13/20 | 5 | 3.35 | 6.8 | (36) |
| CT | 11/2/20 | 11/14/20 | 3.5 | 2.09 | 4.99 | (36) |
| DC | 10/27/20 | 11/11/20 | 8.8 | 6.97 | 10.67 | (36) |
| DE | 10/26/20 | 11/9/20 | 10.4 | 7.72 | 13.62 | (36) |
| FL | 10/27/20 | 11/4/20 | 7.6 | 6.02 | 9.11 | (36) |
| GA | 10/27/20 | 11/13/20 | 12.4 | 10.01 | 14.88 | (36) |
| HI | 10/29/20 | 11/15/20 | 2.9 | 1.66 | 4.68 | (36) |
| IA | 10/28/20 | 11/13/20 | 11.9 | 9.37 | 14.58 | (36) |
| ID | 10/30/20 | 11/13/20 | 6.3 | 4.19 | 8.96 | (36) |
| IL | 10/26/20 | 11/7/20 | 12.9 | 10.87 | 14.94 | (36) |
| IN | 10/29/20 | 11/11/20 | 6.3 | 4.25 | 8.59 | (36) |
| KS | 11/1/20 | 11/15/20 | 5.5 | 4.17 | 7.06 | (36) |
| KY | 10/26/20 | 11/10/20 | 7.5 | 5.88 | 9.43 | (36) |
| LA | 10/28/20 | 11/14/20 | 9.7 | 7.49 | 11.78 | (36) |
| MA | 11/2/20 | 11/11/20 | 3.8 | 2.48 | 5.2 | (36) |
| MD | 10/29/20 | 11/11/20 | 7.2 | 5.3 | 9.36 | (36) |
| ME | 11/2/20 | 11/13/20 | 1 | 0.33 | 1.79 | (36) |
| MI | 10/26/20 | 11/13/20 | 8.6 | 6.43 | 11.05 | (36) |
| MN | 10/29/20 | 11/13/20 | 8.4 | 6.79 | 10.14 | (36) |
| MO | 11/2/20 | 11/10/20 | 6.1 | 4.32 | 7.97 | (36) |
| MS | 10/25/20 | 11/9/20 | 11 | 9.31 | 13.11 | (36) |
| MT | 10/30/20 | 11/13/20 | 7.7 | 4.98 | 11 | (36) |
| NC | 10/27/20 | 11/10/20 | 7.5 | 5.89 | 8.92 | (36) |
| NE | 10/30/20 | 11/14/20 | 11.9 | 9.58 | 14.2 | (36) |
| NH | 11/2/20 | 11/7/20 | 1.1 | 0.38 | 1.97 | (36) |
| NJ | 10/29/20 | 11/11/20 | 11.9 | 10.04 | 13.87 | (36) |
| NM | 10/30/20 | 11/15/20 | 7.5 | 5.68 | 9.39 | (36) |
| NV | 10/25/20 | 11/9/20 | 8.5 | 6.56 | 10.51 | (36) |
| NY | 10/30/20 | 11/15/20 | 13 | 10.7 | 15.36 | (36) |
| OH | 10/25/20 | 11/10/20 | 7.3 | 5.84 | 8.79 | (36) |
| OK | 10/31/20 | 11/13/20 | 6 | 4.55 | 7.7 | (36) |
| OR | 10/30/20 | 11/13/20 | 2.3 | 1.18 | 3.58 | (36) |
| PA | 10/26/20 | 11/9/20 | 6.3 | 4.95 | 7.69 | (36) |
| RI | 11/2/20 | 11/13/20 | 3.4 | 2.08 | 4.92 | (36) |
| SC | 10/27/20 | 11/11/20 | 8.4 | 6.94 | 9.99 | (36) |
| SD | 10/27/20 | 11/10/20 | 8.8 | 3.94 | 15.36 | (36) |
| TN | 10/26/20 | 11/10/20 | 10.7 | 8.75 | 12.57 | (36) |
| TX | 10/29/20 | 11/11/20 | 15.9 | 11.55 | 23.68 | (36) |
| UT | 10/25/20 | 11/9/20 | 6.9 | 4.22 | 10.79 | (36) |
| VA | 10/29/20 | 11/11/20 | 3.6 | 2.07 | 5.6 | (36) |
| VT | 11/2/20 | 11/13/20 | 0.4 | 0.04 | 0.98 | (36) |
| WA | 11/1/20 | 11/13/20 | 3.4 | 2.45 | 4.44 | (36) |
| WI | 10/26/20 | 11/10/20 | 15.3 | 12.24 | 18.9 | (36) |
| WV | 10/29/20 | 11/15/20 | 5.5 | 3.03 | 9.35 | (36) |
| WY | 10/26/20 | 11/10/20 | 4.8 | 2.36 | 7.62 | (36) |

**Table B.** State-wide seroprevalence validation data

| **State** | **Min date** | **Max date** | **Point est. (%)** | **LCL (%)** | **UCL (%)** | **Source** |
| --- | --- | --- | --- | --- | --- | --- |
| AK | 11/14/20 | 11/28/20 | 6.5 | 4.28 | 9.14 | (36) |
| AL | 11/14/20 | 11/24/20 | 9 | 7.15 | 10.98 | (36) |
| AR | 11/9/20 | 11/25/20 | 7.5 | 6.25 | 8.89 | (36) |
| AZ | 11/9/20 | 11/25/20 | 11.4 | 9.47 | 13.63 | 36 |
| CA | 11/9/20 | 11/20/20 | 7.9 | 6.09 | 9.63 | (36) |
| CO | 11/13/20 | 11/27/20 | 7.1 | 4.72 | 9.97 | (36) |
| CT | 11/16/20 | 11/28/20 | 4.6 | 3.28 | 5.98 | (36) |
| DC | 11/10/20 | 11/23/20 | 11.4 | 9.33 | 13.42 | (36) |
| DE | 11/10/20 | 11/27/20 | 8.1 | 5.97 | 10.3 | (36) |
| FL | 11/9/20 | 11/20/20 | 10.2 | 8.66 | 11.82 | (36) |
| GA | 11/15/20 | 11/23/20 | 10.3 | 8.09 | 12.58 | (36) |
| HI | 11/13/20 | 11/25/20 | 2.8 | 1.34 | 4.31 | (36) |
| IA | 11/13/20 | 11/27/20 | 14.7 | 11.02 | 18.56 | (36) |
| ID | 11/17/20 | 11/27/20 | 13.4 | 9.61 | 17.73 | (36) |
| IL | 11/14/20 | 11/25/20 | 16.2 | 14.64 | 17.83 | (36) |
| IN | 11/12/20 | 11/25/20 | 6.2 | 3.79 | 8.63 | (36) |
| KS | 11/9/20 | 11/28/20 | 8.2 | 6.39 | 10.35 | (36) |
| KY | 11/9/20 | 11/25/20 | 7.7 | 5.86 | 9.79 | (36) |
| LA | 11/12/20 | 11/26/20 | 9.3 | 6.92 | 11.79 | (36) |
| MA | 11/16/20 | 11/28/20 | 4.5 | 3.29 | 5.87 | (36) |
| MD | 11/12/20 | 11/26/20 | 9.5 | 7.51 | 11.43 | (36) |
| ME | 11/16/20 | 11/27/20 | 0.6 | 0.1 | 1.29 | (36) |
| MI | 11/9/20 | 11/25/20 | 8.8 | 6.47 | 12.1 | (36) |
| MN | 11/12/20 | 11/27/20 | 11.4 | 9.23 | 13.83 | (36) |
| MO | 11/9/20 | 11/25/20 | 10.3 | 8.14 | 12.86 | (36) |
| MS | 11/9/20 | 11/24/20 | 13.6 | 11.65 | 15.61 | (36) |
| MT | 11/11/20 | 11/25/20 | 9.1 | 6 | 12.5 | (36) |
| NC | 11/9/20 | 11/23/20 | 8.4 | 6.49 | 10.45 | (36) |
| NE | 11/13/20 | 11/27/20 | 16.3 | 13.06 | 19.61 | (36) |
| NH | 11/16/20 | 11/27/20 | 1.6 | 0.72 | 2.54 | (36) |
| NJ | 11/12/20 | 11/26/20 | 15.1 | 12.92 | 17.35 | (36) |
| NM | 11/12/20 | 11/29/20 | 8.8 | 6.72 | 11.11 | (36) |
| NV | 11/9/20 | 11/25/20 | 12 | 9.18 | 14.98 | (36) |
| NY | 11/11/20 | 11/28/20 | 9.3 | 7.21 | 11.24 | (36) |
| OH | 11/13/20 | 11/25/20 | 12.8 | 10.54 | 15.16 | (36) |
| OK | 11/14/20 | 11/17/20 | 6.5 | 4.62 | 8.52 | (36) |
| OR | 11/16/20 | 11/20/20 | 4.1 | 1.67 | 7.09 | (36) |
| PA | 11/9/20 | 11/27/20 | 7.6 | 6.36 | 8.83 | (36) |
| RI | 11/16/20 | 11/27/20 | 5.3 | 3.82 | 6.85 | (36) |
| SC | 11/10/20 | 11/23/20 | 10.6 | 8.89 | 12.37 | (36) |
| SD | 11/10/20 | 11/25/20 | 12 | 4.77 | 20.21 | (36) |
| TN | 11/9/20 | 11/25/20 | 12 | 10.29 | 13.81 | (36) |
| TX | 11/12/20 | 11/25/20 | 14.3 | 10.62 | 18.52 | (36) |
| UT | 11/12/20 | 11/25/20 | 12.2 | 8.66 | 15.38 | (36) |
| VA | 11/12/20 | 11/26/20 | 4.6 | 2.98 | 6.56 | (36) |
| VT | 11/16/20 | 11/28/20 | 0.6 | 0 | 1.68 | (36) |
| WA | 11/19/20 | 11/23/20 | 3 | 1.01 | 5.38 | (36) |
| WI | 11/9/20 | 11/25/20 | 15.6 | 13.39 | 18.13 | (36) |
| WV | 11/16/20 | 11/27/20 | 3.6 | 1.82 | 5.78 | (36) |
| WY | 11/9/20 | 11/25/20 | 9.1 | 4.29 | 14.94 | (36) |
| AK | 11/23/20 | 12/7/20 | 8.8 | 6.17 | 11.53 | (36) |
| AL | 11/23/20 | 12/8/20 | 11.7 | 9.74 | 14.02 | (36) |
| AR | 11/25/20 | 12/9/20 | 9.2 | 7.21 | 11.11 | (36) |
| AZ | 11/30/20 | 12/8/20 | 12.2 | 9.85 | 14.69 | (36) |
| CA | 11/23/20 | 12/3/20 | 8.9 | 7.07 | 10.87 | (36) |
| CO | 11/26/20 | 12/11/20 | 9.8 | 8 | 11.4 | (36) |
| CT | 11/30/20 | 12/11/20 | 4.9 | 3.49 | 6.45 | (36) |
| DC | 11/23/20 | 12/5/20 | 13.2 | 10.78 | 15.31 | (36) |
| DE | 11/24/20 | 12/10/20 | 7.8 | 5.91 | 9.81 | (36) |
| FL | 11/30/20 | 12/2/20 | 11.3 | 9 | 13.5 | (36) |
| GA | 11/25/20 | 12/10/20 | 15 | 12.59 | 17.7 | (36) |
| HI | 11/27/20 | 12/12/20 | 4.5 | 2.04 | 7.24 | (36) |
| IA | 11/25/20 | 12/11/20 | 19.7 | 16.18 | 23.39 | (36) |
| ID | 11/27/20 | 12/8/20 | 10.7 | 7.31 | 14.51 | (36) |
| IL | 11/27/20 | 12/8/20 | 23.4 | 21.32 | 25.62 | (36) |
| IN | 11/26/20 | 12/9/20 | 8.6 | 6.14 | 11.87 | (36) |
| KS | 11/24/20 | 12/12/20 | 9.7 | 7.68 | 11.79 | (36) |
| KY | 11/25/20 | 12/9/20 | 10.9 | 8.95 | 13.04 | (36) |
| LA | 11/27/20 | 12/12/20 | 10.2 | 8.15 | 12.13 | (36) |
| MA | 11/30/20 | 12/12/20 | 6.2 | 4.76 | 7.82 | (36) |
| MD | 11/26/20 | 12/10/20 | 10.9 | 8.32 | 13.81 | (36) |
| ME | 11/30/20 | 12/11/20 | 1.3 | 0.34 | 2.53 | (36) |
| MI | 11/24/20 | 12/9/20 | 10.3 | 7.88 | 12.68 | (36) |
| MN | 11/25/20 | 12/10/20 | 14 | 11.71 | 16.52 | (36) |
| MO | 11/27/20 | 12/9/20 | 14.8 | 12.15 | 17.4 | (36) |
| MS | 11/23/20 | 12/8/20 | 18.3 | 15.53 | 21.34 | (36) |
| MT | 11/29/20 | 12/11/20 | 9.7 | 6.61 | 12.66 | (36) |
| NC | 11/23/20 | 12/1/20 | 7.3 | 5.6 | 9.23 | (36) |
| NE | 11/27/20 | 12/11/20 | 15.5 | 12.43 | 18.79 | (36) |
| NH | 11/30/20 | 12/5/20 | 1.9 | 1.05 | 2.93 | (36) |
| NJ | 11/27/20 | 12/10/20 | 15.5 | 13.08 | 18.29 | (36) |
| NM | 11/28/20 | 12/12/20 | 12.9 | 10.57 | 15.1 | (36) |
| NV | 11/27/20 | 12/8/20 | 13.2 | 10.31 | 16.23 | (36) |
| NY | 11/23/20 | 12/12/20 | 12.2 | 10.13 | 14.58 | (36) |
| OH | 11/25/20 | 12/9/20 | 16.5 | 14.4 | 18.73 | (36) |
| OK | 11/30/20 | 12/10/20 | 6.7 | 5.13 | 8.24 | (36) |
| OR | 11/30/20 | 12/11/20 | 3.6 | 2.32 | 5.05 | (36) |
| PA | 11/27/20 | 12/9/20 | 10.6 | 8.58 | 12.68 | (36) |
| RI | 11/30/20 | 12/12/20 | 8.7 | 6.95 | 10.66 | (36) |
| SC | 11/23/20 | 12/7/20 | 9.4 | 7.63 | 11.56 | (36) |
| SD | 11/25/20 | 12/9/20 | 23.3 | 13.47 | 34.75 | (36) |
| TN | 11/25/20 | 12/8/20 | 14.9 | 12.52 | 17.19 | (36) |
| TX | 11/26/20 | 12/9/20 | 18.1 | 13.95 | 23.3 | (36) |
| UT | 11/23/20 | 12/7/20 | 21.6 | 14.83 | 27.87 | (36) |
| VA | 11/26/20 | 12/10/20 | 5.7 | 3.45 | 8.02 | (36) |
| VT | 11/30/20 | 12/11/20 | 0.8 | 0.04 | 1.8 | (36) |
| WA | 11/23/20 | 12/11/20 | 5.5 | 3.91 | 7.32 | (36) |
| WI | 11/25/20 | 12/7/20 | 20.3 | 16.45 | 24.31 | (36) |
| WV | 11/28/20 | 12/12/20 | 5.1 | 3.26 | 7.47 | (36) |
| WY | 11/24/20 | 12/9/20 | 13.1 | 8.1 | 18.82 | (36) |
| AK | 12/10/20 | 12/21/20 | 11.1 | 7.71 | 15 | (36) |
| AL | 12/14/20 | 12/26/20 | 15.5 | 13.32 | 17.9 | (36) |
| AR | 12/14/20 | 12/27/20 | 13.1 | 10.97 | 15.2 | (36) |
| AZ | 12/14/20 | 12/27/20 | 12.6 | 10.9 | 14.43 | (36) |
| CA | 12/14/20 | 12/27/20 | 10.4 | 8.33 | 12.49 | (36) |
| CO | 12/11/20 | 12/18/20 | 11.5 | 8.96 | 14.46 | (36) |
| CT | 12/14/20 | 12/26/20 | 8.5 | 6.77 | 10.28 | (36) |
| DC | 12/14/20 | 12/22/20 | 12.5 | 9.87 | 14.91 | (36) |
| DE | 12/14/20 | 12/22/20 | 9.9 | 7.65 | 11.98 | (36) |
| FL | 12/14/20 | 12/24/20 | 13 | 11.03 | 15.09 | (36) |
| GA | 12/14/20 | 12/18/20 | 16.3 | 13.63 | 19.49 | (36) |
| HI | 12/10/20 | 12/26/20 | 3 | 1.64 | 4.33 | (36) |
| IA | 12/12/20 | 12/24/20 | 19.3 | 15.48 | 23.31 | (36) |
| ID | 12/8/20 | 12/21/20 | 18 | 13.38 | 22.45 | (36) |
| IL | 12/14/20 | 12/26/20 | 23.1 | 20.66 | 25.55 | (36) |
| IN | 12/10/20 | 12/22/20 | 11.2 | 8.57 | 13.8 | (36) |
| KS | 12/11/20 | 12/23/20 | 12.3 | 10.23 | 14.41 | (36) |
| KY | 12/14/20 | 12/27/20 | 11.7 | 10.01 | 13.72 | (36) |
| LA | 12/10/20 | 12/24/20 | 10.6 | 8.34 | 13.26 | (36) |
| MA | 12/14/20 | 12/26/20 | 5.9 | 4.51 | 7.43 | (36) |
| MD | 12/10/20 | 12/23/20 | 15.2 | 12.7 | 17.8 | (36) |
| ME | 12/14/20 | 12/26/20 | 2.3 | 0.7 | 5.51 | (36) |
| MI | 12/14/20 | 12/26/20 | 15.1 | 12.53 | 18.16 | (36) |
| MN | 12/11/20 | 12/23/20 | 15.2 | 12.58 | 17.9 | (36) |
| MO | 12/14/20 | 12/26/20 | 18.6 | 14.58 | 22.33 | (36) |
| MS | 12/14/20 | 12/26/20 | 17.7 | 14.03 | 21.59 | (36) |
| MT | 12/10/20 | 12/23/20 | 14.8 | 10.51 | 18.89 | (36) |
| NC | 12/14/20 | 12/23/20 | 8.1 | 6.18 | 10.2 | (36) |
| NE | 12/8/20 | 12/26/20 | 23 | 19.12 | 27.01 | (36) |
| NH | 12/14/20 | 12/24/20 | 3.2 | 1.88 | 4.83 | (36) |
| NJ | 12/10/20 | 12/23/20 | 16.8 | 14.29 | 19.16 | (36) |
| NM | 12/10/20 | 12/22/20 | 20.2 | 16.98 | 23.66 | (36) |
| NV | 12/14/20 | 12/27/20 | 16.4 | 14.43 | 18.46 | (36) |
| NY | 12/11/20 | 12/23/20 | 9.7 | 7.91 | 11.77 | (36) |
| OH | 12/14/20 | 12/22/20 | 17.9 | 15.86 | 20.03 | (36) |
| OK | 12/10/20 | 12/19/20 | 11.6 | 9.39 | 13.82 | (36) |
| OR | 12/11/20 | 12/15/20 | 4.6 | 2.55 | 6.85 | (36) |
| PA | 12/14/20 | 12/27/20 | 11.7 | 9.56 | 13.9 | (36) |
| RI | 12/14/20 | 12/26/20 | 11.8 | 9.18 | 14.52 | (36) |
| SC | 12/14/20 | 12/23/20 | 13.3 | 10.86 | 16.45 | (36) |
| TN | 12/14/20 | 12/26/20 | 18 | 14.14 | 22.25 | (36) |
| TX | 12/10/20 | 12/23/20 | 16.4 | 13.96 | 19.41 | (36) |
| UT | 12/14/20 | 12/27/20 | 19.7 | 16.11 | 24.18 | (36) |
| VA | 12/10/20 | 12/23/20 | 5.7 | 3.78 | 8.54 | (36) |
| VT | 12/14/20 | 12/24/20 | 1.4 | 0 | 3.17 | (36) |
| WA | 12/11/20 | 12/15/20 | 5 | 2.7 | 7.76 | (36) |
| WI | 12/14/20 | 12/27/20 | 24 | 19.81 | 28.42 | (36) |
| WV | 12/10/20 | 12/26/20 | 5.9 | 3.93 | 8.11 | (36) |
| WY | 12/14/20 | 12/24/20 | 18.5 | 11.19 | 26.47 | (36) |
| AK | 12/23/20 | 1/9/21 | 10.2 | 8.11 | 12.54 | (36) |
| AL | 12/28/20 | 1/8/21 | 20.4 | 18.07 | 23.06 | (36) |
| AR | 12/28/20 | 1/10/21 | 13.8 | 11.74 | 15.98 | (36) |
| AZ | 12/28/20 | 1/7/21 | 17 | 14.73 | 19.22 | (36) |
| CA | 12/28/20 | 1/10/21 | 16.4 | 14.06 | 18.96 | (36) |
| CO | 12/25/20 | 1/6/21 | 12.6 | 9.88 | 15.61 | (36) |
| CT | 12/28/20 | 1/9/21 | 8.1 | 6.42 | 9.65 | (36) |
| DC | 12/28/20 | 1/6/21 | 14.6 | 11.9 | 17.14 | (36) |
| DE | 12/28/20 | 1/9/21 | 9.9 | 8.02 | 11.91 | (36) |
| FL | 12/28/20 | 1/9/21 | 18.6 | 16.21 | 20.93 | (36) |
| GA | 12/27/20 | 12/31/20 | 19.7 | 16.78 | 22.54 | (36) |
| HI | 12/23/20 | 1/9/21 | 2.8 | 1.61 | 4.3 | (36) |
| IA | 12/26/20 | 1/8/21 | 20.6 | 16.88 | 24.62 | (36) |
| ID | 12/24/20 | 1/9/21 | 15.4 | 11.71 | 19.46 | (36) |
| IL | 12/28/20 | 1/7/21 | 28.4 | 25.35 | 31.34 | (36) |
| IN | 12/26/20 | 1/7/21 | 15.6 | 11.65 | 20.24 | (36) |
| KS | 12/26/20 | 1/7/21 | 14.2 | 11.48 | 16.61 | (36) |
| KY | 12/28/20 | 1/10/21 | 13.5 | 11.39 | 15.84 | (36) |
| LA | 12/24/20 | 1/6/21 | 13.3 | 10.5 | 15.88 | (36) |
| MA | 12/28/20 | 1/9/21 | 9.5 | 7.49 | 11.49 | (36) |
| MD | 12/24/20 | 1/7/21 | 16.8 | 13.82 | 20.31 | (36) |
| ME | 12/28/20 | 1/8/21 | 2.5 | 1.35 | 3.98 | (36) |
| MI | 12/28/20 | 1/10/21 | 17.5 | 14.47 | 20.16 | (36) |
| MN | 12/22/20 | 1/7/21 | 15.9 | 13.25 | 18.62 | (36) |
| MO | 12/28/20 | 1/9/21 | 16.9 | 13.83 | 20.2 | (36) |
| MS | 12/28/20 | 1/10/21 | 21 | 18.29 | 23.67 | (36) |
| MT | 12/25/20 | 1/8/21 | 18.4 | 14.46 | 22.8 | (36) |
| NC | 12/29/20 | 1/7/21 | 12.7 | 10.73 | 14.68 | (36) |
| NE | 12/26/20 | 1/9/21 | 21.1 | 17.7 | 24.36 | (36) |
| NH | 12/28/20 | 12/31/20 | 4.8 | 3.17 | 6.78 | (36) |
| NJ | 12/24/20 | 1/7/21 | 19.8 | 17.19 | 22.57 | (36) |
| NM | 12/22/20 | 1/6/21 | 16.1 | 13.28 | 19.31 | (36) |
| NV | 12/28/20 | 1/7/21 | 19.3 | 16.89 | 21.96 | (36) |
| NY | 12/24/20 | 1/6/21 | 12.9 | 10.73 | 15.13 | (36) |
| OH | 12/28/20 | 1/9/21 | 23.8 | 21.09 | 26.22 | (36) |
| OK | 12/28/20 | 12/31/20 | 12.5 | 9.95 | 15.23 | (36) |
| OR | 12/24/20 | 1/9/21 | 7.2 | 5.54 | 9 | (36) |
| PA | 12/28/20 | 1/10/21 | 16.5 | 13.94 | 19.55 | (36) |
| RI | 12/28/20 | 1/9/21 | 11.4 | 9.2 | 13.69 | (36) |
| SC | 12/30/20 | 1/9/21 | 14.4 | 12.13 | 16.5 | (36) |
| TN | 12/28/20 | 1/9/21 | 18.2 | 15.74 | 20.87 | (36) |
| TX | 12/23/20 | 1/7/21 | 20.2 | 16.69 | 23.57 | (36) |
| UT | 12/28/20 | 1/7/21 | 17.4 | 13.46 | 21.69 | (36) |
| VA | 12/24/20 | 1/7/21 | 8.7 | 5.85 | 11.63 | (36) |
| VT | 12/28/20 | 1/9/21 | 1.4 | 0.23 | 3.22 | (36) |
| WA | 12/23/20 | 1/8/21 | 5.1 | 3.48 | 7.05 | (36) |
| WI | 12/28/20 | 1/10/21 | 27.3 | 23.74 | 30.87 | (36) |
| WV | 12/25/20 | 1/9/21 | 8.5 | 5.38 | 12 | (36) |
| WY | 12/28/20 | 1/9/21 | 20.9 | 10.99 | 32.39 | (36) |

**Table C:** Posterior distributions and convergence diagnostic of *n* and SP_o_ for individual states (random effects)

|  | **Primary model** |  |  |  | **Geometric mean model** |  |
| --- | --- | --- | --- | --- | --- | --- |
| **State** | ***n*: median [95% CrI]** | ***n*: PSRF** | **SPo/N (%): median [95% CrI]** | **SPo/N (%): PSRF** | **SPo/N (%): median [95% CrI]** | **SPo/N (%): PSRF** |
| AL | 0.52 [0.43 - 0.67] | 1.1 | 0.96 [0.02 - 4.35] | 1.01 | 2.16 [0.34 - 4.32] | 1.01 |
| AK | 0.58 [0.47 - 0.73] | 1.09 | 0.13 [0.01 - 0.48] | 1 | 0.14 [0.01 - 0.48] | 1 |
| AZ | 0.55 [0.45 - 0.69] | 1.1 | 0.55 [0.02 - 2.69] | 1 | 0.72 [0.04 - 2.5] | 1 |
| AR | 0.54 [0.45 - 0.68] | 1.1 | 0.39 [0.01 - 1.88] | 1 | 0.51 [0.03 - 1.61] | 1.02 |
| CA | 0.53 [0.42 - 0.69] | 1.09 | 1.28 [0.04 - 3.07] | 1.01 | 1.7 [0.67 - 2.97] | 1.01 |
| CO | 0.54 [0.46 - 0.68] | 1.1 | 0.42 [0.02 - 1.79] | 1 | 0.45 [0.03 - 1.36] | 1.01 |
| CT | 0.57 [0.46 - 0.73] | 1.08 | 1.1 [0.04 - 2.52] | 1.01 | 0.78 [0.08 - 1.68] | 1.02 |
| DE | 0.51 [0.4 - 0.68] | 1.09 | 1.44 [0.04 - 4.47] | 1.01 | 2.7 [1.04 - 4.69] | 1.01 |
| DC | 0.52 [0.42 - 0.67] | 1.1 | 0.98 [0.02 - 3.76] | 1.01 | 1.81 [0.33 - 3.59] | 1.03 |
| FL | 0.53 [0.43 - 0.69] | 1.09 | 0.87 [0.02 - 3.01] | 1 | 1.39 [0.15 - 2.92] | 1.01 |
| GA | 0.5 [0.41 - 0.64] | 1.1 | 0.93 [0.02 - 4.37] | 1.01 | 2.82 [1.05 - 4.82] | 1.01 |
| HI | 0.55 [0.45 - 0.7] | 1.07 | 0.17 [0.01 - 0.89] | 1 | 0.21 [0.02 - 0.92] | 1 |
| ID | 0.57 [0.48 - 0.71] | 1.08 | 0.65 [0.02 - 2.56] | 1 | 0.49 [0.04 - 1.81] | 1.02 |
| IL | 0.52 [0.42 - 0.69] | 1.08 | 1.9 [0.05 - 4.48] | 1 | 2.54 [1.04 - 4.22] | 1.01 |
| IN | 0.57 [0.47 - 0.72] | 1.08 | 1.23 [0.25 - 2.26] | 1 | 1 [0.36 - 1.82] | 1.01 |
| IA | 0.51 [0.41 - 0.67] | 1.07 | 1.74 [0.04 - 5.45] | 1 | 3.32 [1.32 - 5.4] | 1.01 |
| KS | 0.57 [0.49 - 0.7] | 1.1 | 0.29 [0.01 - 1.47] | 1 | 0.23 [0.02 - 1] | 1.01 |
| KY | 0.54 [0.44 - 0.69] | 1.1 | 0.63 [0.02 - 1.96] | 1 | 0.76 [0.1 - 1.68] | 1.02 |
| LA | 0.55 [0.43 - 0.7] | 1.08 | 5.05 [2.57 - 7.68] | 1 | 5.16 [3.45 - 7.6] | 1 |
| ME | 0.6 [0.51 - 0.74] | 1.08 | 0.1 [0.01 - 0.36] | 1 | 0.08 [0.01 - 0.29] | 1 |
| MD | 0.52 [0.38 - 0.69] | 1.07 | 4.37 [0.16 - 7.41] | 1.01 | 5.02 [3.08 - 7.4] | 1.01 |
| MA | 0.57 [0.48 - 0.71] | 1.09 | 0.36 [0.01 - 1.57] | 1 | 0.29 [0.02 - 1.12] | 1.01 |
| MI | 0.53 [0.45 - 0.68] | 1.1 | 0.53 [0.02 - 2.28] | 1.01 | 0.72 [0.07 - 1.81] | 1.01 |
| MN | 0.5 [0.38 - 0.66] | 1.09 | 1.67 [0.08 - 3.39] | 1.01 | 2.51 [1.67 - 3.6] | 1.01 |
| MS | 0.53 [0.44 - 0.67] | 1.09 | 0.86 [0.02 - 4.09] | 1 | 1.65 [0.15 - 3.94] | 1.02 |
| MO | 0.57 [0.47 - 0.71] | 1.09 | 1.32 [0.65 - 2.07] | 1 | 1.19 [0.67 - 1.75] | 1.02 |
| MT | 0.53 [0.44 - 0.68] | 1.1 | 0.2 [0.01 - 0.87] | 1 | 0.29 [0.02 - 0.94] | 1.01 |
| NE | 0.49 [0.38 - 0.66] | 1.1 | 1.7 [0.04 - 5.25] | 1.01 | 3.58 [1.89 - 5.59] | 1.01 |
| NV | 0.51 [0.4 - 0.67] | 1.08 | 1.46 [0.03 - 4.48] | 1 | 2.71 [1.06 - 4.49] | 1.01 |
| NH | 0.62 [0.53 - 0.75] | 1.08 | 0.1 [0.01 - 0.39] | 1 | 0.08 [0.01 - 0.28] | 1 |
| NJ | 0.49 [0.38 - 0.68] | 1.08 | 4.35 [0.06 - 10.09] | 1.01 | 6.97 [4.57 - 9.94] | 1 |
| NM | 0.52 [0.41 - 0.68] | 1.08 | 0.8 [0.03 - 2.17] | 1 | 1.26 [0.38 - 2.23] | 1.02 |
| NY | 0.53 [0.4 - 0.69] | 1.06 | 14.11 [8.89 - 18.58] | 1 | 14.56 [11.3 - 18.41] | 1.02 |
| NC | 0.53 [0.44 - 0.67] | 1.1 | 0.45 [0.02 - 2.06] | 1 | 0.74 [0.04 - 1.95] | 1.01 |
| ND | 0.56 [0.44 - 0.72] | 1.07 | 0.38 [0.02 - 1.55] | 1 | 0.43 [0.03 - 1.57] | 1 |
| OH | 0.55 [0.46 - 0.69] | 1.1 | 0.49 [0.02 - 1.66] | 1.01 | 0.54 [0.05 - 1.36] | 1.02 |
| OK | 0.52 [0.43 - 0.66] | 1.11 | 0.43 [0.02 - 2.03] | 1.01 | 0.94 [0.1 - 2.16] | 1.02 |
| OR | 0.55 [0.43 - 0.71] | 1.07 | 1 [0.11 - 1.89] | 1 | 1.02 [0.42 - 1.81] | 1.01 |
| PA | 0.52 [0.36 - 0.69] | 1.08 | 5.21 [0.27 - 8.09] | 1.01 | 5.88 [3.96 - 8.22] | 1 |
| RI | 0.6 [0.5 - 0.74] | 1.09 | 0.21 [0.01 - 0.98] | 1 | 0.18 [0.02 - 0.78] | 1 |
| SC | 0.52 [0.43 - 0.66] | 1.09 | 0.66 [0.02 - 3.29] | 1 | 1.48 [0.17 - 3.37] | 1.02 |
| SD | 0.59 [0.48 - 0.73] | 1.08 | 0.24 [0.01 - 1.47] | 1 | 0.3 [0.02 - 1.42] | 1 |
| TN | 0.49 [0.39 - 0.64] | 1.09 | 0.79 [0.02 - 3.38] | 1 | 2.43 [0.99 - 4.02] | 1.02 |
| TX | 0.5 [0.4 - 0.64] | 1.09 | 0.98 [0.03 - 3.58] | 1 | 2.44 [1.01 - 4.16] | 1.02 |
| UT | 0.53 [0.44 - 0.67] | 1.11 | 1.07 [0.39 - 1.91] | 1.01 | 1.22 [0.66 - 1.93] | 1.01 |
| VT | 0.59 [0.5 - 0.74] | 1.08 | 0.09 [0.01 - 0.35] | 1 | 0.09 [0.01 - 0.3] | 1 |
| VA | 0.56 [0.45 - 0.72] | 1.06 | 1.25 [0.04 - 3.09] | 1 | 1.07 [0.1 - 2.4] | 1.01 |
| WA | 0.56 [0.46 - 0.72] | 1.08 | 0.96 [0.05 - 2.14] | 1 | 0.79 [0.12 - 1.66] | 1.01 |
| WV | 0.53 [0.43 - 0.68] | 1.1 | 0.37 [0.02 - 1.2] | 1 | 0.57 [0.1 - 1.23] | 1 |
| WI | 0.53 [0.44 - 0.67] | 1.1 | 0.34 [0.01 - 1.57] | 1 | 0.6 [0.05 - 1.75] | 1.01 |
| WY | 0.54 [0.43 - 0.69] | 1.09 | 0.38 [0.01 - 1.76] | 1 | 0.51 [0.04 - 1.85] | 1 |

**Table D.** Primary model posterior estimates of prevalence (undiagnosed and total) and seroprevalence as of December 31, 2020.

| **date** | **state** | **Undiagnosed Prevalence** | **Undiagnosed Prevalence trend** | **Total Prevalence** | **Total Prevalence trend** | **Seroprevalence** |
| --- | --- | --- | --- | --- | --- | --- |
| 12/31/20 | AK | 0.28% [0.14%-0.46%] | -0.09% | 0.73% [0.59%-0.92%] | -0.39% | 7.1% [6%-8.5%] |
| 12/31/20 | AL | 1.64% [0.63%-2.94%] | +0.1% | 2.35% [1.32%-3.67%] | +0.2% | 18.9% [14.7%-23.6%] |
| 12/31/20 | AR | 1.1% [0.51%-1.81%] | +0.18% | 1.76% [1.17%-2.48%] | +0.24% | 12.8% [10.6%-15.6%] |
| 12/31/20 | AZ | 1.03% [0.49%-1.68%] | +0.13% | 1.84% [1.29%-2.51%] | +0.23% | 12% [9.8%-14.9%] |
| 12/31/20 | CA | 0.94% [0.42%-1.61%] | -0.02% | 1.87% [1.34%-2.56%] | +0.36% | 9.8% [7.9%-12.3%] |
| 12/31/20 | CO | 0.44% [0.21%-0.67%] | -0.07% | 0.89% [0.65%-1.13%] | -0.26% | 9.7% [8.1%-11.9%] |
| 12/31/20 | CT | 0.48% [0.22%-0.79%] | +0.02% | 1.02% [0.74%-1.35%] | -0.01% | 8.3% [7.2%-9.9%] |
| 12/31/20 | DC | 0.34% [0.16%-0.54%] | +0.03% | 0.65% [0.47%-0.87%] | +0.05% | 9.1% [7.6%-11%] |
| 12/31/20 | DE | 0.79% [0.36%-1.29%] | +0.01% | 1.4% [0.98%-1.89%] | -0.03% | 12.1% [9.8%-15.1%] |
| 12/31/20 | FL | 0.73% [0.32%-1.3%] | +0.16% | 1.21% [0.78%-1.79%] | +0.28% | 11.6% [9.6%-14.1%] |
| 12/31/20 | GA | 1.32% [0.56%-2.17%] | +0.24% | 2.03% [1.17%-3.01%] | +0.56% | 16.4% [12.7%-20.1%] |
| 12/31/20 | HI | 0.14% [0.06%-0.25%] | +0.01% | 0.22% [0.14%-0.33%] | +0.02% | 2.9% [2.2%-4.1%] |
| 12/31/20 | IA | 1.08% [0.34%-2.2%] | +0.03% | 1.42% [0.65%-2.58%] | -0.03% | 23% [16.9%-30.4%] |
| 12/31/20 | ID | 1.01% [0.38%-1.95%] | -0.17% | 1.57% [0.96%-2.46%] | -0.32% | 16.1% [12%-21%] |
| 12/31/20 | IL | 0.55% [0.23%-0.92%] | -0.05% | 1.04% [0.72%-1.43%] | -0.18% | 14.8% [12.2%-18%] |
| 12/31/20 | IN | 0.62% [0.3%-1.07%] | -0.06% | 1.31% [0.99%-1.76%] | -0.2% | 11.5% [9.3%-14.1%] |
| 12/31/20 | KS | 0.99% [0.43%-1.67%] | -0.04% | 1.71% [1.09%-2.44%] | +0.08% | 14.3% [11.4%-17.7%] |
| 12/31/20 | KY | 0.9% [0.36%-1.58%] | +0.12% | 1.46% [0.9%-2.14%] | +0.08% | 10.4% [8.3%-12.9%] |
| 12/31/20 | LA | 0.73% [0.31%-1.37%] | +0.17% | 1.22% [0.79%-1.87%] | +0.21% | 16.1% [13.5%-19.3%] |
| 12/31/20 | MA | 0.54% [0.29%-0.83%] | +0.08% | 1.17% [0.92%-1.47%] | +0.16% | 7.3% [6.1%-8.8%] |
| 12/31/20 | MD | 0.49% [0.2%-1.02%] | +0.05% | 0.86% [0.57%-1.39%] | +0.02% | 13.1% [11%-16.1%] |
| 12/31/20 | ME | 0.28% [0.14%-0.45%] | +0.02% | 0.59% [0.44%-0.77%] | +0.13% | 2.1% [1.6%-2.7%] |
| 12/31/20 | MI | 0.44% [0.21%-0.7%] | -0.03% | 0.79% [0.55%-1.05%] | -0.23% | 10.2% [8.5%-12.1%] |
| 12/31/20 | MN | 0.45% [0.19%-0.8%] | -0.13% | 0.84% [0.57%-1.19%] | -0.5% | 15.1% [12.3%-19.3%] |
| 12/31/20 | MO | 0.55% [0.24%-0.97%] | -0.04% | 0.99% [0.66%-1.42%] | -0.09% | 11.6% [9.4%-14.6%] |
| 12/31/20 | MS | 1.29% [0.54%-2.28%] | +0.21% | 1.92% [1.16%-2.93%] | +0.29% | 16.9% [13.4%-21%] |
| 12/31/20 | MT | 0.54% [0.23%-0.92%] | -0.11% | 1.01% [0.69%-1.4%] | -0.29% | 12.4% [10%-15.4%] |
| 12/31/20 | NC | 0.85% [0.39%-1.39%] | +0.21% | 1.43% [0.92%-2.03%] | +0.42% | 9.8% [7.9%-11.9%] |
| 12/31/20 | ND | 0.31% [0.13%-0.58%] | -0.04% | 0.65% [0.47%-0.92%] | -0.38% | 15.9% [13.4%-19.7%] |
| 12/31/20 | NE | 0.71% [0.29%-1.25%] | -0.04% | 1.2% [0.77%-1.75%] | -0.28% | 18.5% [15%-22.9%] |
| 12/31/20 | NH | 0.43% [0.21%-0.7%] | +0.02% | 0.93% [0.7%-1.2%] | +0.08% | 3.6% [2.8%-4.5%] |
| 12/31/20 | NJ | 0.79% [0.29%-1.46%] | +0.07% | 1.31% [0.81%-2.01%] | +0.04% | 18.7% [15.4%-23%] |
| 12/31/20 | NM | 0.67% [0.3%-1.18%] | -0.12% | 1.31% [0.89%-1.87%] | -0.18% | 11.7% [9.3%-14.8%] |
| 12/31/20 | NV | 0.95% [0.39%-1.73%] | -0.07% | 1.62% [1.06%-2.4%] | -0.11% | 15.6% [12.4%-19.6%] |
| 12/31/20 | NY | 0.61% [0.31%-1.09%] | +0.14% | 1.12% [0.81%-1.61%] | +0.22% | 23.2% [19.7%-27.6%] |
| 12/31/20 | OH | 0.8% [0.36%-1.34%] | -0.06% | 1.5% [1.01%-2.08%] | -0.1% | 10% [8%-12.5%] |
| 12/31/20 | OK | 1.12% [0.5%-1.79%] | +0.03% | 1.96% [1.27%-2.71%] | +0.29% | 13.6% [10.9%-16.6%] |
| 12/31/20 | OR | 0.29% [0.12%-0.57%] | -0.01% | 0.54% [0.38%-0.82%] | -0.05% | 5.3% [4.2%-6.8%] |
| 12/31/20 | PA | 0.82% [0.32%-2%] | -0.15% | 1.46% [0.95%-2.66%] | -0.12% | 14.9% [12%-20.3%] |
| 12/31/20 | RI | 0.52% [0.28%-0.78%] | +0.01% | 1.29% [1.04%-1.56%] | -0.22% | 9.3% [7.9%-11%] |
| 12/31/20 | SC | 0.93% [0.44%-1.49%] | +0.2% | 1.51% [1%-2.09%] | +0.38% | 12.8% [10.4%-15.7%] |
| 12/31/20 | SD | 0.6% [0.25%-1.17%] | -0.11% | 1.15% [0.77%-1.76%] | -0.31% | 16.6% [12.8%-22.2%] |
| 12/31/20 | TN | 1.61% [0.67%-2.76%] | +0% | 2.8% [1.76%-4.06%] | +0.49% | 17.3% [13.6%-21.4%] |
| 12/31/20 | TX | 1.04% [0.48%-1.78%] | +0.04% | 1.59% [0.98%-2.41%] | +0.25% | 15.7% [12.3%-19.9%] |
| 12/31/20 | UT | 1.13% [0.52%-1.95%] | +0.16% | 1.84% [1.21%-2.69%] | +0.11% | 15.6% [12.4%-19.4%] |
| 12/31/20 | VA | 0.61% [0.24%-1.13%] | +0.1% | 0.99% [0.63%-1.49%] | +0.12% | 8.6% [6.8%-10.9%] |
| 12/31/20 | VT | 0.14% [0.07%-0.23%] | +0.03% | 0.28% [0.21%-0.37%] | +0.03% | 1.5% [1.2%-2%] |
| 12/31/20 | WA | 0.34% [0.14%-0.64%] | +0.07% | 0.57% [0.37%-0.88%] | -0.02% | 6.8% [5.3%-8.7%] |
| 12/31/20 | WI | 0.6% [0.26%-0.98%] | -0.08% | 1.11% [0.75%-1.51%] | -0.19% | 14.8% [12.2%-17.9%] |
| 12/31/20 | WV | 0.79% [0.37%-1.33%] | +0.03% | 1.41% [1%-1.94%] | +0.04% | 7.3% [5.7%-9.4%] |
| 12/31/20 | WY | 0.47% [0.22%-0.83%] | -0.15% | 0.98% [0.71%-1.35%] | -0.33% | 11.9% [9.4%-15.5%] |
| 12/31/20 | US | 0.83% [0.41%-1.25%] | +0.04% | 1.43% [0.99%-1.86%] | +0.12% | 13.2% [12.3%-14.2%] |

**Table E.** Geometric mean model posterior estimates of prevalence (undiagnosed and total) and seroprevalence as of December 31, 2020.

| **date** | **state** | **Undiagnosed Prevalence** | **Undiagnosed Prevalence trend** | **Total Prevalence** | **Total Prevalence trend** | **Seroprevalence** |
| --- | --- | --- | --- | --- | --- | --- |
| 12/31/20 | AK | 0.4% [0.4%-0.4%] | -0.12% | 0.85% [0.85%-0.85%] | -0.41% | 7% [6.4%-7.7%] |
| 12/31/20 | AL | 1.86% [1.86%-1.86%] | +0.12% | 2.57% [2.57%-2.57%] | +0.23% | 16.8% [14.9%-19.1%] |
| 12/31/20 | AR | 1.43% [1.43%-1.43%] | +0.24% | 2.09% [2.09%-2.09%] | +0.31% | 12.2% [11%-13.5%] |
| 12/31/20 | AZ | 1.3% [1.3%-1.3%] | +0.16% | 2.12% [2.12%-2.12%] | +0.28% | 11.3% [10.1%-13%] |
| 12/31/20 | CA | 1.1% [1.1%-1.1%] | -0.02% | 2.03% [2.03%-2.03%] | +0.36% | 8.9% [7.8%-10.1%] |
| 12/31/20 | CO | 0.55% [0.55%-0.55%] | -0.08% | 1% [1%-1%] | -0.26% | 9.2% [8.2%-10.3%] |
| 12/31/20 | CT | 0.67% [0.67%-0.67%] | +0.03% | 1.22% [1.22%-1.22%] | +0.03% | 8.2% [7.3%-9%] |
| 12/31/20 | DC | 0.37% [0.37%-0.37%] | +0.03% | 0.69% [0.69%-0.69%] | +0.06% | 8.5% [7.3%-10.3%] |
| 12/31/20 | DE | 0.82% [0.82%-0.82%] | +0.01% | 1.43% [1.43%-1.43%] | -0.03% | 10.8% [9.3%-12.7%] |
| 12/31/20 | FL | 0.88% [0.88%-0.88%] | +0.2% | 1.36% [1.36%-1.36%] | +0.32% | 10.9% [9.7%-12.2%] |
| 12/31/20 | GA | 1.31% [1.31%-1.31%] | +0.24% | 2.02% [2.02%-2.02%] | +0.55% | 14.1% [12.5%-16.2%] |
| 12/31/20 | HI | 0.18% [0.18%-0.18%] | +0.02% | 0.26% [0.26%-0.26%] | +0.01% | 2.9% [2.6%-3.6%] |
| 12/31/20 | IA | 1.13% [1.13%-1.13%] | +0.04% | 1.47% [1.47%-1.47%] | -0.02% | 19.8% [18%-22.4%] |
| 12/31/20 | ID | 1.65% [1.65%-1.65%] | -0.2% | 2.18% [2.18%-2.18%] | -0.38% | 17.3% [15.5%-19.4%] |
| 12/31/20 | IL | 0.62% [0.62%-0.62%] | -0.05% | 1.12% [1.12%-1.12%] | -0.18% | 13.6% [12.2%-15.3%] |
| 12/31/20 | IN | 0.91% [0.91%-0.91%] | -0.07% | 1.61% [1.61%-1.61%] | -0.2% | 11.6% [10.4%-12.8%] |
| 12/31/20 | KS | 1.55% [1.55%-1.55%] | -0.05% | 2.31% [2.31%-2.31%] | +0.17% | 15.5% [13.7%-17.5%] |
| 12/31/20 | KY | 1.15% [1.15%-1.15%] | +0.16% | 1.71% [1.71%-1.71%] | +0.13% | 9.8% [8.8%-10.9%] |
| 12/31/20 | LA | 0.95% [0.95%-0.95%] | +0.23% | 1.44% [1.44%-1.44%] | +0.28% | 15.5% [13.6%-17.9%] |
| 12/31/20 | MA | 0.75% [0.75%-0.75%] | +0.13% | 1.39% [1.39%-1.39%] | +0.21% | 7.3% [6.5%-8.2%] |
| 12/31/20 | MD | 0.54% [0.54%-0.54%] | +0.05% | 0.91% [0.91%-0.91%] | +0.03% | 12.3% [10.5%-14.7%] |
| 12/31/20 | ME | 0.49% [0.49%-0.49%] | +0.03% | 0.81% [0.81%-0.81%] | +0.16% | 2.5% [2.1%-2.8%] |
| 12/31/20 | MI | 0.53% [0.53%-0.53%] | -0.04% | 0.88% [0.88%-0.88%] | -0.23% | 9.4% [8.5%-10.6%] |
| 12/31/20 | MN | 0.44% [0.44%-0.44%] | -0.12% | 0.83% [0.83%-0.83%] | -0.49% | 13% [11.8%-14.3%] |
| 12/31/20 | MO | 0.84% [0.84%-0.84%] | -0.04% | 1.28% [1.28%-1.28%] | -0.07% | 12% [11%-13.2%] |
| 12/31/20 | MS | 1.53% [1.53%-1.53%] | +0.26% | 2.17% [2.17%-2.17%] | +0.35% | 15.6% [13.8%-17.8%] |
| 12/31/20 | MT | 0.66% [0.66%-0.66%] | -0.13% | 1.13% [1.13%-1.13%] | -0.29% | 11.4% [10.3%-12.5%] |
| 12/31/20 | NC | 1.01% [1.01%-1.01%] | +0.26% | 1.61% [1.61%-1.61%] | +0.49% | 8.9% [7.9%-10.1%] |
| 12/31/20 | ND | 0.44% [0.44%-0.44%] | -0.05% | 0.78% [0.78%-0.78%] | -0.38% | 15.6% [14.5%-17.1%] |
| 12/31/20 | NE | 0.68% [0.68%-0.68%] | -0.04% | 1.17% [1.17%-1.17%] | -0.28% | 16% [14.3%-18.2%] |
| 12/31/20 | NH | 0.82% [0.82%-0.82%] | +0.06% | 1.32% [1.32%-1.32%] | +0.14% | 4.4% [3.8%-5%] |
| 12/31/20 | NJ | 0.76% [0.76%-0.76%] | +0.06% | 1.28% [1.28%-1.28%] | +0.03% | 17.1% [14.7%-20.3%] |
| 12/31/20 | NM | 0.74% [0.74%-0.74%] | -0.14% | 1.38% [1.38%-1.38%] | -0.18% | 10.3% [9.2%-11.6%] |
| 12/31/20 | NV | 1.04% [1.04%-1.04%] | -0.07% | 1.7% [1.7%-1.7%] | -0.1% | 13.9% [12.2%-15.8%] |
| 12/31/20 | NY | 0.71% [0.71%-0.71%] | +0.17% | 1.22% [1.22%-1.22%] | +0.25% | 22.2% [19.1%-26.4%] |
| 12/31/20 | OH | 1.04% [1.04%-1.04%] | -0.07% | 1.76% [1.76%-1.76%] | -0.06% | 9.4% [8.4%-10.5%] |
| 12/31/20 | OK | 1.26% [1.26%-1.26%] | +0.04% | 2.11% [2.11%-2.11%] | +0.32% | 12% [10.8%-13.3%] |
| 12/31/20 | OR | 0.39% [0.39%-0.39%] | -0.01% | 0.64% [0.64%-0.64%] | -0.05% | 5.2% [4.5%-6%] |
| 12/31/20 | PA | 0.92% [0.92%-0.92%] | -0.16% | 1.56% [1.56%-1.56%] | -0.13% | 13.7% [11.7%-16.3%] |
| 12/31/20 | RI | 0.8% [0.8%-0.8%] | +0.02% | 1.57% [1.57%-1.57%] | -0.19% | 9.8% [8.9%-10.9%] |
| 12/31/20 | SC | 1.04% [1.04%-1.04%] | +0.22% | 1.63% [1.63%-1.63%] | +0.41% | 11.6% [10.2%-13.3%] |
| 12/31/20 | SD | 1.02% [1.02%-1.02%] | -0.14% | 1.6% [1.6%-1.6%] | -0.28% | 18.3% [16.4%-20.3%] |
| 12/31/20 | TN | 1.53% [1.53%-1.53%] | -0.01% | 2.71% [2.71%-2.71%] | +0.47% | 14.2% [12.7%-15.9%] |
| 12/31/20 | TX | 1.03% [1.03%-1.03%] | +0.04% | 1.59% [1.59%-1.59%] | +0.25% | 13.4% [11.9%-15.2%] |
| 12/31/20 | UT | 1.37% [1.37%-1.37%] | +0.2% | 2.09% [2.09%-2.09%] | +0.16% | 14.2% [12.9%-15.8%] |
| 12/31/20 | VA | 0.86% [0.86%-0.86%] | +0.15% | 1.23% [1.23%-1.23%] | +0.15% | 8.5% [7.5%-9.9%] |
| 12/31/20 | VT | 0.22% [0.22%-0.22%] | +0.05% | 0.37% [0.37%-0.37%] | +0.05% | 1.7% [1.5%-2%] |
| 12/31/20 | WA | 0.5% [0.5%-0.5%] | +0.1% | 0.73% [0.73%-0.73%] | +0.02% | 6.9% [6.1%-7.8%] |
| 12/31/20 | WI | 0.7% [0.7%-0.7%] | -0.09% | 1.22% [1.22%-1.22%] | -0.18% | 13.4% [12.3%-14.8%] |
| 12/31/20 | WV | 0.92% [0.92%-0.92%] | +0.04% | 1.54% [1.54%-1.54%] | +0.03% | 6.4% [5.7%-7.2%] |
| 12/31/20 | WY | 0.58% [0.58%-0.58%] | -0.18% | 1.09% [1.09%-1.09%] | -0.34% | 11.1% [9.9%-12.4%] |
| 12/31/20 | US | 0.93% [0.93%-0.93%] | +0.05% | 1.54% [1.54%-1.54%] | +0.14% | 12.1% [11.4%-12.9%] |

**Table F.** International seroprevalence data.

| **Nation/ Country** | **Min date** | **Max date** | **Point est. (%)** | **LCL (%)** | **UCL (%)** | **Source** |
| --- | --- | --- | --- | --- | --- | --- |
| Belgium | 3/30/20 | 4/5/20 | 2.9 | 2.4 | 3.5 | (18) |
| Belgium | 4/20/20 | 4/26/20 | 6 | 5.2 | 6.9 | (18) |
| Belgium | 5/18/20 | 5/25/20 | 6.9 | 6.1 | 7.8 | (18) |
| Belgium | 6/8/20 | 6/13/20 | 5.5 | 4.7 | 6.4 | (18) |
| Belgium | 6/29/20 | 7/4/20 | 4.5 | 3.8 | 5.3 | (18) |
| Canada | 5/19/20 | 6/18/20 | 0.7 | 0.6 | 0.8 | (19) |
| Denmark | 4/6/20 | 5/3/20 | 1.9 | 0.8 | 2.3 | (20) |
| England | 2/1/20 | 3/31/20 | 1.8 | 0.8 | 3.5 | (21) |
| England | 2/1/20 | 3/31/20 | 1.4 | 0.1 | 5.9 | (21) |
| England | 3/16/20 | 6/30/20 | 3.9 | 3.4 | 4.4 | (22) |
| England | 4/1/20 | 4/30/20 | 4.5 | 2.1 | 8.4 | (21) |
| England | 4/1/20 | 4/30/20 | 6.6 | 4.8 | 8.8 | (21) |
| England | 4/26/20 | 7/26/20 | 6.2 | 5.6 | 6.9 | (23) |
| England | 4/26/20 | 7/8/20 | 6.3 | 5.6 | 7.1 | (24) |
| England | 4/30/20 | 5/22/20 | 8.6 | 8 | 9.2 | (25) |
| England | 5/1/20 | 5/31/20 | 2.7 | 0.7 | 0.9 | (21) |
| England | 5/1/20 | 5/31/20 | 7.8 | 6.3 | 9.6 | (21) |
| England | 5/22/20 | 6/8/20 | 8.2 | 7.5 | 8.9 | (25) |
| England | 6/1/20 | 8/2/20 | 3.2 | 2.5 | 4.1 | (21) |
| England | 6/1/20 | 8/2/20 | 4.9 | 2.7 | 8.1 | (21) |
| England | 6/8/20 | 7/6/20 | 7.1 | 6.6 | 7.6 | (25) |
| England | 6/20/20 | 7/13/20 | 6 | 5.8 | 6.1 | (26) |
| England | 7/13/20 | 7/21/20 | 6.8 | 6.3 | 7.3 | (25) |
| England | 7/20/20 | 8/16/20 | 5.5 | 5 | 6 | (25) |
| Greece | 3/1/20 | 3/31/20 | 0.02 | 0 | 0.25 | (27) |
| Greece | 4/1/20 | 4/30/20 | 0.25 | 0.02 | 0.5 | (27) |
| Hungary | 5/1/20 | 5/16/20 | 0.68 | 0.5 | 0.85 | (28) |
| Iceland | 4/1/20 | 4/4/20 | 0.6 | 0.3 | 0.9 | (29) |
| India | 5/11/20 | 5/25/20 | 0.7 | 0.6 | 0.8 | (9) |
| Ireland | 6/22/20 | 7/16/20 | 1.7 | 1.1 | 2.4 | (30) |
| Luxembourg | 4/15/20 | 5/5/20 | 2.06 | 1.34 | 2.77 | (31) |
| Norway | 4/20/20 | 5/17/20 | 1.1 | 0.5 | 2 | (32) |
| Portugal | 5/21/20 | 7/8/20 | 1.9 | 1.4 | 2.5 | (33) |
| Russia | 6/10/20 | 6/10/20 | 14 | 13.9 | 14.1 | (34) |
| Slovenia | 4/20/20 | 5/1/20 | 0.15 | 0.03 | 0.47 | (35) |


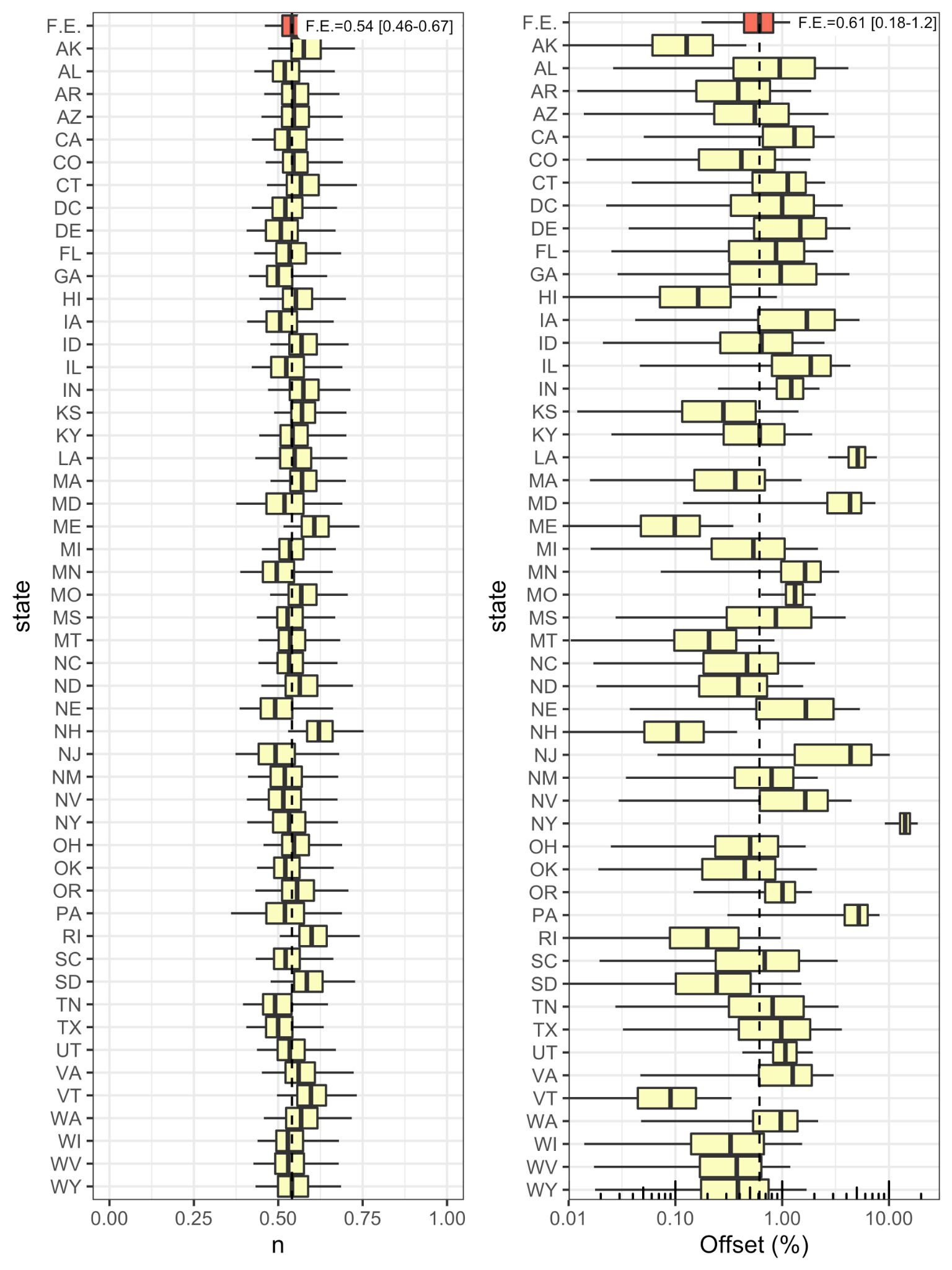


**Fig A:** Posterior distributions of the power parameter *n* and the seroprevalence offset SP_o_ for individual states using the primary random effects model. The fixed effect is denoted by “F.E.,” and the vertical dashed line represents its posterior median. For the simpler geometric mean model, the power parameter is fixed at *n*=½, and the F.E. posterior median [CrI] for SP_o_ is 0.90 [0.38-1.50].


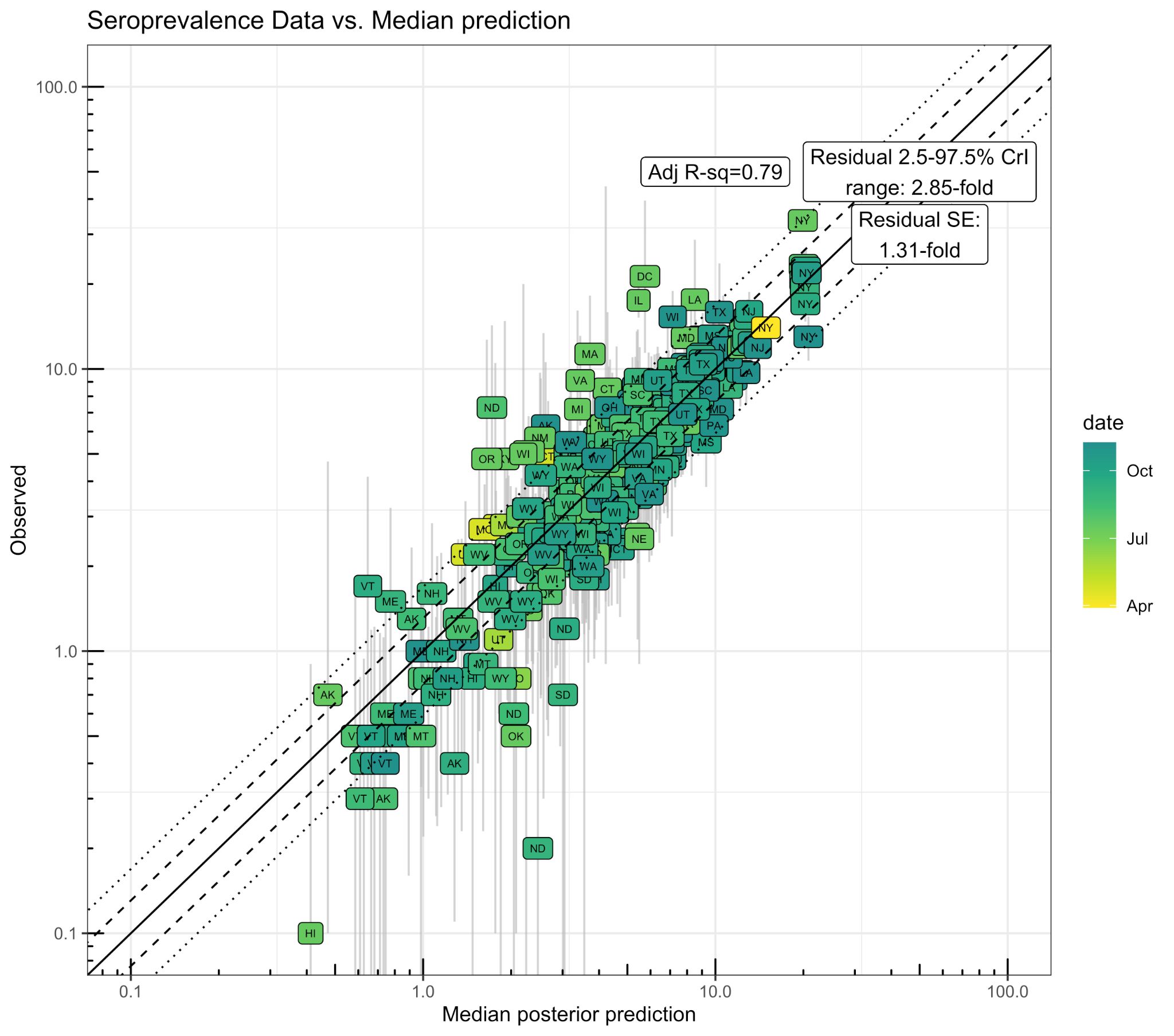


**Fig B:** Scatter plot of seroprevalence predictions (posterior median for primary random effects model) versus calibration data (reported point estimate and 95% CI). The solid line represents equality, the dashed line is +/- one residual standard error, and the dotted line is the 95% CrI residual error. The adjusted R^2^ is calculated from a linear model based on the log-transformed posterior medians and the observed point estimates. Results for the simpler geometric mean (*n*=½) model are similar, with residual SE of 1.33-fold, 95% CrI range of 3.01-fold, and adjusted R^2^ = 0.78.


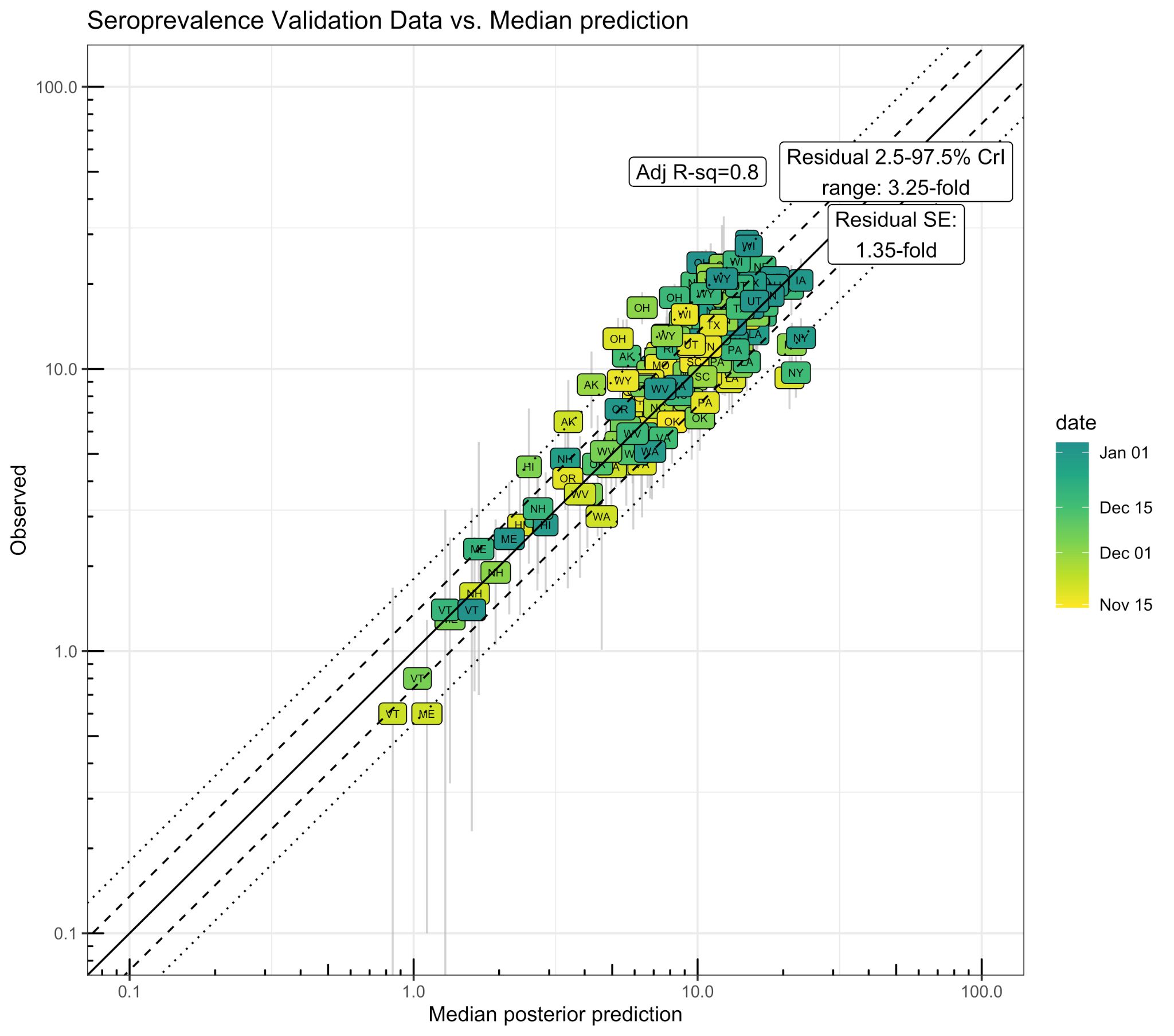


**Fig C:** Scatter plot of seroprevalence predictions (posterior median for primary random effects model) versus validation data (reported point estimate and 95% CI). The solid line represents equality, the dashed line is +/- one residual standard error, and the dotted line is the 95% CrI residual error. The adjusted R^2^ is calculated from a linear model based on the log-transformed posterior medians and the observed point estimates. Results for the simpler geometric mean (*n*=½) model are similar, with residual SE of 1.39-fold, 95% CrI range of 3.62-fold, and adjusted R^2^ = 0.77.


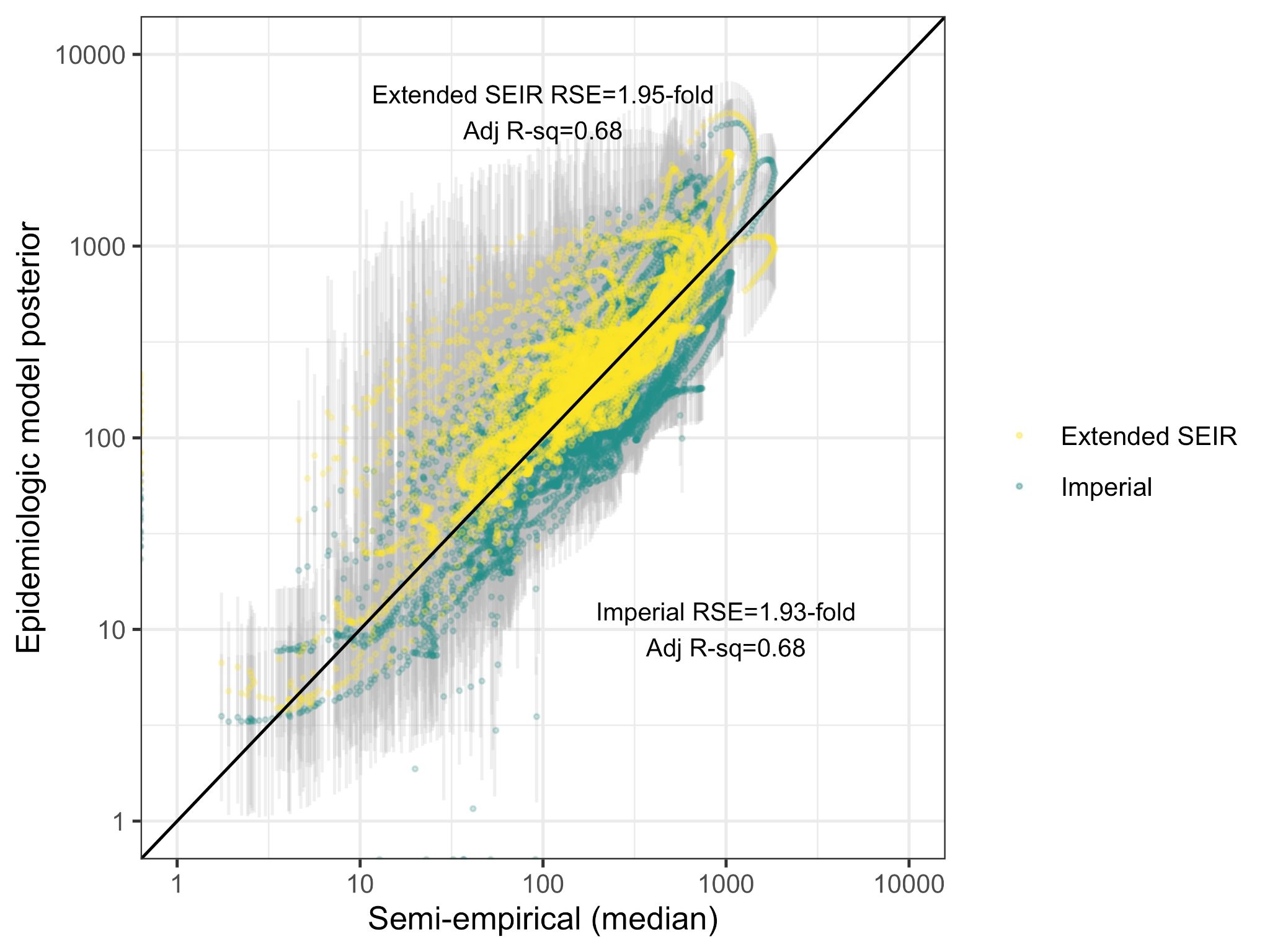


**Fig D:** Scatter plot of active infection prevalence predictions from semi-empirical model (posterior median for primary random effects model) versus those from epidemiologic models (posterior median and 95% CrI). The solid line represents equality. The residual standard error (RSE) and adjusted R^2^ are from the comparison of natural log-transformed median predictions. Results for the simpler geometric mean (*n*=½) model are similar, with RSEs of 1.71-fold and 2.01-fold, 95% CrI ranges of 1.77-fold and 2.01-fold, and adjusted R^2^ values = 0.73 and 0.71, for the Extended SEIR and Imperial models, respectively.


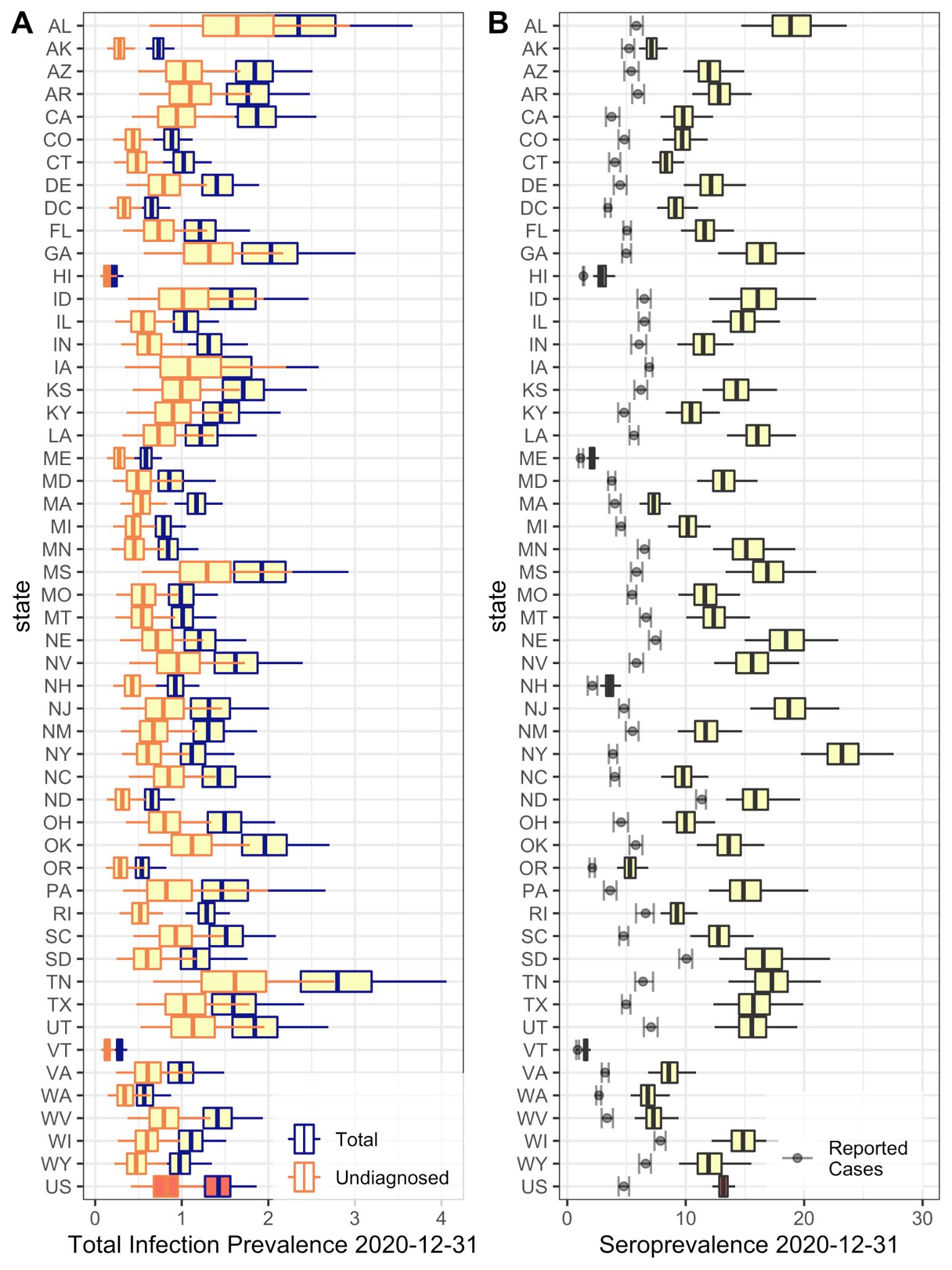


**Fig E:** Boxplots (box=IQR, line=median, whiskers=95% CrI) of posterior estimate of infection prevalence (A) and seroprevalence (B) across states and for the U.S. overall as of December 31, 2020, using the primary random effects model. In (B), for comparison, cumulative reported cases are shown with a 14-day lag to allow time for seroconversion (error bars denote range of 7-21 day lags).


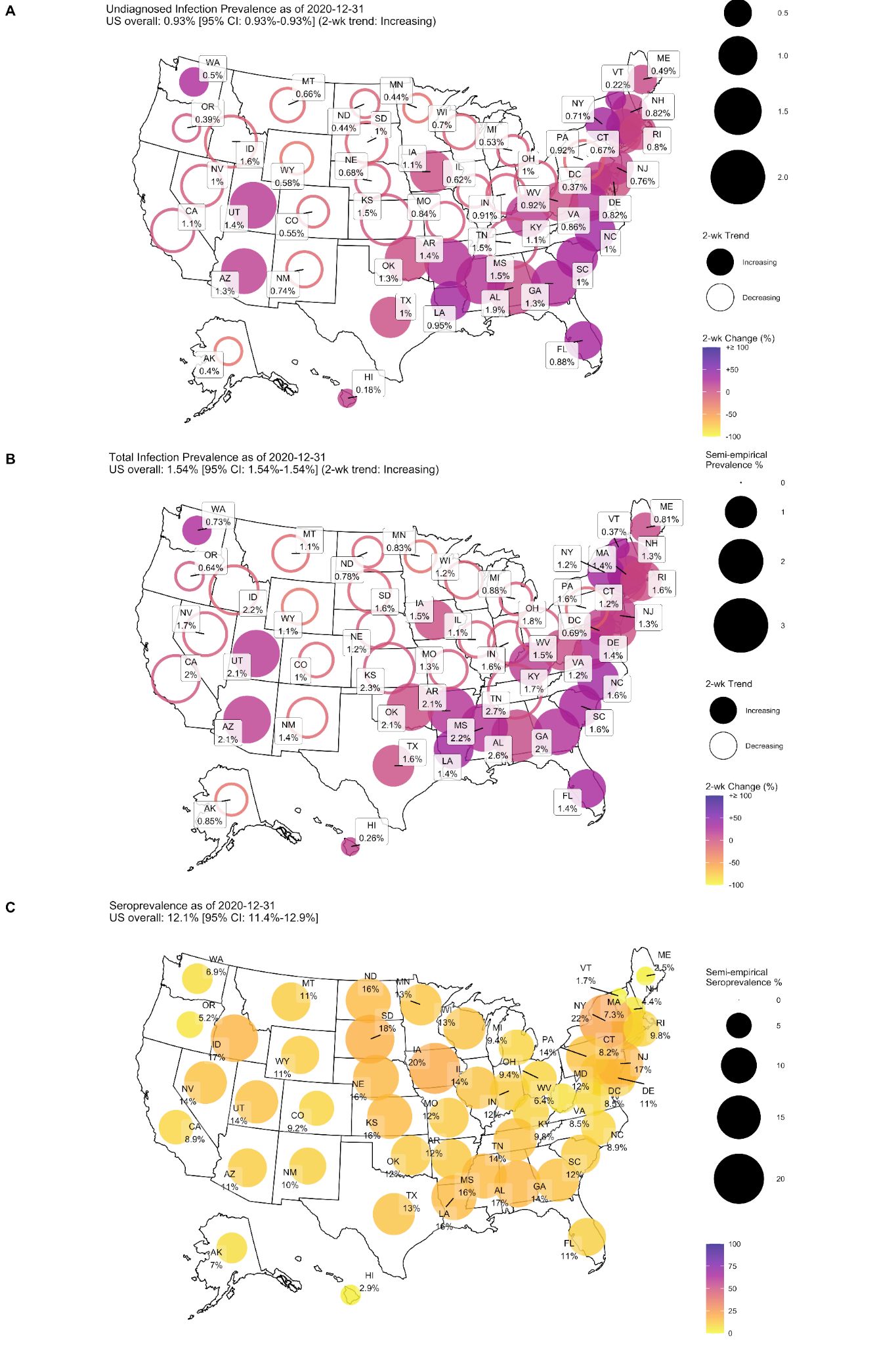


**Fig F.** A) Map of estimated undiagnosed (A) and total (B) prevalence and transmission trends and overall seroprevalence (C) as of December 31, 2020, based on data through January 15, 2021. Values based on the simpler geometric mean model (see Figure 4 for primary random effects model predictions). The maps were generated using the R package usmap <https://cran.r-project.org/web/packages/usmap/index.html> (GPL-3), which uses shape files from the U.S. Census Bureau (the link provided in documentation is here: <https://www.census.gov/geographies/mapping-files/time-series/geo/tiger-line-file.html>).


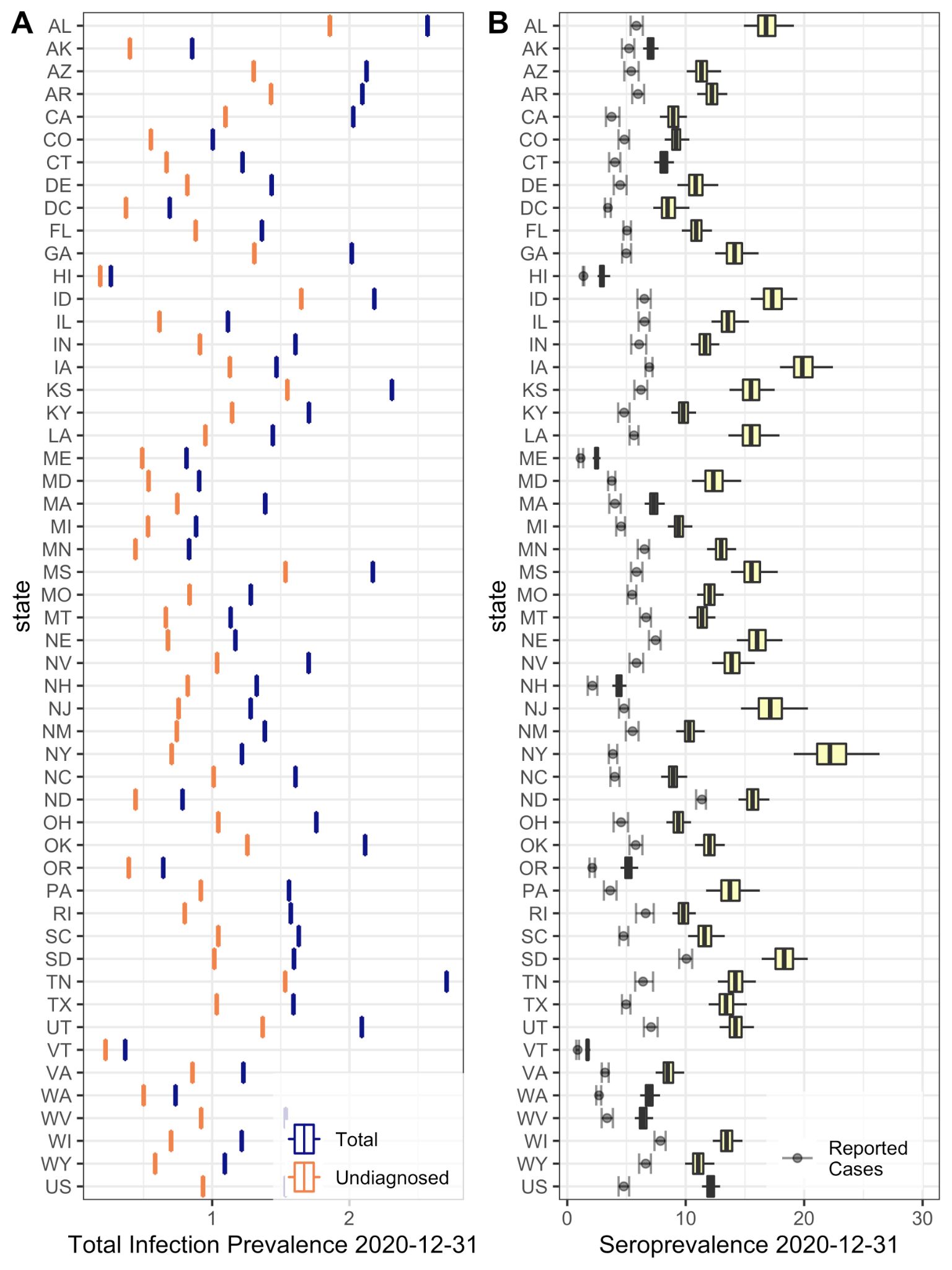


**Fig G:** Boxplots (box=IQR, line=median, whiskers=95% CrI) of posterior estimate of infection prevalence (A) and seroprevalence (B) across states and for the U.S. overall as of December 31, 2020, using the simpler geometric mean model. In (B), for comparison, cumulative reported cases are shown with a 14-day lag to allow time for seroconversion (error bars denote range of 7-21 day lags).


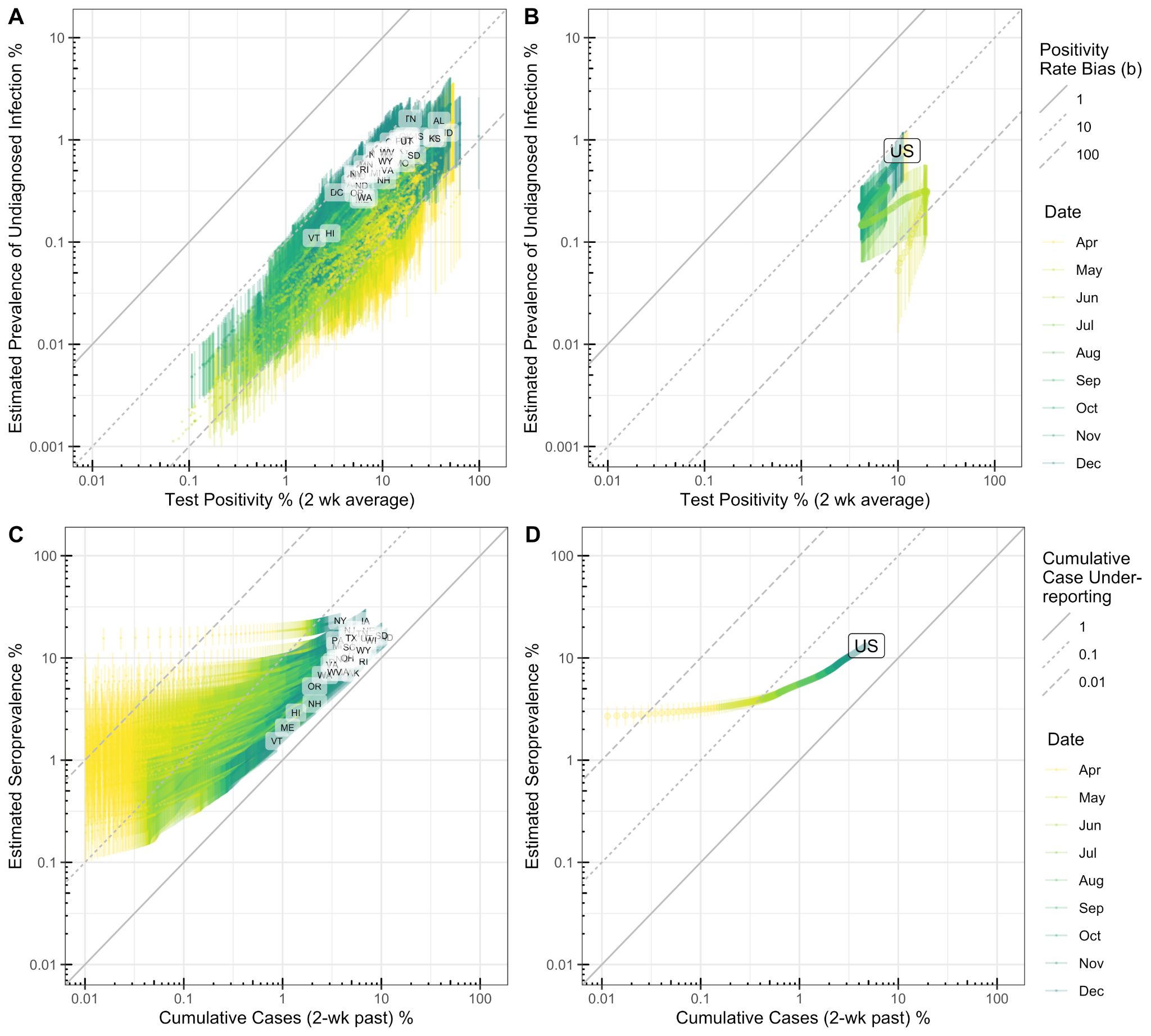


**Fig H.** Bias estimates from primary random effects model. A, B) Comparison of test positivity (14-day average) and semi-empirical prevalence estimates (median and 95% CrI) across all states (A) or across the U.S. in aggregate (B) from April 1-December 31, 2020. Diagonal lines denote different levels of positivity bias, as illustrated in Figure 1A. C, D) Comparison of cumulative reported cases, with 14-day lag to allow for conversion to seropositivity, and semi-empirical seropositivity estimates (median and 95% CrI) across all states (C) or across the U.S. in aggregate (D) from April 1-December 31, 2020. Diagonal lines denote different levels of cumulative case under-reporting. Results for the simpler geometric mean (*n*=½) model are similar.

**
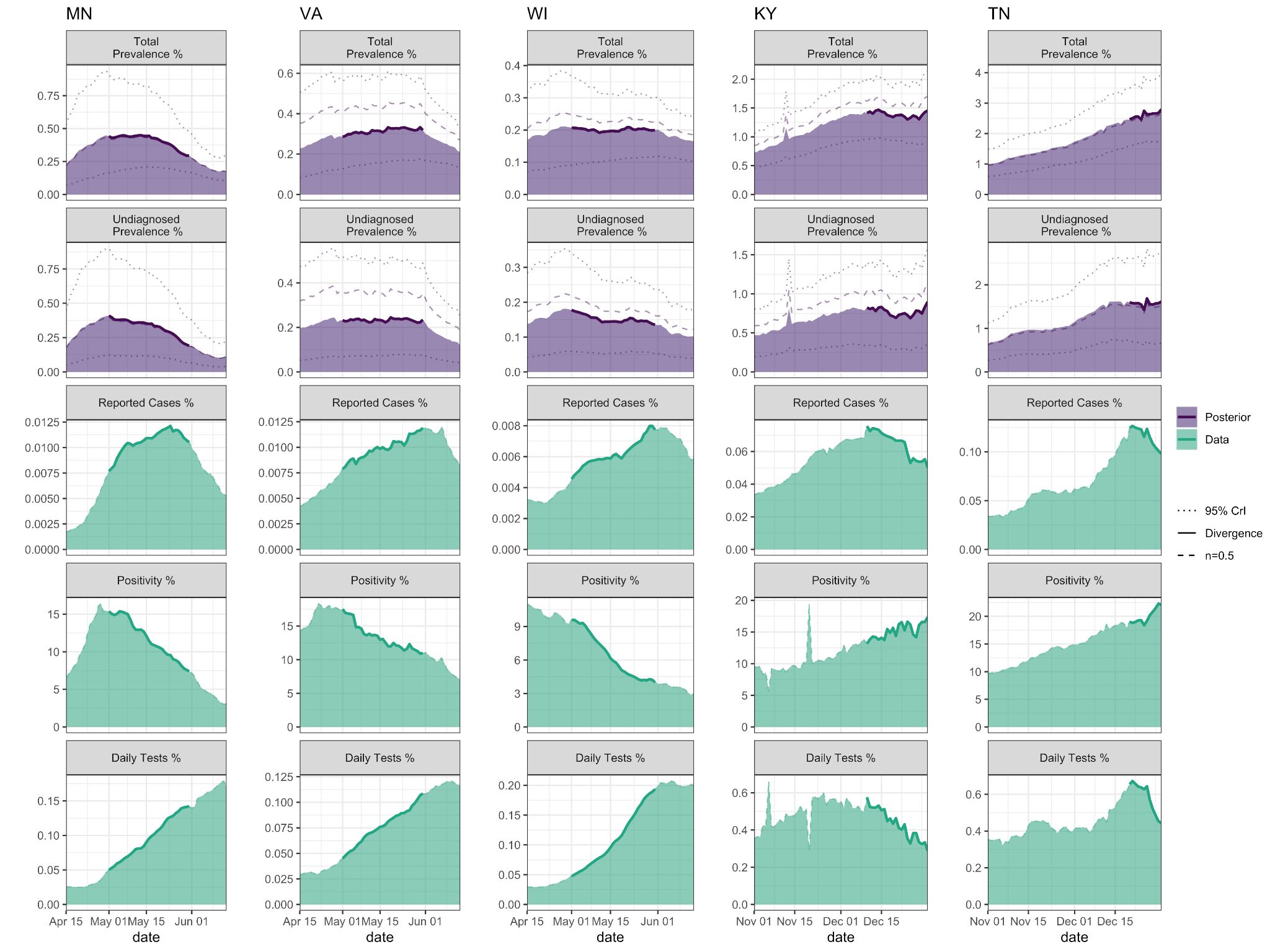
**

**Fig I:** Examples of five states where the trends in reported case rates and positivity rates diverged (i.e., one increasing, the other decreasing). For each state, the top panel is the active infection (total diagnosed and undiagnosed) prevalence as predicted by the semi-empirical model (posterior median and 95% CrI), the second panel is the active undiagnosed infection prevalence, whereas the bottom three panels show the reported case, positivity, and testing rates, each averaged over the previous 14 days.


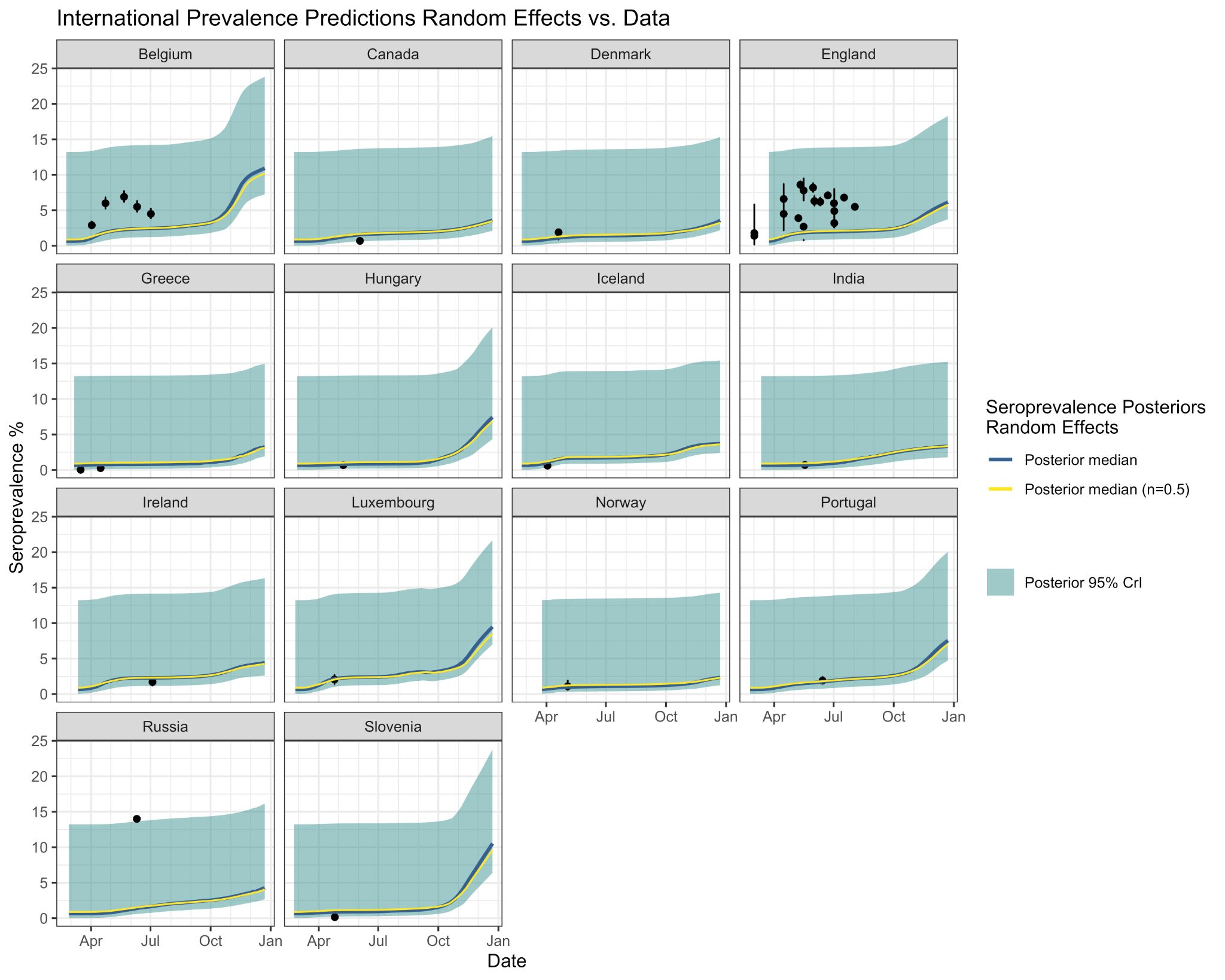


**Fig J:** Application of semi-empirical model using random effects posterior distributions from U.S. states to other nations/countries. COVID-19 antibody seroprevalence estimates (posterior median and 95% credible intervals) for each nation/country with state-wide seroprevalence data (**Table F**, reported point estimates and 95% confidence intervals shown).


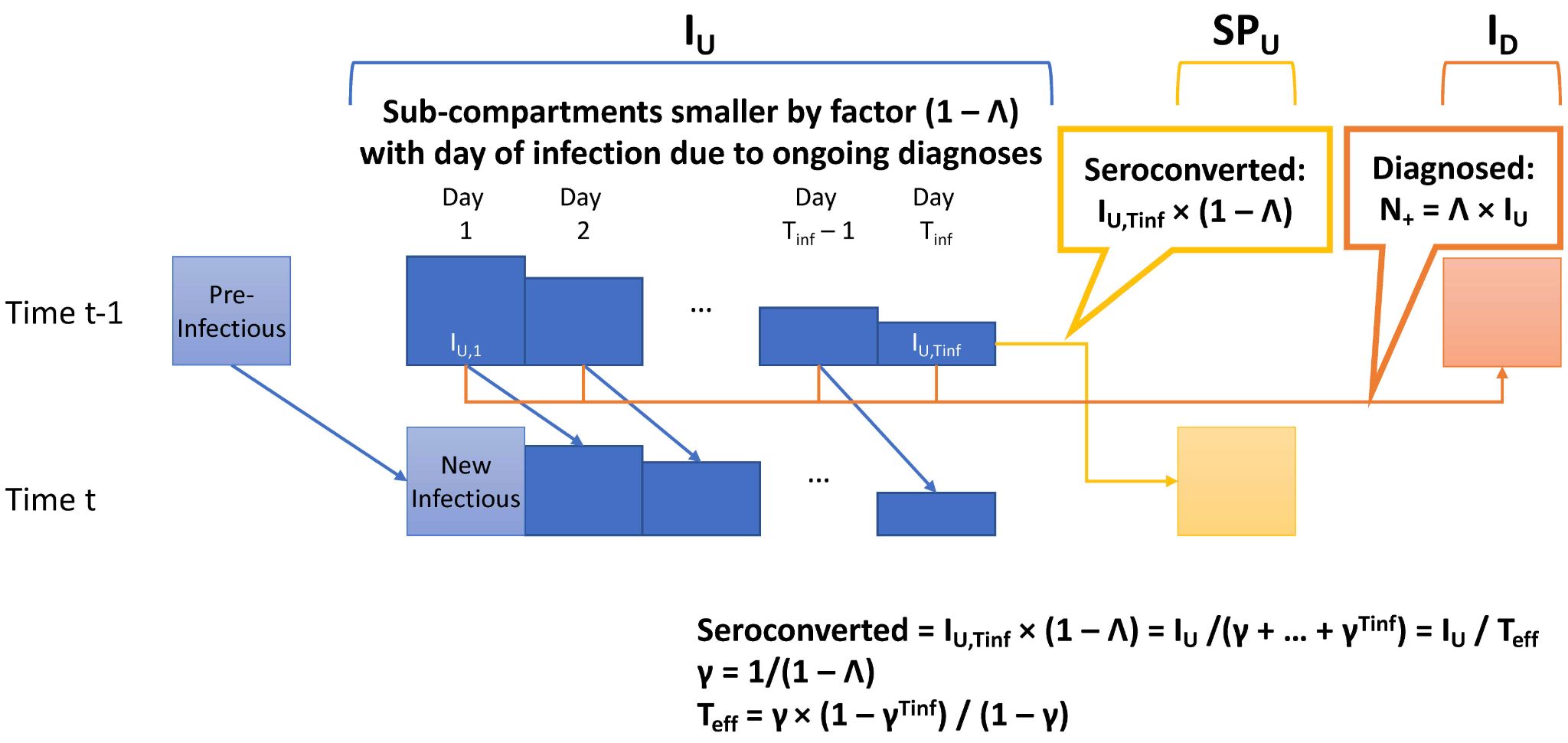


**Fig K:** Conceptual model of undiagnosed prevalence (equations 7-9). Assuming a time interval between infection and seropositivity = *T*_inf_, each time point *t*, we can subdivide the undiagnosed infection prevalence *I_U_* into *T*_inf_ “subcompartments” *I_U,m_* (*m* = 1...*T*_inf_). The number of undiagnosed individuals who are diagnosed each day is *I_U_* ✕ Λ (diagnosis considered sampling without replacement of *I_U_*). The number of undiagnosed individuals who become newly undiagnosed seropositive (entering SP_U_ the next day) is simply the number in the last subcompartment $I_{U,T_{inf}}$multiplied by another factor of (1 – Λ) to account for the fraction that get diagnosed that day.


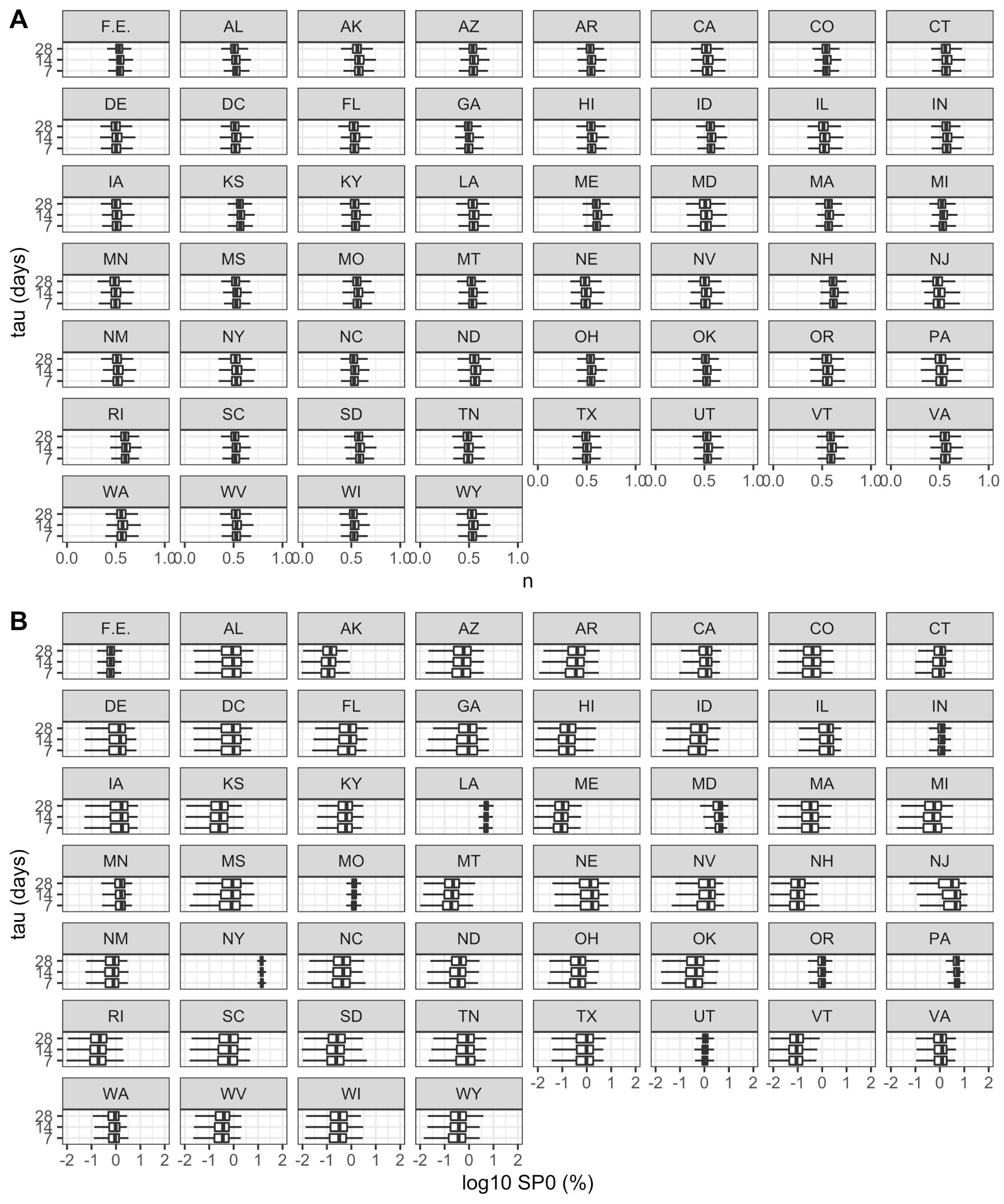


**Fig L: Sensitivity of parameter estimates to changing averaging time τ from 14 to 7 or 28 days.** A) Posterior distributions of power parameter n; B) posterior distributions of seroprevalence offset SP_o_.


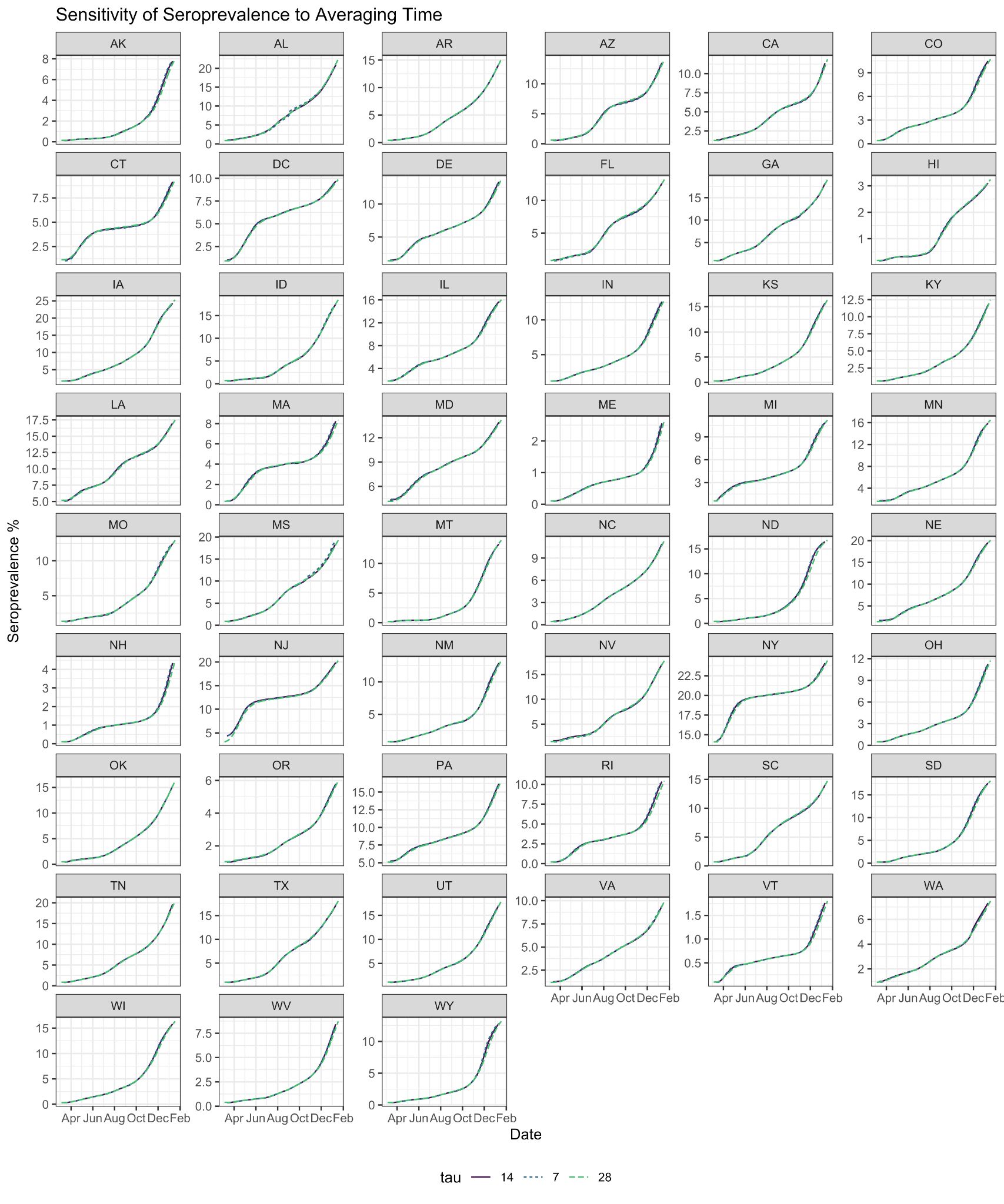


**Fig M: Sensitivity of seroprevalence predictions to changing averaging time τ from 14 to 7 or 28 days.** All predictions are posterior medians.


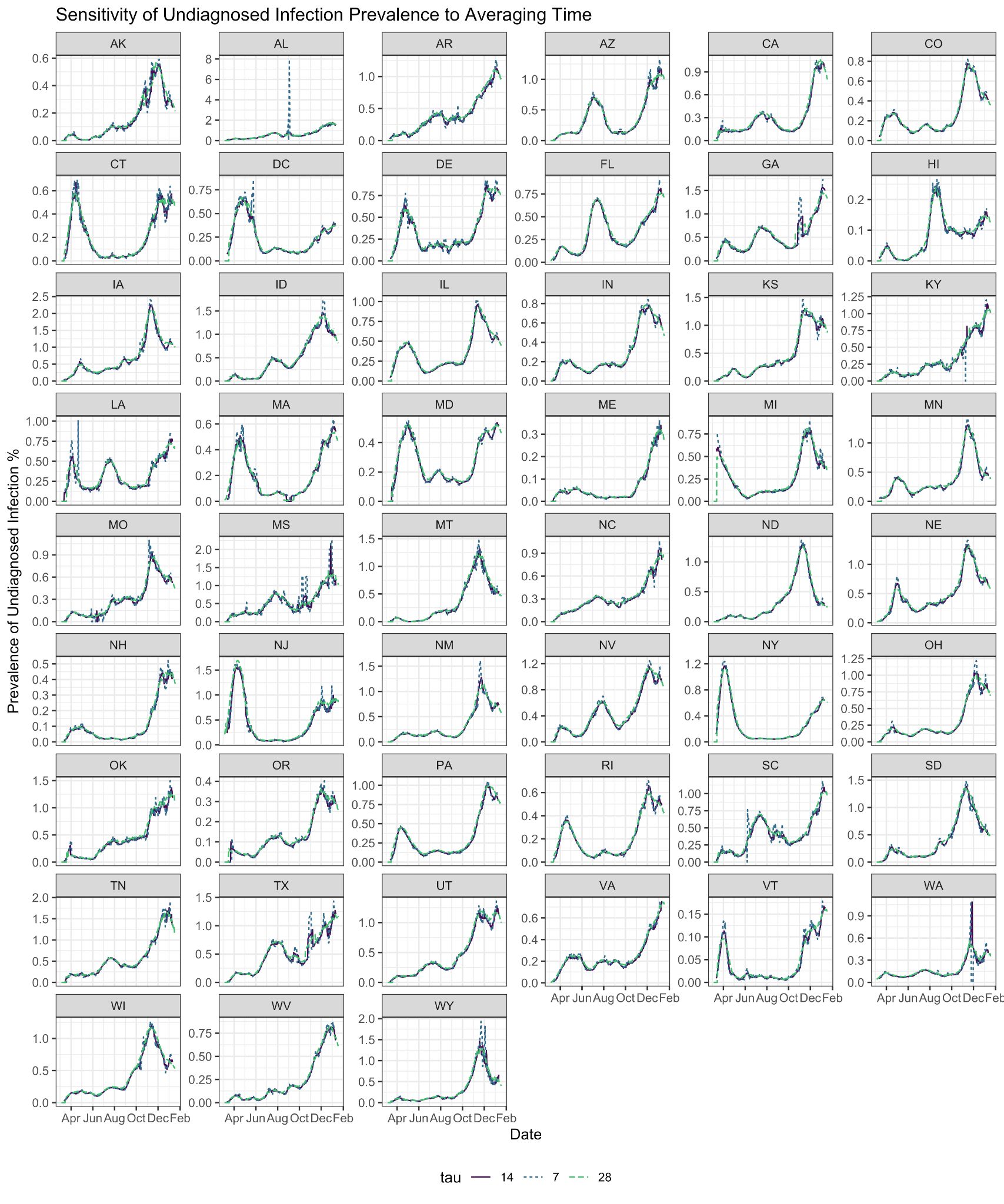


**Fig N: Sensitivity of undiagnosed prevalence predictions to changing averaging time τ from 14 to 7 or 28 days.** All predictions are posterior medians.

**
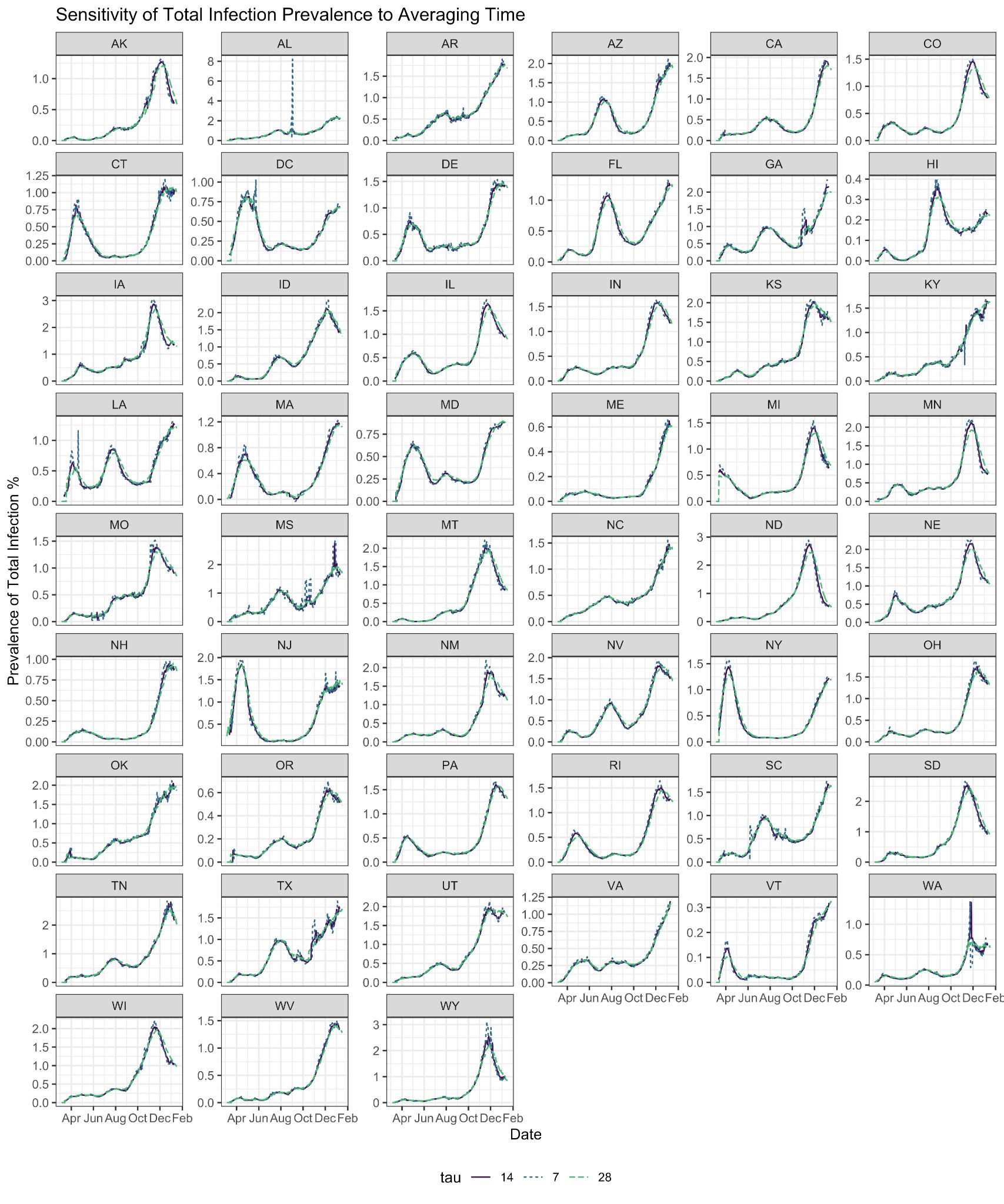
**

**Fig O: Sensitivity of total prevalence predictions to changing averaging time τ from 14 to 7 or 28 days.** All predictions are posterior medians.
